# Age Effects on Distraction in a Visual Task Requiring Fast Reactions: An Event-Related Potential Study

**DOI:** 10.3389/fnagi.2020.596047

**Published:** 2020-11-26

**Authors:** Petia Kojouharova, Zsófia Anna Gaál, Boglárka Nagy, István Czigler

**Affiliations:** ^1^Institute of Cognitive Neuroscience and Psychology, Research Centre for Natural Sciences, Budapest, Hungary; ^2^Doctoral School of Psychology (Cognitive Science), Budapest University of Technology and Economics, Budapest, Hungary; ^3^Institute of Psychology, Eötvös Loránd University, Budapest, Hungary

**Keywords:** visual distraction, non-spatial distraction, aging, event-related potentials (ERP), choice reaction time, Go/Nogo

## Abstract

We investigated the effects of distractors in older and younger participants in choice and simple reaction time tasks with concurrent registration of event-related potentials. In the task the participants had to prevent a disk from falling into a bin after a color or luminosity change (target stimuli). Infrequently, task-irrelevant stimuli (schematic faces or threatening objects) were superimposed on the target stimuli (distractors), or the bin disappeared which required no response (Nogo trials). Reaction time was delayed to the distractors, but this effect was similar in the two age groups. As a robust age-related difference, in the older group a large anterior positivity and posterior negativity emerged to the distractors within the 100–200 ms post-stimulus range, and these components were larger for schematic faces than for threatening objects. sLORETA localized the age-specific effect to the ventral stream of the visual system and to anterior structures considered as parts of the executive system. The Nogo stimuli elicited a late positivity (Nogo P3) with longer latency in the older group. We interpreted the age-related differences as decreased but compensated resistance to task-irrelevant change of the target stimuli.

## Introduction

In modern life older adults frequently face the same challenges as younger adults. The most frequently cited examples are traffic situations that frequently require fast reactions from both drivers and pedestrians. Other, potentially less harmful examples from everyday life with similar requirements are hand fitting and kitchen activities. In this study our aim was to compare older and younger participants in tasks requiring both fast reactions in the presence of rare irrelevant (distractor) events and occasional refraining from reacting. In other words, in situations when some events are connected to the motivated behavior, other events may also compete for the resources of processing capacities (Desimone and Duncan, 1995). If these distractor events are unexpected and salient, they may cause performance to deteriorate, and elicit brain activity specific to the processing and inhibiting of the distracting stimuli.

Age-related changes in the sensitivity to distracting stimuli are frequently investigated in the visual and auditory modality. Generally, older adults are viewed as more vulnerable to task-irrelevant events than younger adults (e.g., Rabbitt, 1965; Hasher and Zacks, 1988; Nagy et al., 2020). However, distraction has several forms, and distraction as well as resisting distraction can vary in different modalities and situations. In the present study we investigate the distraction effect of rare irrelevant stimuli superimposed on visual target stimuli. Besides the behavioral measures we recorded event-related potentials (ERPs). ERPs have high resolution in the time domain, and various ERP components are indexes of sub-processes of stimulus evaluation, response organization, or inhibition. Accordingly, ERP data are particularly useful in disclosing the stream of processes leading either to compromised performance or to processes for compensating for the potential effects of distractors. Importantly, similar performance of older and younger people can be reached by different brain processes. ERP data can shed light on such processing differences.

### Studies of Age-Related Distraction in the Visual Modality

We investigated age-related differences in non-spatial visual distraction, which is less frequently investigated than spatial visual distraction, but more frequently investigated in the auditory modality. Evidence for such differences in spatial attention has been equivocal. In a version of the spatial cuing task (Posner, 1980), Juola et al. (2000) obtained a larger distractor effect to peripheral stimulus onset distractors (invalid peripheral cuing) in older adults, but no age-related difference appeared to invalid central cuing. Similarly, Pratt and Bellomo (1999) obtained a larger effect of onset but not color distractors in older participants. On the contrary, Tales et al. (2002) found no age-related differences to either peripheral or central cues.

In a color and shape search task with spatial cuing Mertes et al. (2017) used both behavioral and ERP measures. They obtained a larger reaction time (RT) increase in the older group only in the color search task with invalid cuing. In the cue-target congruency condition (color target and color cue) the ERP distractor effect was larger in the older group as indicated by the attention-related N2pc. Furthermore, an earlier ERP signature of the distractor effect, the early distractor positivity (Pd-early), was absent in the older group. In a search task using the oculomotor capture paradigm Colcombe et al. (2003) found no age-related differences for onset and color distractors in either saccade measures or RT. Similarly, Lien et al. (2011) obtained no age-related differences for onset and color distractors when they measured RT and the N2pc component. On the other hand, in a search task Madden et al. (2014) obtained a larger effect of the salient (color) distractor in older participants. In this study frontal fMRI activation increased in distractor trials, and the activation was larger in the older group, but they reported no age-specific loci of the activity difference.

In a spatial attention paradigm, Wascher et al. (2012) also investigated the age-related effect of distractors at different locations on performance together with ERP activity. Stimuli (vertical or horizontal bars with higher or lower luminance than the background) were presented at two lateral positions. The task was either luminance matching within stimulus pairs (a less salient feature) or orientation matching within stimulus pairs (a more salient feature). Participants had to ignore the task-irrelevant stimulus dimension. In the conflicting condition (relevant and irrelevant change together) performance was disproportionally lower in the older group. There were only small age-related effects on earlier ERP components (N1 range), whereas components related to the activity of control structures (indicated by the frontocentral N2) were influenced to a greater extent by the salient distractors in the older group. According to the authors these results showed delayed activity of the control structures in the older group. Karthaus et al. (2018) investigated RT (braking) and the frontal early component P2 in both visual spatial and cross-modal distraction in a driving simulation. Braking slowed down in the older group in the presence of visual distraction. P2 was generally larger in the younger group.

Overall, research on spatial distraction in vision suggests that although age-related differences in processing distractors are not consistently observed in behavioral data, attention- and/or control-related processes may be involved to a greater extent in older adults.

### Studies of Age-Related Distraction in Cross-Modal Tasks

Cross-modal tasks (auditory distractors accompanying visual tasks) have been used as another effective way of studying distraction (see e.g., Escera et al., 2003), and older adults seem to be disproportionately affected. In the cross-modal condition in Karthaus et al.’s (2018) study braking did not slow down, but P2 increased in both groups to the acoustic distraction. P2 was again larger in the younger group. Parmentier et al. (2018) re-analyzed the data of four studies that included older and younger groups (Andrés et al., 2006; Parmentier and Andrés, 2010; Leiva et al., 2015, 2016). According to this analysis older participants were slower even beside the effect of general age-related slowing (e.g., Salthouse, 1996). In another version of the cross-modal task Cid-Fernández et al. (2014) obtained slower RT in the distractor trials in middle-aged and older groups. They also measured ERPs and reported disproportionately longer N2b latency in the middle-aged and older participants, indicating a later onset of orientation-related processes. In a subsequent study Cid-Fernández et al. (2016) concentrated on response-related ERP components and the late positivity (P3b). As their data showed, the distraction effect on the behavioral measures did not differ notably between the groups. However, as within trials the processing of the distractor stimuli proceeded, the distance between the various response-related components increased at a larger rate in the older group, indicating a strategic processing difference between the younger and older adults. The authors interpreted this finding as the age-related difference being due to a more extensive serial, in contrast to parallel, type of processing in the older groups. However, regarding the technical realization of the cross-modal method, Parmentier et al. (2018) noted that this paradigm has a spatial attention aspect, i.e., the visual stimuli were presented in the center of the screen at a distance, whereas the auditory stimuli were presented via headphones, thus their location was the same as the participant’s. Nevertheless, cross-modal research on distraction provides some evidence that processing distractors requires additional resources in older adults.

### Studies of Age-Related Distraction in the Auditory Modality

In tasks that use non-spatial distraction such as a purely auditory task (e.g., Schröger and Wolff, 1998) the age-related results are again equivocal. In this task participants have to discriminate between two values of a tone feature (usually duration), and infrequently there is a change in a task-irrelevant feature (usually pitch) as distractor. Mager et al. (2005) obtained no distraction difference between a younger and middle-aged group in RT, but error rate to the deviant stimuli (stimuli with shorter duration) was larger in the middle-aged participants. At the level of ERPs, mismatch negativity (MMN) and P3a effects appeared in both groups, but the P3a latency was longer in the middle-aged participants, and reorientation negativity (RON) was absent to the deviant short stimuli. Horváth et al. (2009) obtained no behavioral differences between younger and older adults in the same task, but both the late positivity and the RON were delayed in the older group. Berti et al. (2013) found a larger distraction effect (increased RT) in an older group compared to middle aged and younger groups. However, they obtained reliable age-effects only on the MMN amplitude. Accordingly, the age-related distraction difference was not particularly striking either on the behavioral or on the ERP level. Berti and Schröger (2006) developed a visual version of this task with duration discrimination and spatial displacement as distractor. In this paradigm Leiva et al. (2015) obtained similar results in an older and in a younger group. Note that the experiment had a spatial aspect, because the frequent and infrequent stimuli appeared at different locations.

Recently in a new version of the auditory task (gap detection preceded by occasional frequency changes (Volosin et al., 2017), the N1 amplitude reduction was measured as an indicator of distraction. According to the behavioral results, the effect of distractors had a longer duration in the older group, but this variable had no effect in younger participants. Meanwhile the older group maintained a high detection rate even at shorter glide-gap separations, indicating the possibility of attentional compensation for distraction.

### The Rationale and Expectations of the Present Study

In our study we presented frequent and infrequent events in the same location. Participants had to prevent the fall of a ‘disk’ into a ‘bin’ within a time limit. Infrequently, task-irrelevant distractors were superimposed on the target stimuli. In Experiment 1 and 2 the distractors were schematic faces. Because it was possible that the distractor-related ERP changes were specific to face-specific components, in Experiment 1a the distractors were threatening objects. In Experiment 1 and 1a the task was a choice RT, and to reduce task demand, in Experiment 2 it was changed to a simple RT task. Furthermore, we introduced infrequent Nogo trials in all tasks to compare the effects of two different inhibitory processes, distractor inhibition and response inhibition to task-irrelevant environmental changes.

Nogo stimuli may elicit the Nogo N2 component, an activity located within the anterior cingulate (ACC) area. The Nogo N2 is supposed to be a signature of top–down inhibition mechanisms to suppress incorrect response tendencies (Falkenstein et al., 1999; Bokura et al., 2001), or a correlate of conflict monitoring (Nieuwenhuis et al., 2003; Randall and Smith, 2011). A later component, the Nogo P3, is likely related to motor activity without execution (Bruin et al., 2001; Burle et al., 2004), but not to the inhibition itself (Randall and Smith, 2011). Although the precise cognitive processes of Nogo N2 and Nogo P3 components are unclear, they are connected to inhibitory control mechanisms (Huster et al., 2013). In a visual Nogo task Falkenstein et al. (2002) obtained a smaller N2 and a delayed anterior P3 in an older group. The Nogo stimuli in the present study did not require target processing, therefore these stimuli were ‘irrelevant’ Nogo stimuli as seen in Hsieh et al. (2016). In their study the infrequent Nogo stimuli did not elicit a longer and smaller anterior N2 in the older group, but the anterior P3 had a longer latency in the older group.

Concerning distraction effects *per se*, and age-related distraction differences specifically, in the early (100–200 ms) range of ERPs we had no *a priori* expectations. In general, infrequent stimuli are expected to elicit a larger posterior N1/N170 than the frequent ones. First, pattern onset (i.e., the superimposed distractors) elicits larger negativity (e.g., Clark et al., 1994). Second, because we used facial stimuli, they could have elicited the N170 component (e.g., Sagiv and Bentin, 2001). The posterior N1 is usually larger in younger participants (for a review see Čeponienė et al., 2008); furthermore, the alerting effect on N1 is larger in younger participants (Kaufman et al., 2016; Williams et al., 2016). However, in Experiment 1 the processing of the infrequent face stimuli may interfere with the processing of the task-related aspects. The more efficient inhibitory activity in the younger group may result in a different age-related activity of processes underlying the N1 amplitude.

As for attentional ERP effects following the N1/N170 range, in discrimination paradigms a posterior negativity (selection negativity) emerges (e.g., Harter and Guido, 1980; Wijers et al., 1989; Czigler and Csibra, 1990; Kenemans et al., 1993; Potts, 2004). In the later part of the selection negativity Kenemans et al. (1995) obtained a reduced amplitude to non-target stimuli in an older group. More recently, in a letter-color discrimination task Alperin et al. (2013) obtained a reduced selection negativity in highly functioning older participants. However, it is uncertain whether salient but task-irrelevant stimuli automatically elicit this component. Together, on the basis of these findings larger or longer posterior negativity is expected in the younger group to the infrequent events.

Anterior ERP components that could possibly emerge in the present study are attributed to attentional (selection positivity) and/or central executive processes. An attention-related positivity in the 150–250 ms (P2) range (selection positivity) has been connected to the detection of particular stimulus characteristics (Luck and Hillyard, 1994), or novelty/saliency detection (Riis et al., 2009; Daffner et al., 2015). Because the infrequent stimuli were more complex, these stimuli may elicit larger anterior P2/selection positivity. The anterior P2 is larger in older groups (Daffner et al., 2000, 2015; Czigler and Balázs, 2005; Alperin et al., 2013). Accordingly, in the elderly we expected increased anterior positivity. Furthermore, due to the more efficient inhibitory activity, in Experiment 1 we hypothesized that the infrequent – frequent ERP difference will be less pronounced in the younger group. Following the positive component, infrequent stimuli may elicit a frontal negativity, the N2b component. Irrelevant novel visual stimuli also elicited N2b in an older group (Czigler et al., 2006), with longer latency (Cid-Fernández et al., 2014). The N2b amplitude is usually smaller in older adults (Czigler and Balázs, 2005; Riis et al., 2009; Daffner et al., 2015). Accordingly, we expected the emergence of N2b, and a larger N2b in the younger group. To anticipate the results of the study, the results of Experiment 1 did not correspond to these expectations, and Experiments 1a and 2 replicated the findings of Experiment 1.

To sum up, this study targeted two types of inhibition – task-irrelevant distractor inhibition and response inhibition – by using both behavioral and ERP measures. The tasks were a choice RT (Experiment 1 and Experiment 1a) and simple RT (Experiment 2, to reduce task demand) with infrequent Nogo trials, and we used schematic faces (Experiment 1 and Experiment 2) and threatening objects (Experiment 1a) as salient distractors. Disproportionate delay in RT and increase in error rate were assumed to emerge for less effective inhibition. Regarding ERPs, more effective inhibition of the distractors was hypothesized to result in smaller and earlier posterior N1/N170 and anterior P2/selection positivity, and larger posterior selection negativity and anterior N2b. We also assumed that more effective response inhibition should lead to more correct omissions in the Nogo trials and earlier anterior Nogo P3. Thus, age-related differences in line with these suppositions will reflect different efficiency of distractor inhibition depending on age.

## Experiment 1

The participants performed a choice reaction time (CRT) task that also included infrequent Nogo trials. Participants had to press keys to the change in the color of a disk that otherwise fell into a bin or withhold their response if the color of the disk did not change and the bin disappeared instead.

### Methods^1^

#### Participants

Eighteen older (14 females; mean age 67.44 years, *SD* = 3.76 years) and twenty younger (15 females; mean age 21.9 years, *SD* = 2.21 years) adults participated in Experiment 1. Participants in the older group were recruited from our database and through advertisements on social media. Younger participants were recruited via a school cooperative. Participants were excluded if their performance on the task was lower than the group mean minus two standard deviations, or there were fewer than 40 epochs in either of the analyzed types of trials. After two participants from the younger group were excluded for low performance or for having too few epochs, the data of the 18 older and 18 younger (14 females; mean age 21.56 years, *SD* = 2.06 years) adults were analyzed. All had normal or corrected to normal vision, and no one reported any neurological or psychiatric diseases. Two participants in the younger group and one participant in the older group were left-handed. We excluded dementia-related differences between the age groups by the Full scale Wechsler IQ (measured by the Hungarian version of WAIS-IV, Rózsa et al., 2010). This measure was used in Experiment 1 and Experiment 2. The average IQ of the older and the younger group were 110.67 (*SD* = 13.57) and 115.83 (*SD* = 14.26), respectively. The participants received payment for their participation. The study was approved by the Joint Ethical Review Committee for Research in Psychology (Hungary), and was carried out in accordance with the Declaration of Helsinki. Written informed consent was obtained from all individual participants included in the study.

#### Stimuli and Procedure

**FIGURE 1:**
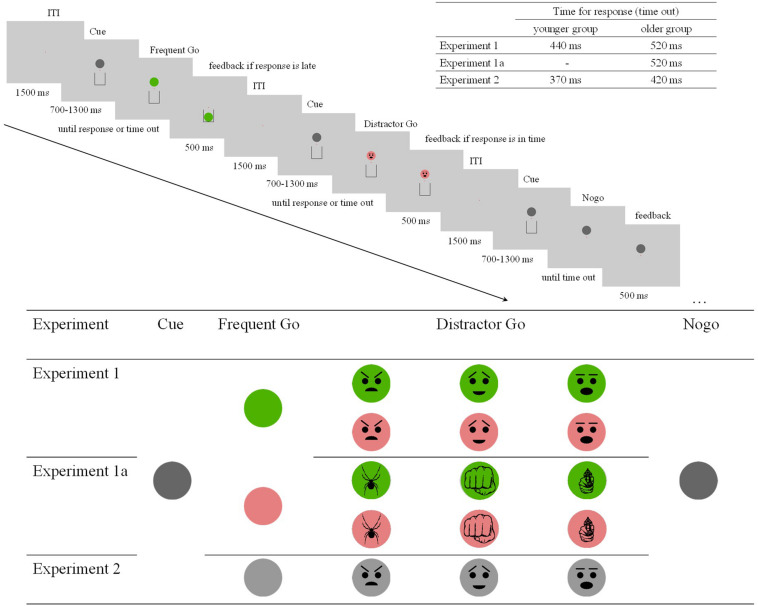
Procedure, time out, and stimuli of the experiments. The stimuli were cue in dark gray, change in color (Experiment 1 and Experiment 1a) or luminosity (Experiment 2) in the case of the Frequent Go stimuli, distractor (superimposed) schematic faces (Experiment 1 and Experiment 2) and threatening objects (Experiment 1a) in the case of the Distractor Go stimuli, and no change in either color or luminosity in the case of the Nogo stimuli.

The experimental stimuli (summarized along with the procedure on Figure 1) were presented on an 18” CRT monitor (LG Flatron F920B, 60 Hz refresh rate, 1280 × 1024 px screen resolution) at a viewing distance of 140 cm.

The experiment included Frequent Go, Distractor Go, and Nogo trials. A trial started with a fixation point, a red dot (RGB 1, 0, 0; 0.11 degrees of visual angle) in the center of the screen on a light grey background (RGB 0.8, 0.8, 0.8; 68.66 cd/m^2^). After 1500 ms a dark gray disk (RGB 0.4, 0.4, 0.4; 11.4 cd/m^2^; 1.76°–1.64°) appeared above the fixation point at 1.23°, and a bin (a 1.86° × 2.34° rectangle with a 0.06°-wide black contour and a missing upper side) appeared underneath at 1.51°. After an average duration of 1000 ms (700–1300 ms in 50 ms steps), for Go trials the color of the disk changed to either green (RGB 0.3, 0.7, 0; 26.9 cd/m^2^) or pink (RGB 0.9, 0.5, 0.5; 26.5 cd/m^2^). At the onset of the color change the participants had to respond as quickly as possible by pressing a key on the left or on the right side depending on the color (the A and L keys on a computer keyboard respectively). The color change - key response correspondence was counterbalanced across participants. The time allowed for a key press (time out) was 440 ms in the younger group and 520 ms in the older group. If the key was not pressed before time out, the disk fell into the bin. The time outs were set as a result of pilot studies and kept the task performance in each group at an average of 80%. In the Go trials the disk was visible for further 500 ms after response, and then the next trial began. In the Frequent Go trials only the color of the disk changed. In the Distractor Go trials along with the change in color a schematic face expressing an emotion (anger, surprise, or happiness) appeared on the disk. We chose schematic faces, because the size of the stimuli was small, and emoticons to increase the salience of the distractors. As ERP research on automatic change detection (vMMN) shows, emotional expression on schematic faces elicits reliable activity even if these stimuli are task-irrelevant (e.g., Kreegipuu et al., 2013). In the Nogo trials there was no change in color, but the bin disappeared. In this case the participants had to withhold their response. Task performance was defined as the percentage of correct responses before time out for the Go trials and correct omissions for the Nogo trials.

The trials were presented in short sequences. The probability of a Frequent Go trial in each sequence was 0.8, the probability of a Distractor Go trial was 0.1, and the probability of a Nogo trial was 0.1. A sequence consisted of 60 trials, thus, there were 48 Frequent Go, 6 Distractor Go, and 6 Nogo trials in a sequence. The color changed to green on half of the Frequent Go and Distractor Go trials and to pink on the remaining half. The Distractor Go and Nogo trials were randomized and randomly inserted in the sequence with at least two and no more than six Frequent Go trials in-between. The number of sequences in the experiment was 18 for a total of 1080 trials (864 Frequent Go, 108 Distractor Go, 108 Nogo trials overall). There were two additional practice sequences at the beginning of the experiment. The participants saw their performance and mean RT at the end of each sequence. The length of each sequence was approximately 2.5 min, and the duration of the experiment was approximately 1 h.

The experiment was realized using Cogent 2000 within MATLAB (MathWorks Inc., 2015) developed by the Cogent 2000 team (Wellcome Department of Imaging Neuroscience, Cogent^2^).

#### EEG Registration

Brain electric activity was recorded (bandwidth: DC-70 Hz; sampling rate 500 Hz) using a BrainAmp DC amplifier system (Brain Products GmbH, Munich, Germany) with Ag/AgCl electrodes placed at 27 locations (F7, F3, FZ, F4, F8, FC3, FC4, T7, C3, CZ, C4, T8, CP5, CP6, P7, P3, PZ, P4, P8, PO7, PO3, POZ, PO4, PO8, O1, OZ, O2) according to the extended 10–20 system by using an elastic electrode cap (EasyCap, Brain Products GmbH). The reference electrode was on the nose tip, and the data were offline re-referenced to the average activity. Eye movements were recorded with four electrodes placed around the eyes. Horizontal EOG was recorded with a bipolar configuration between electrodes positioned lateral to the outer canthi of the eyes (one electrode on each side). Vertical eye movement was monitored with a bipolar montage between two electrodes, one placed below the left eye and one at the AF7 location. The impedance of all electrodes was kept below 10 kΩ. The EEG signal was band-pass-filtered offline with a non-causal Kaiser-windowed FIR filter (lowpass filter parameters: cutoff frequency of 30 Hz, beta of 12.2653, a transition band of 10 Hz; highpass filter parameters: cutoff frequency of 0.1 Hz, beta of 5.6533, a transition band of 0.2 Hz). Because of a constant four-frame delay between the recording of the trigger and the appearance of the stimulus on the screen (measured with a photosensor), epochs were extracted after correcting with 68 ms for the delay. The extracted epochs were with a duration of 800 ms, including a 100 ms pre-stimulus interval, and were stimulus-locked to the color change (Go trials) or the disappearance of the bin (Nogo trials). The mean voltage during the 100 ms pre-stimulus interval served as the baseline for amplitude measurements, and, to remove eye blinks, epochs with an amplitude change exceeding 100 μV on any channel were excluded from averaging.

#### Data Analysis

The trials were separated into Go and Nogo trials for analysis. The Go trials were further divided into Frequent Go and Distractor Go trials. The Frequent Go trials included only the trials before a Distractor Go or a Nogo trial. All responses with RT under 150 ms were removed from further analysis to exclude responses that may have begun before the stimulus change. The RTs were also corrected for the 68 ms delay. Only trials for which the participant gave the correct response were included in the RT and in the ERP analyses.

##### Behavioral data analysis

Overall task performance was compared to confirm that the task difficulty was similar for the two groups as intended.

Performance defined as error rate and mean RT were computed for the Go trials for each participant in the two groups. There were two types of error: misses and incorrect key presses. Both were calculated as a percentage of all Frequent or Distractor Go trials. Mixed analyses of variance (ANOVAs) were calculated with within-subject factor *Trial Type* (Frequent Go, Distractor Go) and between-subject factor *Age* (younger, older). We were interested in whether there was an interaction between age group and Go trial type for either measure which would have indicated a difference in the effect of distraction depending on age. In the case of Nogo trials the percentage of correct omissions of all Nogo trials was calculated and compared between groups to assess response inhibition.

##### EEG data analysis

This section describes both the *a priori* and *post hoc* decisions of the analysis.

###### Regions of interest (ROIs)

Based on the scalp distributions and to reduce the number of comparisons, we formed six ROIs: frontal ROI (F3, Fz, F4), central ROI (C3, Cz, C4), parietal ROI (P3, Pz, P4), occipital ROI (O1, Oz, O2), left parieto-occipital ROI (PO7, P7), and right parieto-occipital ROI (PO8, P8). For the Go trials, the analyses focused on the occipital, left parieto-occipital, and right parieto-occipital ROIs for stimulus change detection (the range of N1/N170), and the frontal and central ROIs for attention-related components (the range of P2 and N2b). For Nogo trials, the analyses were conducted on the ERPs measured at the central and parietal ROIs for the Nogo N2 and the Nogo P3.

###### Go trials

To reduce the number of factors in the comparison between the Frequent Go and Distractor Go trials (Luck and Gaspelin, 2017), Distractor Go *minus* Frequent Go difference potentials were calculated. One-sample *t*-tests were run on the Distractor Go *minus* Frequent Go difference potentials within each group to identify significant deviations from zero in time windows that were defined based on the grand averages for each trial type (because of age-related differences, the time windows could be different in the two groups) (see Table 3 in Results for the time windows). Only significant deviations for at least 11 consecutive data points (20 ms) were considered to indicate a difference between the Distractor Go and the Frequent Go stimuli (Guthrie and Buchwald, 1991).

Peak latencies were measured to assess age-related delays in the processing of the stimuli. For each participant the peak latency was defined as the latency at which the minimum or maximum amplitude value was found within the time window of the investigated component. Mean amplitude values were calculated as the average of the ±10 ms range (a 20 ms duration) around the largest peak in the grand average for each of the time windows.

We added one additional measure of amplitude *post hoc*. A large frontocentral anterior positivity observed in the older group raised the possibility that N2b emerged in Distractor Go trials, but did not reach negative values. Our original approach of comparing mean amplitudes was not appropriate in this case. Instead, peak-to-peak amplitudes were calculated as the difference between the amplitudes at the N2b (younger group) or the negative deflection (older group) and the preceding anterior positivity^3^.

The labels N1/N170 and P2 correspond to our *a priori* view about the expected ERP components. However, as the results suggested, we changed the terminology to ‘anterior positivity’ (anterior counterpart of the posterior negativity and possible P2) and ‘posterior negativity’ (N1/N170 and putative attention-related posterior activity: selection negativity).

###### Nogo trials

For Nogo trials we analyzed the ERPs to the Nogo stimuli. Time windows, peak latencies, and mean amplitudes were defined similarly to those for Go trials.

###### Statistical analysis

Mixed ANOVAs were calculated for peak latencies, mean amplitudes, and peak-to-peak amplitudes with ROI (*Laterality* or *Anteriority*, depending on the investigated component) as a within-subject factor and *Age* (younger, older) as a between-subject factor. The ANOVAs were conducted only on the difference potentials for the Go trials and on the ERPs for the Nogo trials. For all statistical analyses Greenhouse–Geisser corrections were used when necessary. For *post hoc* comparison the Tukey HSD correction was applied. In addition, Bayesian analysis was conducted to evaluate the strength of the evidence for either the null or the alternative hypothesis (Quintana and Williams, 2018; Keysers et al., 2020). The default prior distributions for ANOVA in JASP were used. Specifically, fixed effects had an *r* scale of 0.5, random effects had an *r* scale of 1, and covariates had an *r* scale of 0.354 (where covariates were included in analyses). We also used the default prior option for the *t*-tests, a Cauchy distribution with spread *r* set to 0.707. All tests were two-tailed. Excluding a few exceptions, only results that reached significance (*p* < 0.05) or for which there was moderate to strong evidence for the null hypothesis were reported (BF_10_/BF_incl_ > 3 or < 0.333). A summary of all statistical analysis is available in Supplementary Data Sheet 1.^4^

##### sLORETA analysis

We applied a distributed source localization technique to locate and compare the cortical sources for potential differences between the groups. This analysis was decided upon *post hoc*, it was entirely exploratory and was conducted because of the frontal positivity and posterior negativity differences between the Distractor Go and the Frequent Go stimuli in the older group found in the ERP analysis (see Results). It was intended to supplement the ERP results.

The source signal of the average ERP time series was reconstructed on the cortical surface by applying the sLORETA inverse solution (Pascual-Marqui, 2002). The sLORETA gives a solution for the EEG inverse problem by applying a weighted minimum norm estimation with spatial smoothing and standardization of the current density map. The forward model was generated on a realistic BEM head model (Gramfort et al., 2010) by applying a template MRI (ICBM152; 1 mm^3^ voxel resolution) with template electrode positions. The reconstructed dipoles (pA/m) were determined for every 15,002 sources in three orthogonal directions (unconstrained solution).

For each subject the sources for the Frequent Go and Distractor Go ERPs were estimated, their difference (Distractor Go *minus* Frequent Go) computed, then normalized to baseline and flattened. The differences were averaged in 6 equal, 20-ms-long intervals from 90 to 210 ms, and then compared to 0 with parametric one-sample χ^2^ -tests for unconstrained sources for each group separately. This analysis shows when and where there are differences between the two conditions within each age group. The differences were reported as significant, if at least 20 voxels exceeded the Bonferroni corrected alpha level (α = 0.05). For the comparison between the groups non-parametric two-sample independent *t*-tests were conducted for the same time intervals on the Distractor Go *minus* Frequent Go differences, and FDR correction (α = 0.01) was applied (Tadel et al., 2019). Brain regions for the corresponding significant activations were identified based on the parcelation scheme introduced by Klein and Tourville (2012).

The EEG data were processed with MATLAB R2015a (MathWorks Inc., 2015) and the EEGLAB 13.6.5b toolbox (Delorme and Makeig, 2004). The sLORETA analysis was performed with Brainstorm (Tadel et al., 2011), which is documented and freely available for download online under the GNU general public license^5^. Group analysis was conducted according to the Group analysis processing pipeline described in Tadel et al. (2019). Statistical analyses were performed with Statistica 13 (Dell Inc., 2016). Bayesian analysis was performed with JASP 0.13.1 (JASP Team, 2020).

### Results

#### Summaries

For all experiments Table 1 presents the descriptive statistics of the behavioral results, Table 2 summarizes the number of epochs for each type of trial, Table 3 summarizes the time windows for the difference potentials (Go trials) and ERP (Nogo trials) analysis, Table 4 shows the averaged peak latencies and mean amplitudes, and the averaged peak-to-peak amplitudes can be found in Table 5.

**TABLE 1:** Percentage of correct omissions in the Nogo trials and misses, incorrect key presses rates, and reaction times (RTs) for the Frequent Go and Distractor Go trials in the older and younger groups for each experiment (mean and *SEM*).

Experiment	Group	Correct omissions (%)	Misses (%)		Incorrect key presses (%)		Reaction time (ms)	
		Nogo	Frequent Go	Distractor Go	Frequent Go	Distractor Go	Frequent Go	Distractor Go
Experiment 1	Younger	99.43% (0.23%)	0.47% (0.23%)	0.47% (0.29%)	6.17% (0.57%)	7.16% (1.03%)	306.27 (4.03)	326.72 (3.45)
	Older	99.85% (0.08%)	0.23% (0.09%)	0.62% (0.28%)	4.42% (0.83%)	5.25% (0.81%)	378.02 (5.67)	396.18 (6.12)
Experiment 1a	Older	99.81% (0.09%)	0.42% (0.15%)	0.65% (0.29%)	4.03% (0.82%)	6.76% (1.12%)	370.13 (4.05)	390.95 (3.88)
Experiment 2	Younger	75.64% (3.58%)	2.09% (0.45%)	1.61% (0.3%)	–	–	236.79 (2.57)	235.96 (3.21)
	Older	87.96% (2.5%)	1.68% (0.33%)	0.99% (0.3%)	–	–	275.01 (3.8)	270.65 (4.77)

**TABLE 2:** Average number of epochs for each participant per trial type for each experiment (mean and range).

Experiment	Group	Frequent Go	Distractor Go	Nogo
Experiment 1	Younger	190.06(172−206)	96.5(84−104)	96.06(55−108)
	Older	193.11(145−212)	97.39(81−106)	101.39(80−108)
Experiment 1a	Older	189.4(122−211)	94.5(62−105)	97.6(50−108)
Experiment 2	Younger	177.38(97−213)	91(56−108)	68.13(42−95)
	Older	167.06(60−214)	86.17(48−108)	82.44(43−106)

**TABLE 3:** Time windows for the analyses of the difference potentials (Go trials) and ERPs (Nogo trials) components per group and per experiment (*post stimulus* range in ms).

Experiment	Group	Posterior negativity			Anterior positivity		N2b		Nogo P2	Nogo P3	
		Occipital	Left parieto-occipital	Right parieto-occipital	Frontal	Central	Frontal	Central	Parietal	Central	Parietal
Experiment 1	Younger	100–200 ms			100–200 ms		150–300 ms		100–250 ms	200–700 ms	
	Older	100–250 ms			100–250 ms		150–300 ms		100–200 ms	200–700 ms	
Experiment 1a	Older	100–250 ms			100–250 ms		–	–	–	–	–
Experiment 2	Younger	100–200 ms			100–250 ms	100–200 ms	200–400 ms		100–300 ms	200–700 ms	
	Older	100–250 ms			100–250 ms		200–400 ms		100–200 ms	200–700 ms	

**TABLE 4:** Average peak latencies and mean amplitudes for the Distractor Go *minus* Frequent Go difference potentials and the Nogo ERPs (mean and *SEM*).

	Experiment	Group	Posterior negativity			Anterior positivity		N2b		Nogo N2	Nogo P3	
			Occipital	Left parieto-occipital	Right parieto-occipital	Frontal	Central	Frontal	Central	Parietal	Central	Parietal
Peak latency	Experiment 1	Younger	140.2 (6.4)	140.8 (5.7)	135.4 (3.3)	155.9 (6.8)	138.8 (3.9)	237.9 (15.2)	220.1 (9.5)	144.7 (3.4)	383.2 (24.4)	501.7 (27.8)
		Older	158.8 (3.6)	154.7 (3.3)	158.6 (2.4)	164.4 (4.6)	156.8 (2.6)	252 (5.4)	250.1 (6.5)	151.4 (3.2)	585.4 (29.6)	609.9 (28.5)
	Experiment 1a	Older	163.6 (6.9)	167.6 (5.0)	168.1 (5.3)	173.6 (6.2)	169.7 (5.8)	–	–	–	–	–
	Experiment 2	Younger	159.1 (7.9)	149.1 (5.1)	150.8 (5.5)	175.5 (7.4)	161.6 (6.5)	308.5 (12.6)	285.4 (10.1)	172.6 (8.3)	353 (14.4)	421 (20.4)
		Older	161.3 (5.4)	164.9 (3.2)	171.7 (4.8)	172.8 (4.3)	167.3 (3.3)	277.6 (12.0)	285.9 (10.2)	156.3 (4.8)	452.7 (24.3)	567.8 (21.9)
Mean amplitude	Experiment 1	Younger	−0.47(0.43)	−2.88(0.72)	−3.78(1.01)	0.8 (0.48)	1.51 (0.4)	–	–	−2.26(0.39)	7.53 (0.85)	3.68 (0.39)
		Older	−4.37(0.61)	−5.85(0.77)	−7.8(0.86)	4.17 (0.48)	4.07 (0.4)	–	–	−1.54(0.25)	5.03 (0.43)	2.11 (0.44)
	Experiment 1a	Older	−3.4(0.76)	−3.42(0.58)	−3.73(0.8)	2.15 (0.54)	2.59 (0.42)	–	–	–	–	–
	Experiment 2	Younger	0.29 (0.56)	−2.44(0.71)	−2.71(1.09)	0.43 (0.46)	0.91 (0.47)	–	–	−1.66(0.35)	8.75 (0.78)	5.56 (0.7)
		Older	−4.02(0.68)	−6.94(0.82)	−6.49(0.8)	4.02 (0.6)	4.34 (0.46)	–	–	−1.21(0.3)	6.38 (0.57)	3.24 (0.56)

**TABLE 5:** Average peak-to-peak amplitudes for the Distractor Go *minus* Frequent Go difference potentials (mean and *SEM*).

Experiment	Group	N2b	
		Frontal	Central
Experiment 1	Younger	3.82 (0.33)	4.37 (0.43)
	Older	5.37 (0.57)	5.05 (0.38)
Experiment 1a	Older	–	–
Experiment 2	Younger	6.52 (0.76)	6.08 (0.77)
	Older	5.56 (0.68)	5.64 (0.5)

**FIGURE 2:**
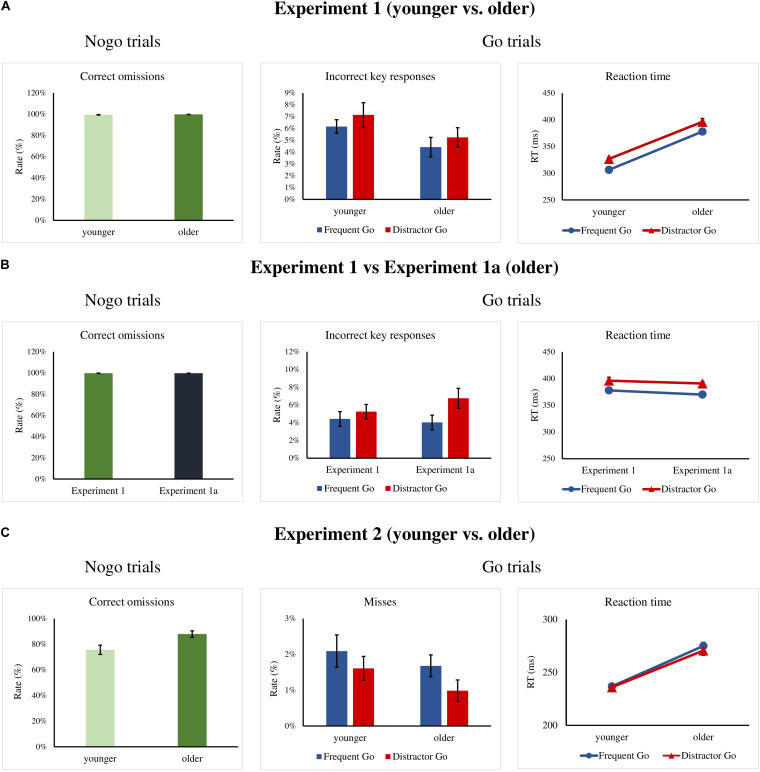
**(A)** Experiment 1: Percentage of correct omissions in the Nogo trials and incorrect key presses rates and reaction times (RTs) for the Frequent Go and Distractor Go trials in the older and younger groups. **(B)** Experiment 1a: Percentage of correct omissions in the Nogo trials and incorrect key presses rates and RTs for the Frequent Go and Distractor Go trials in the Experiment 1 and 1a for the older groups. **(C)** Experiment 2: Percentage of correct omissions in the Nogo trials and misses and RTs for the Frequent Go and Distractor Go trials in the older and younger groups. Error bars indicate *S.E.M*.

**FIGURE 3:**
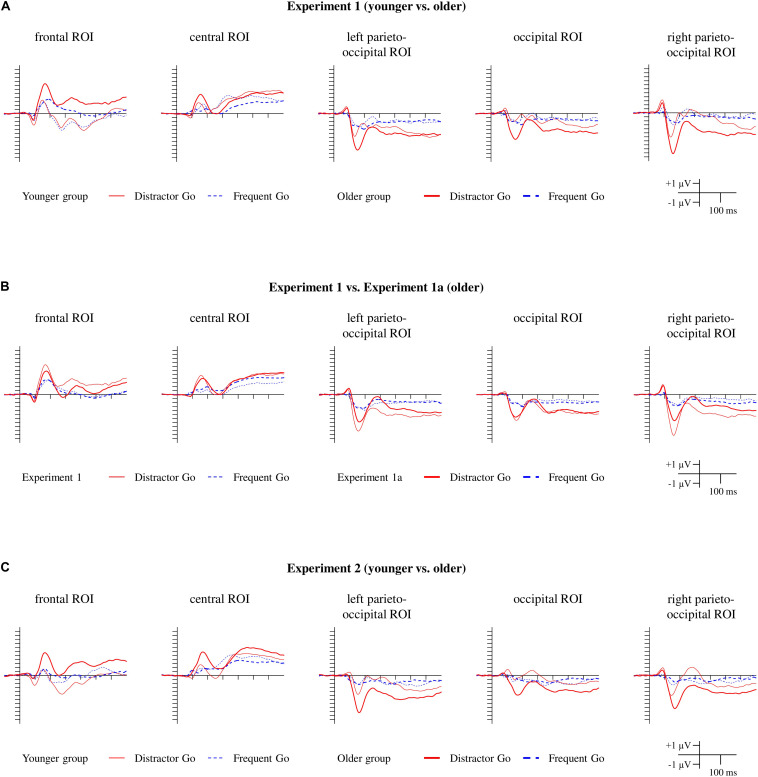
ERPs for the Distractor Go and Frequent Go trials for Experiment 1 **(A)**, Experiment 1a **(B)**, and Experiment 2 **(C)**. The red lines indicate ERPs to the Distractor Go stimuli, and the blue dashed lines indicate ERPs to the Frequent Go stimuli. For Experiment 1 and Experiment 2 the thin lines indicate the younger group, and the thick lines indicate the older group. For Experiment 1a, the ERPs measured in the older group are indicated by the thick lines, and the ERPs from the older group in Experiment 1 are depicted with thin lines for comparison.

**FIGURE 4:**
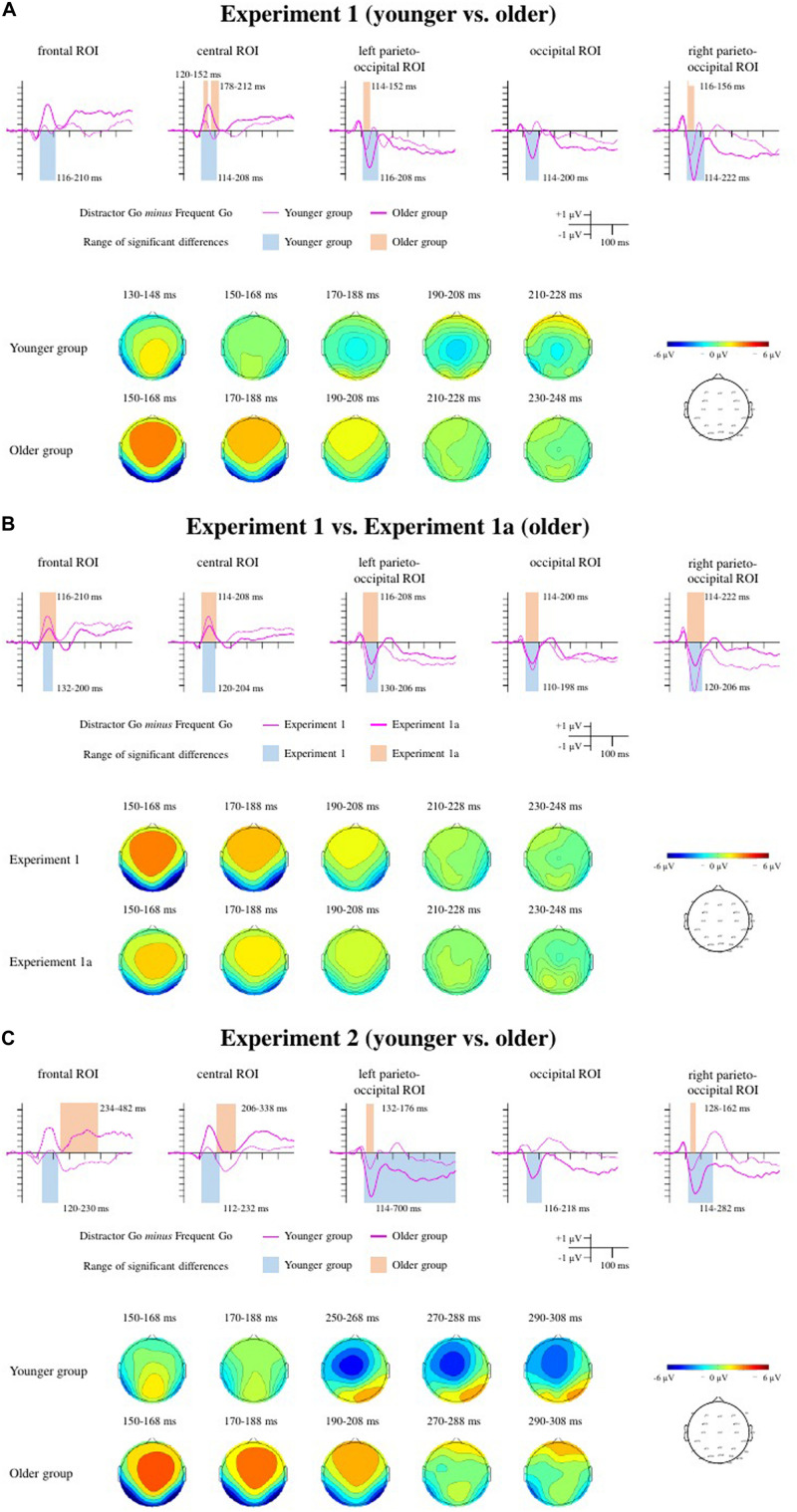
Difference potentials, range of significant differences, and scalp distributions for the Distractor Go *minus* Frequent Go difference potentials for Experiment 1 **(A)**, Experiment 1a **(B)**, and Experiment 2 **(C)**. For Experiment 1 and Experiment 2 the thin lines indicate the younger group, and the thick lines indicate the older group. For Experiment 1a, the difference potentials measured in the older group are indicated by the thick lines, and those from the older group in Experiment 1 are depicted with thin lines for comparison. The ranges of significant differences are depicted with color rectangles: In Experiment 1 and Experiment 2 orange indicates the younger group and blue indicates the older group. In Experiment 1a blue indicates the ranges of significant differences in the older group and orange for the older group from Experiment 1.

**FIGURE 5:**
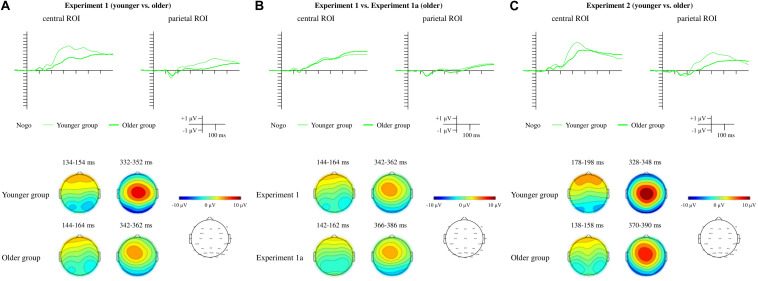
ERPs and scalp distributions for the Nogo trials for Experiment 1 **(A)**, Experiment 1a **(B)**, and Experiment 2 **(C)**. For Experiment 1 and Experiment 2 the thin lines indicate the younger group, and the thick lines indicate the older group. For Experiment 1a, the difference potentials measured in the older group are indicated by the thick lines, and those from the older group in Experiment 1 are depicted with thin lines for comparison.

Figure 2 depicts the behavioral results for each experiment. The ERPs to the Frequent Go and Distractor Go trials (included for visual inspection only) are depicted on Figure 3. The Distractor Go *minus* Frequent Go difference potentials along with the ranges of significant differences from the point-by-point *t*-tests as well as scalp distributions can be found on Figure 4. The ERPs and scalp distributions of the Nogo trials are shown on Figure 5.

**FIGURE 6:**
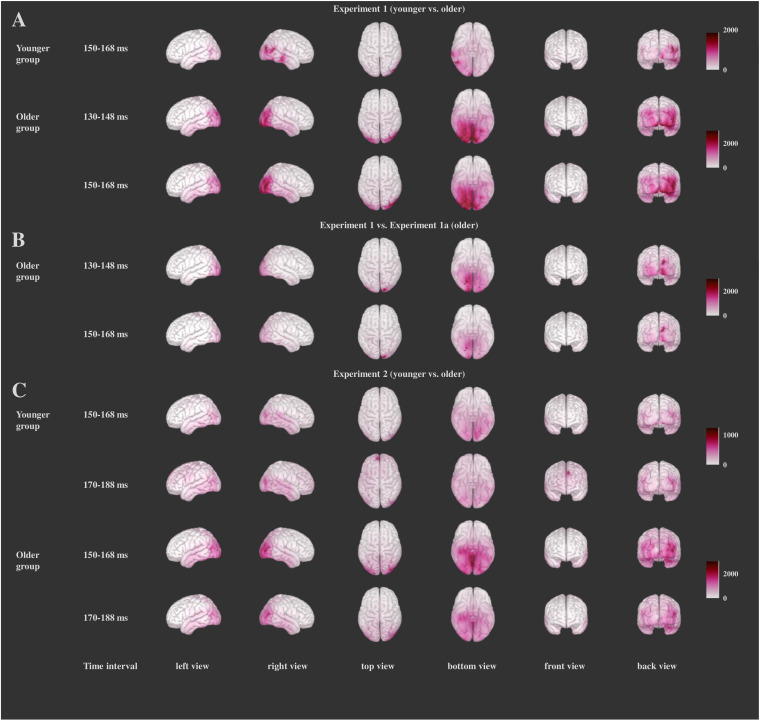
Significant differences between the Distractor Go and Frequent Go conditions within the groups for each experiment (χ^2^-test values of the differences) for Experiment 1 **(A)**, Experiment 1a **(B)**, and Experiment 2 **(C)**. Darker color indicates larger χ^2^-test values. Only the time intervals for which the between-group comparisons yielded significant results are depicted.

**FIGURE 7:**
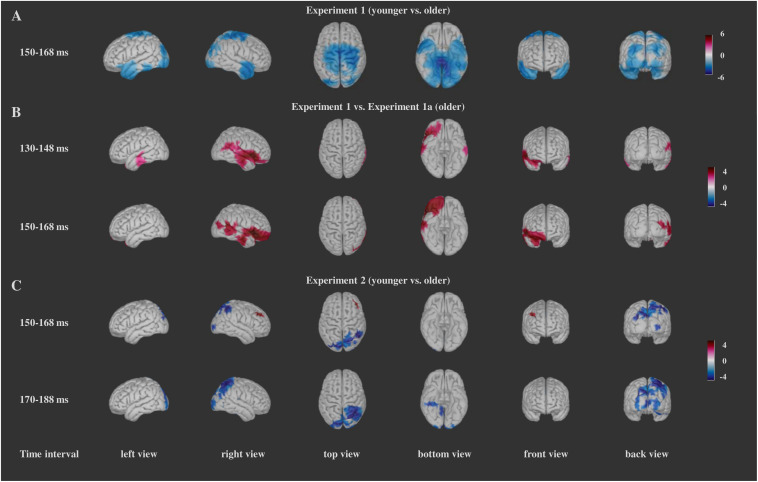
Significant differences for the comparisons between the groups (*t*-values of the differences) for Experiment 1 **(A)**, Experiment 1a **(B)**, and Experiment 2 **(C)**. For Experiment 1 and Experiment 2 blue color indicates larger activity in the older group, and red color indicates larger activity in the younger group. For the comparison between Experiment 1a and Experiment 1, red color indicates larger activity in the older group from Experiment 1, and blue color indicates larger activity in the older group from Experiment 1a. Darker color indicates larger *t*-values.

The results of the source localization for the comparisons between the Frequent Go and Distractor Go trials are presented on Figure 6, and those for the comparisons between the groups are depicted on Figure 7. A list of the brain regions for which the largest differences were found between the Frequent Go and the Distractor Go trials and a list of the brain regions with significant differences between the groups can be found in the Supplementary Material (Supplementary Tables 1, 2 respectfully). In all cases only the time intervals for which the between-group comparison yielded significant differences are shown.

#### Behavioral Results

A total of 10 trials in the younger group and only 1 trial in the older group were removed for RTs that were too fast. Task performance was 81.29% (*SD* = 6.45%) in the younger group and 79.19% (*SD* = 9.36%) in the older group. There was no difference between the groups, *t*(34) = −0.76, *p* = 0.453, *Cohen’s d* = 0.261, BF_10_ = 0.403, with median posterior δ = −0.191. 95% credible interval = [−0.793, 0.375]. Thus, the task had a similar difficulty level in the two age groups.

For Go trials, the rate of misses was below 1%, so these were excluded from further analysis. We did not find significant main effects for the error rates (incorrect key presses, Figure 2A and Table 1), and there was moderate evidence for a null effect for the interaction, *F*(1,34) = 0.033s, *p* = 0.857, η_p_^2^ < 0.001, BF_incl_ = 0.288.

Mean RTs for the Frequent Go and Distractor Go trials showed a main effect of *Trial Type*, *F*(1,34) = 162.32, *p* < 0.001, η_p_^2^ = 0.827, BF_incl_ = 9.268 × 10^10^, with participants being faster for Frequent Go than for Distractor Go trials. The main effect of *Age* was also significant, *F*(1,34) = 107.07, *p* < 0.001, η_p_^2^ = 0.759, BF_incl_ = 6.552 × 10^8^. The older participants were overall slower than the younger participants (Figure 2A and Table 1). The interaction was not significant.

Performance for Nogo trials was at ceiling in both age groups, thus these data were not further investigated (Figure 2A and Table 1).

#### Event-Related Potentials

##### Go trials

First, we visually inspected the ERPs of the Frequent Go and Distractor Go trials (Figure 3A). At the occipital, left parieto-occipital and right parieto-occipital ROIs a posterior negativity was observed in both groups that was larger for the Distractor Go compared to Frequent Go trials in the older group, but not in the younger group.

At the frontal ROI an anterior positivity and the N2b component were observed in the younger group, but only an anterior positivity was seen in the older group. The Distractor Go trials seemed to elicit a much larger anterior positivity than Frequent Go trials in the older group. At the central ROI only the anterior positivity emerged for both groups in the Go trials, but the difference potential indicated a possible emergence of N2b in the younger group.

Next, we conducted statistical analyses on the Distractor Go *minus* Frequent Go difference potentials.

###### Posterior negativity

In the younger group the consecutive one-sample *t*-tests showed significant differences at the left parieto-occipital and right parieto-occipital ROI. In the older group, there were significant differences at all three ROIs (see Table 3 for the time windows and Figure 4A for the ranges). A mixed ANOVA with within-subject factor *Laterality* (occipital, left parieto-occipital, right parieto-occipital) and between-subject factor *Age* (younger, older) on peak latencies (Table 4) showed only a significant main effect of *Age*, *F*(1,34) = 16.601, *p* < 0.001, η_p_^2^ = 0.328, BF_incl_ = 94.186. The peak latency was longer in the older group. There was also moderate evidence for the lack of interaction, *F*(1,34) = 0.822, *p* = 0.444, η_p_^2^ = 0.024, BF_incl_ = 0.259. Regarding mean amplitudes (Table 4), we found both main effects of *Age*, *F*(1,34) = 17.448, *p* < 0.001, η_p_^2^ = 0.339, BF_incl_ = 127.674 and *Laterality*, *F*(2,68) = 19.715, *p* < 0.001, ε = 0.734, η_p_^2^ = 0.367, BF_incl_ = 91769.9. The mean amplitude was larger in the older group compared to the younger group, largest at the right parieto-occipital ROI compared to the occipital (*p* < 0.001) and left parieto-occipital ROIs (*p* = 0.027), and larger at the left parieto-occipital ROI compared to the occipital ROI (*p* = 0.002). The evidence for the interaction was moderate in favor of the null hypothesis, *F*(1,34) = 0.57, *p* = 0.518, η_p_^2^ = 0.017, BF_incl_ = 0.216.

###### Anterior positivity

The consecutive one-sample *t*-tests on the Distractor Go *minus* Frequent Go difference potentials showed a significant difference only at the central ROI in the younger group. In the older group there were significant differences at both the frontal and the central ROIs (see Table 3 for the time windows and Figure 4A for the ranges). Peak latencies (Table 4) were compared between groups with a mixed ANOVA with a within-subject factor of *Anteriority* (frontal, central) and between-subject factor of *Age* (younger, older). There was a main effect of *Anteriority*, *F*(1,34) = 20.861, *p* < 0.001, η_p_^2^ = 0.38, BF_incl_ = 51.52, with an overall longer latency at the frontal ROI (*p* < 0.001). There was a significant but not conclusive interaction between *Anteriority* and *Age*, *F*(1,34) = 10.572, *p* = 0.003, η_p_^2^ = 0.237, BF_incl_ = 2.224. *Post hoc* comparisons showed that the latency was longer at the frontal ROI in the younger (*p* < 0.001), but not in the older group (*p* = 0.789). The comparison of mean amplitudes (Table 4) showed a main effect of *Age*, *F*(1,34) = 24.045, *p* < 0.001, η_p_^2^ = 0.414, BF_incl_ = 462.122, a main effect of *Anteriority* which was not conclusive, *F*(1,34) = 4.318, *p* = 0.045, η_p_^2^ = 0.113, BF_incl_ = 0.996, and a significant interaction, *F*(1,34) = 7.468, *p* = 0.01, η_p_^2^ = 0.18, BF_incl_ = 4.687. The mean amplitude was larger in the older group (*p* < 0.001 for both ROI compared to the younger group) and larger at the central ROI, but only in the younger group (*p* < 0.001).

###### N2b

The one-sample *t*-tests on the Distractor Go *minus* Frequent Go difference potentials indicated a significant difference at the central ROI in the younger group. In the older group, the significant differences belonged either to the anterior positivity at both ROIs, or to a long-lasting positivity (frontal ROI) and the P3 range (central ROI) (see Table 3 for the time windows and Figure 4A for the ranges). Nevertheless, peak latencies (Table 4) were measured for the minimum reached within the time windows and compared with a repeated measures ANOVA. The main effects and the interaction were not significant. The results of the peak-to-peak amplitude comparison were likewise not significant (Table 5), although there was moderate evidence against the main effect of *Anteriority*, *F*(1,34) = 0.18, *p* = 0.679, η_p_^2^ = 0.005, BF_incl_ = 0.253.

### Nogo Trials

For the Nogo trials (Figure 5A) a Nogo P3 was visible at the central and parietal ROIs. A Nogo P2 was observed only at the parietal ROI.

#### Nogo N2

There was no difference between the groups for peak latencies or mean amplitudes for the Nogo N2 at the parietal ROI (Table 4).

#### Nogo P3

Peak latencies and mean amplitudes (Table 4) of the Nogo P3 were compared between groups with a mixed ANOVA with a within-subject factor *Anteriority* (central, parietal) and a between-subject factor *Age* (younger, older). The main effects of *Age* and *Anteriority* were significant in the case of peak latencies, *F*(1,34) = 22.823, *p* < 0.001, η_p_^2^ = 0.401, BF_incl_ = 528.862 and *F*(1,34) = 10.818, *p* = 0.002, η_p_^2^ = 0.241, BF_incl_ = 12.868, respectively. The interaction was also significant but inconclusive, *F*(1,34) = 4.682, *p* = 0.038, η_p_^2^ = 0.121, BF_incl_ = 1.864. The *post hoc* comparisons showed that P3 peaked earliest in the younger group at the central ROI (*p* = 0.003 compared to the parietal ROI in the younger and both *p*s < 0.001 compared to both ROI in the older group). The latency of P3 at the parietal location in the younger group also differed from that in the older group (*p* = 0.037). Finally, the latency of P3 in the older group did not differ by *Anteriority* (*p* = 0.856). In the case of mean amplitudes, there was a significant main effect of *Age*, *F*(1,34) = 9.103, *p* = 0.005, η_p_^2^ = 0.211, BF_incl_ = 8.988, and of *Anteriority*, *F*(1,34) = 65.772, *p* < 0.001, η_p_^2^ = 0.659, BF_incl_ = 2.912 × 10^7^. The Nogo P3 was larger in the younger group and larger at the central ROI. The interaction was not significant.

#### Source Localization

Our analysis showed significant differences for the whole cortex (above 99% in each of the time intervals). This was one of the possible outcomes as the parametric one-sample χ^2^-test is a very sensitive test (Tadel et al., 2016). To isolate the areas with greatest difference, for each time interval we determined the voxels for which the χ^2^-test values were above the mean plus two standard deviations as well as the regions that had at least 20 voxels with such values. For this time interval (150–168 ms, see below) the Distractor Go *minus* Frequent Go activity is reflected by a right-sided broad posterior network the right fusiform gyrus, right inferior parietal, right lateral occipital, right lingual, right middle temporal and right pericalcarine cortex in both groups, in the right inferior temporal cortex for the younger group, and in the left lateral occipital, left lingual, right superior parietal cortex, right cuneus and right precuneus in the older group (Figure 6A and Supplementary Table 1).

The comparison between the two age groups showed widespread bilateral differences in the activity in the occipital, temporal, parietal, central, and posterior cingulate regions as well as smaller differences in the left and right caudal middle frontal, the left and right entorhinal, right insula and right lateral orbitofrontal in the 150–168 ms time interval. The activation was larger in the older group (Figure 7A and Supplementary Table 2).

### Discussion

We adjusted the RT time limit to equate the general error rate, thus the longer RT corresponds to the general slowing tendency in the elderly (e.g., Salthouse, 1996). With the increased time out the older group performed fairly well in the task. Importantly, we obtained no significant *Trial Type* × *Age* interaction for RT or error rate. In the case of RT, the result is inconclusive. However, when the difference between the Frequent Go and the Distractor Go RTs is calculated for each participant, then the difference between the group averages is only ∼2.3 ms, and likely not of interest The Nogo stimuli elicited hardly any erroneous reactions.

We restrict the discussion of Experiment 1 and 1a to the ERP differences between the ERPs to the Frequent Go and Distractor Go (infrequent) stimuli. Other age-related effects and effects of infrequent and frequent stimuli will be provided in the General discussion.

In the ERPs to both Go stimuli in both groups there was an anterior positivity within the 100–200 ms range, and a posterior negativity within the same range. While the posterior negativity was observed even to the Frequent Go stimuli (see the ERPs on Figure 3), the exogenous N1 and/or the face-related N170 is a part of this negativity. Exogenous anterior positivity in this range is less common to visual stimuli; task-unrelated visual stimuli usually elicit a posterior N1 (e.g., Čeponienė et al., 2008). In the Čeponienė et al. (2008) study N1 had larger amplitudes in younger participants at both anterior and posterior locations compared to the older participants. Therefore, it is improbable that the anterior positivity in the present study was due to exogenous activity, and the increased activity to the Distractor Go stimuli was due to the onset of pattern *per se*. Furthermore, the age-related increase of anterior positivity and posterior negativity is in contrast with the age-related decrease of exogenous activity in this range (Čeponienė et al., 2008) and also with the larger alerting effects on this component in younger participants (Kaufman et al., 2016; Williams et al., 2016). As another possibility, the Distractor Go stimuli contained schematic faces, and the larger components to these stimuli could be due to the involvement of face-specific activity. In fact, Chaby et al. (2003); de Fockert et al. (2009), Gao et al. (2009), and Daniel and Bentin (2012) obtained larger face specific N170 in older participants (but see Pfütze et al., 2002). Furthermore, Jeffreys and Tukmachi (1992) reported a central counterpart of face specific activity. To investigate whether the anterior positivity and posterior negativity is a face-specific effect, in Experiment 1a we introduced non-facial distractors to control for the face specificity of the anterior positivity/posterior negativity.

As the localization results showed, the infrequent distractors elicited increased activity in visual areas dealing with elementary visual features as well as object-related processing. It is possible that areas with specific sensitivity to facial stimuli within the fusiform and lingual gyri contributed to this difference, therefore the possibility of face specificity of distractors deserves investigation. In both age groups activity differences between the effects of infrequent and frequent stimuli concentrated on the right side. As a general finding, older group do not show any asymmetric scalp distribution (Pfütze et al., 2002; Chaby et al., 2003; Gao et al., 2009; Daniel and Bentin, 2012), which is against the face-specificity interpretation of the increased activity to infrequent stimuli.

The posterior ERP activity in the 150–168 ms range was sensitive to both stimulus-related features (exogenous N1/N170) and attentional influences (selection negativity). Besides the increased sensitivity of the posterior structures, in the older group we obtained increased sensitivity in anterior areas, showing that attentional processes play a more important role in this age group. As the behavioral results showed, besides the general slowing in the older group, these participants were fairly effective in performing the task. Therefore we hypothesize that the function of the anterior structures contributed to the compensation of the increased task-irrelevant modality specific activity.

## Experiment 1a

The use of faces as distractors resulted in a large ERP difference between the Frequent and Distractor Go stimuli in Experiment 1 within the 100–200 ms latency range both posteriorly and anteriorly. It is possible that this posterior negative and anterior positive difference potential was due to the onset of a salient distractor. However, event-related activity specific to facial stimulation could also contribute to the enlarged activity. As the sLORETA localization indicated, a wide set of posterior structures contributed to the activity difference, and among these structures the fusiform and lingual gyri have face-specific areas. Furthermore, the much larger effect in the older group may correspond to results showing larger face-specific posterior activity in elderly (e.g., Daniel and Bentin, 2012). In Experiment 1a we used the same method as in Experiment 1, but instead of facial distractors we utilized other objects: line drawings of a spider, a fist, and a pistol, i.e., potentially salient objects (Zsido et al., 2019) (see Figure 1). Another difference was that the stimuli were presented on a 24′′ monitor (ASUS VG245HE, 60 Hz refresh rate, 1920 × 1080 px screen resolution). Because we were interested in the robust anterior positivity and posterior negativity in the older group, Experiment 1a included only older participants. The between-subject factor was relabeled *Experiment* (Experiment 1, Experiment 1a). We restricted the comparison of ERPs and difference potentials between Experiment 1 and Experiment 1a to the 100–250 ms range.

### Methods

#### Participants

Based on the data from Experiment 1 and 2 for the Distractor Go *minus* Frequent Go difference potential and Experiment 1 for the RT difference between the two trial types, we were able to calculate the sample sizes required for obtaining similarly large differences in RT, the anterior positivity amplitude, and the posterior negativity amplitude. The calculation was performed with G^∗^Power 3.1.9.4 (Faul et al., 2007). The results showed that to achieve power 0.99 the largest total sample required was 12 participants (for details, see Supplementary Data Sheet 3).

Twenty-five older adults participated in the experiment (14 females; mean age 66.76 years, *SD* = 4.09 years). Two participants did not complete the session. Two participants were excluded for performance on the task at around 50% which indicates random responding, and one participant was then excluded for low performance. Thus, the data of 20 participants (11 females; mean age 66.45 years, *SD* = 4.2 years) were analyzed. All had normal or corrected to normal vision, and no one reported any neurological or psychiatric diseases. All participants were right-handed. The participants received payment for their participation. We excluded dementia-related cognitive changes by measuring four subtests of the WAIS-IV, representing four major components of intelligence (Similarities, Digit Span, Matrix Reasoning, Coding). The mean total score of the group was 51.52, *SD* = 7.29.

### Results

#### Behavioral Results

Task performance was 82.73% (*SD* = 7.3%) and there was no difference when compared to the performance of the older group in Experiment 1, *t*(36) = 1.27, *p* = 0.212, *Cohen’s d* = 0.422, BF_10_ = 0.593, with median posterior δ = −0.321. 95% credible interval = [−0.931, 0.241]. Thus, the task had a similar level of difficulty for both groups.

The rate of misses for Go trials was below 1%, so these results were not further analyzed. The rates of incorrect key presses (Figure 2B and Table 1) for the two older groups were compared for *Trial Type* (Frequent Go, Distractor Go) as a within-subject factor and *Experiment* (Experiment 1, Experiment 1a) as the between-subject factor. There was a main effect of *Trial Type*, *F*(1,36) = 15.26, *p* < 0.001, η_p_^2^ = 0.298, BF_incl_ = 54.925 with more errors in the case of Distractor Go trials. There was no main effect of *Experiment*, and the interaction was significant but not conclusive, *F*(1, 36) = 4.404, *p* = 0.043, η_p_^2^ = 0.109, BF_incl_ = 1.764. The *post hoc* comparisons showed that there was a difference in the rate of incorrect key presses between Frequent Go and Distractor Go trials only in Experiment 1a (*p* < 0.001).

Mean RTs for Frequent Go and Distractor Go trials (Figure 2B and Table 1) were compared for *Trial Type* (Frequent Go, Distractor Go) as a within-subject factor and *Experiment* (Experiment 1, Experiment 1a) as the between-subject factor. There was a main effect of *Trial Type*, *F*(1,36) = 92.38, *p* < 0.001, η_p_^2^ = 0.72, BF_incl_ = 4.306 × 10^8^, with faster RTs for Frequent Go than for Distractor Go trials. The main effect of *Experiment* and the interaction were not significant.

The rate of correct omissions for Nogo trials was again at ceiling, thus we did not analyze it further (Figure 2B and Table 1).

#### Event-Related Potentials

Experiment 1a was conducted with the goal of determining whether the type of Distractor Go stimuli (i.e., faces vs. threatening stimuli) affects the latency and amplitude of the anterior positivity/posterior negativity facial distractors. Nevertheless, the ERPs clearly show very close overlaps and identical morphology for all trial types in the two experiments (Figures 3B, 4B, 5B).

##### Go trials

###### Posterior negativity

The consecutive one-sample *t*-tests showed significant differences at the occipital, the left parieto-occipital, and the right parieto-occipital ROIs (see Table 3 for the time windows and Figure 4B for the ranges). A mixed ANOVA with a within-subject factor *Laterality* (occipital, left parieto-occipital, right parieto-occipital) and a between-subject factor *Experiment* (Experiment 1, Experiment 1a) was conducted on the peak latencies (Table 4). Neither the main effects nor the interaction were significant. There was moderate evidence for lack of a main effect of *Laterality*, *F*(1,36) = 0.345, *p* = 0.709, η_p_^2^ = 0.01, BF_incl_ = 0.114, and a lack of interaction, *F*(1,36) = 0.915, *p* = 0.405, η_p_^2^ = 0.024, BF_incl_ = 0.273. The same comparison for mean amplitudes (Table 4) yielded a main effect of *Experiment*, *F*(1,36) = 7.85, *p* = 0.008, η_p_^2^ = 0.179, BF_incl_ = 6.339, a main effect of *Laterality*, *F*(2,72) = 7.943, *p* = 0.002, ε = 0.803, η_p_^2^ = 0.181, BF_incl_ = 14.120, and a significant interaction between the two factors, *F*(2,72) = 5.306, *p* = 0.012, ε = 0.803, η_p_^2^ = 0.129, BF_incl_ = 5.714. The *post hoc* comparisons revealed that the posterior negativity at the right parieto-occipital ROI for Experiment 1 was larger compared to the mean amplitude measured at all three ROIs in Experiment 1a (*p* = 0.001 for the occipital, *p* = 0.001 for the left parieto-occipital, and *p* = 0.003 for the right parieto-occipital).

###### Anterior positivity

The consecutive one-sample *t*-tests showed significant differences at both frontal and central ROIs (see Table 3 for the time windows and Figure 4B for the ranges). Peak latencies (Table 4) were compared with a mixed ANOVA with a within-subject factor *Anteriority* (frontal ROI, central ROI) and a between-subject factor *Experiment* (Experiment 1, Experiment 1a). The main effect of *Anteriority* was significant but inconclusive, *F*(1,36) = 5.523, *p* = 0.024, η_p_^2^ = 0.133, BF_incl_ = 2.075, with the peak latency being shorter at the central ROI. The main effect of *Experiment* and the interaction were not significant. The same comparison for peak amplitudes (Table 4) showed a main effect of *Experiment*, *F*(1,36) = 7.418, *p* = 0.01, η_p_^2^ = 0.171, BF_incl_ = 3.343, with the mean amplitude being smaller in Experiment 1a. The main effect of *Anteriority* and the interaction were not significant.

#### Source Localization

For the Distractor Go *minus* Frequent Go difference within the groups we report the results similarly as in Experiment 1. In Experiment 1a similar posterior areas (lateral occipital area, fusiform gyrus, lingual gyrus, precuneus) disclosed stronger activity to the infrequent distractors than in Experiment 1, and again these areas concentrated on the right side (see Figure 6B and Supplementary Table 1 for the within-subject comparisons and Figure 7B and Supplementary Table 2 for the comparison between the two groups).

### Discussion

Event-related potentials to the Go and Nogo stimuli in the later latency range were fairly similar in the two experiments.

As the ERP results showed, the distractors in Experiment 1a elicited qualitatively similar results as the facial distractors in Experiment 1, but the magnitude of the differences was smaller (the evidence for the difference was moderate with BF_incl_ larger than 3 but smaller than 10). Accordingly, even if the distractors of Experiment 1a were considered as threatening objects (e.g., Zsido et al., 2019), they were less salient than the emoticons of Experiment 1. However, we do not discard the possibility that face-specific activity (N170) contributed to the effects on the posterior locations. Concerning the anterior positivity, face specific positive activity concentrated on the central locations (e.g., Jeffreys, 1996), whereas distractor-related activity was clearly frontal, i.e., more anterior than the face-specific component.

Source localization results have two facets. First, similar visual areas had increased activity when the distractors were not facial stimuli. Accordingly, the Distractor Go *minus* Frequent Go activity difference did not confine to a specific type of distractor (i.e., face). Second, facial stimuli were more salient distractors than potentially threatening stimuli of Experiment 1a, and this difference resulted in larger activity in areas involved in executive functions (orbitofrontal cortex) and emotional processing (insula). Therefore, we suppose that the increased activity in the 150–168 ms time interval is mainly due to the increased distractor salience.

## Experiment 2

In Experiment 2 the choice RT task was replaced by a simple RT task, in which participants had to give the same response to all Go stimuli. In Experiment 1 and 1a the same distractors appeared on Go stimuli that required different responses depending on the color change. Therefore, the distractors interfered with the task-related features, making the choice more difficult. In a simple RT paradigm the task is to respond to any change, irrespective of the presence or absence of the distractors. In principle, a more salient stimulus change may even facilitate the reaction speed. However, another possibility is that the increased processing load may impede reaction. As for the ERP results, similar within-group Distractor Go *minus* Frequent Go differences, and similar age-related differences in Experiments 1 and 2 would be evidence that the anterior positivity and posterior negativity are not related to discrimination demand, but are signatures of the automatic processing of the additional objects. Age-related differences in this case can be signatures of a more intensive process, i.e., decreased resistance to the processing of otherwise task-unrelated events.

### Methods

#### Participants

Twenty-two older (13 females; mean age 67.91 years, *SD* = 3.23 years) and twenty younger adults (12 females; mean age 21.53 years, *SD* = 2.23 years) participated in the study. Two participants from the older group were excluded because of a technical error at the time of recording. Two further participants from the older group and one participant from the younger group were excluded for low performance, and the data of three additional participants from the younger group were excluded for having too few epochs. Thus, the data of 18 participants (10 females; mean age 67.67 years, *SD* = 3.45 years) in the older group and 16 participants (8 females; mean age 21.56 years, *SD* = 2.09 years) in the younger group were analyzed. All had normal or corrected to normal vision, and no one reported any neurological or psychiatric diseases. One participant from the younger group was left-handed. The participants received payment for their participation. The average IQ of the older and the younger group was 132.22 (*SD* = 9.12) and 106.31 (*SD* = 17.42), respectively^6^.

#### Stimuli and Procedure

The stimuli and procedure were similar to those in Experiment 1 with the following exceptions (see also Figure 1). The dark gray disk changed luminosity instead of color from dark to light gray (RGB 0.6, 0.6, 0.6, 26.4 cd/m^2^). The participants had to press the Space key as fast as possible when the change occurred. The time limit for a key press was 370 ms for the younger group and 420 ms for the older group, which kept the task performance in both groups at an average of 85%. The Distractor Go trials had the same schematic faces appear in addition to the luminosity change as in Experiment 1. On the Nogo trials there was no change in luminosity, and the bin disappeared. The background of the screen was light gray (RGB 0.9, 0.9, 0.9; 88.45 cd/m2). Because the task was a simple RT task, the RT limit for trial removal (RT that was too fast) was lowered to 100 ms.

### Results

#### Behavioral Results

A total of 106 trials in the younger group and 31 trials in the older group were removed for RT that was too fast. Overall performance was 86.85% (*SD* = 4.82%) in the younger group and 87.92% (*SD* = 5.17%) in the older group, and there was no difference between the groups, *t*(32) = 0.601, *p* = 0.552, *Cohen’s d* = 0.213, BF_10_ = 0.379, with median posterior δ = 0.153. 95% credible interval = [−0.427, 0.765].

Experiment 2 had only one type of errors – misses. The rate of misses for Go trials (Figure 2C and Table 1) was again very low, thus it was not further analyzed. Mean RTs for Frequent Go and Distractor Go trials (Figure 2C and Table 1) were compared between the age groups with a mixed ANOVA with *Trial Type* (Frequent Go, Distractor Go) as a within-subject factor and *Age* (younger, older) as the between-subject factor. There was only a main effect of *Age*, *F*(1,32) = 54.37, *p* < 0.001, η_p_^2^ = 0.629, BF_incl_ = 4.007 × 10^5^. The older participants were overall slower than the younger participants. The main effect of *Trial Type* and the interaction were not significant.

The older participants had a higher rate of correct omissions (Figure 2C and Table 1) than the younger participants in the Nogo trials, Mann–Whitney *U* = 60, *p* = 0.004, BF_10_ = 7.269, with median posterior δ = 0.805. 95% credible interval = [−0.141, 1.529].

#### Event-Related Potentials

The morphologies of the ERPs for Frequent Go, Distractor Go, Nogo trials and the Distractor Go *minus* Frequent Go difference potentials were similar to the ERP results of Experiment 1 and Experiment 1a (Figures 3C, 4C, 5C).

##### Go trials

According to the visual inspection of the Frequent Go and the Distractor Go ERPs (Figure 3C), the negativity in the 100–200 ms range at the three occipital ROIs was observed in both groups with a larger amplitude for the Distractor Go compared to Frequent Go trials in the older group. At the frontal ROI the anterior positivity within the 100–200 ms range and the N2b components can be observed in the younger group. In the older group the anterior positivity seemed larger for Distractor than Frequent Go trials. The large frontocentral positivity in the older group for Distractor Go trials was similar to that found in Experiment 1 which suggests N2b emergence that can be observed only as a negative deflection.

###### Posterior negativity

In the younger group the consecutive one-sample *t*-tests showed significant differences at the left parieto-occipital and right parieto-occipital ROIs. In the older group, there were significant consecutive differences in the negative direction for all three ROIs: occipital, right parieto-occipital and left parieto-occipital (see Table 3 for the time windows and Figure 4C for the ranges). At the latter the negativity in the investigated time window merges into a late long-lasting negativity until the end of the epoch. A mixed ANOVA with within-subject factor *Laterality* (posterior, left posterior, right posterior ROI) and between-subject factor *Age* (younger, older) on peak latencies (Table 4) showed a significant but inconclusive main effect of *Age*, *F*(1,32) = 4.772, *p* = 0.036, η_p_^2^ = 0.13, BF_incl_ = 1.970. The peak latency was longer in the older group. The results for the main effect of *Laterality*, and there was moderate evidence for the null hypothesis, *F*(1,32) = 0.556, *p* = 0.576, η_p_^2^ = 0.017, BF_incl_ = 0.14. The interaction was not significant The same analysis on mean amplitudes (Table 4) showed both a main effect of *Age*, *F*(1,32) = 19.063, *p* < 0.001, η_p_^2^ = 0.373, BF_incl_ = 176.126, and of *Laterality*, *F*(2,64) = 20.32, *p* < 0.001, ε = 0.822, η_p_^2^ = 0.388, BF_incl_ = 1.08 × 10^5^. The mean amplitude was larger in the older group compared to the younger group, larger at the left and the right parieto-occipital ROIs compared to the occipital (both *p*s < 0.001). The interaction was not significant, with moderate evidence for a null effect, *F*(1,32) = 0.763, *p* = 0.72, η_p_^2^ = 0.008, BF_incl_ = 0.179.

###### Anterior positivity

The consecutive one-sample *t*-tests showed significant differences at the frontal ROI and at the central ROI in the older group. There were no significant differences in the younger group that last for at least 11 data points (20 ms) within the investigated time windows (see Table 3 for the time windows and Figure 4C for the ranges). Peak latencies (Table 4) were compared with a mixed ANOVA with a within-subject factor *Anteriority* (frontal ROI, central ROI) and a between-subject factor *Age* (younger, older). Only the main factor of *Anteriority* was significant but not conclusive, *F*(1,32) = 4.951, *p* = 0.033, η_p_^2^ = 0.134, BF_incl_ = 1.851, with a longer latency at the frontal ROI. The main effect of *Age* and the interaction were not significant. Mean amplitudes (Table 4) were compared with the same factors. The main effect of *Age* was significant, *F*(1,32) = 30.003, *p* < 0.001, η_p_^2^ = 0.484, BF_incl_ = 3202.887. The mean amplitude was larger in the older group. The main effect of *Anteriority* was not significant, and there was moderate evidence for the lack of interaction, *F*(1,32) = 0.06, *p* = 0.808, η_p_^2^ = 0.002, BF_incl_ = 0.327.

###### N2b

The consecutive one-sample *t*-tests on the Distractor Go *minus* Frequent Go difference potentials indicated a significant difference at the frontal ROI and at the central ROI in the younger group. In the older group the significant differences did not reveal an emergence of N2b, and the time window included only the start of a long-lasting positivity at the frontal ROI (∼300 ms) and at the central ROI (∼350 ms) that lasts until the end of the epoch (see Table 3 for the time windows and Figure 4C for the ranges). A mixed ANOVA with a within-factor *Anteriority* (frontal ROI, central ROI) and a between-subject factor *Age* (younger, older) revealed no significant main effects, and no significant interaction for peak latency (Table 4). There was moderate evidence for no main effect of *Anteriority*, *F*(1,32) = 0.612, *p* = 0.44, η_p_^2^ = 0.019, BF_incl_ = 0.319. The peak-to-peak amplitudes comparison (Table 5) with the same factors showed no significant main effects and no significant interaction, and again there was moderate evidence for no main effect of *Anteriority*, *F*(1,32) = 0.187, *p* = 0.668, η_p_^2^ = 0.006, BF_incl_ = 282.

##### Nogo trials

The Nogo P2 and Nogo P3 emergence was similar to that in Experiment 1 (Figure 5C).

###### Nogo N2

The Nogo N2 was observed only at the parietal ROI (Figure 5C). There was no difference between the groups for peak latencies or mean amplitudes (Table 4).

###### Nogo P3

Peak latencies and mean amplitudes (Table 4) were compared between groups with a mixed ANOVA with a within-subject factor *Anteriority* (central ROI, parietal ROI) and a between-subject factor *Age* (younger, older). The main effects of *Age* and *Anteriority* were significant, *F*(1,32) = 26.093, *p* < 0.001, η_p_^2^ = 0.449, BF_incl_ = 1175.672 and *F*(1,32) = 28.024, *p* < 0.001, η_p_^2^ = 0.467, BF_incl_ = 7519.774, respectively. P3 had a shorter latency in the younger group compared to the older group and at the central ROI compared to the frontal ROI. The interaction was not significant. In the case of mean amplitudes, there was a significant main effect of *Age*, *F*(1,32) = 8.414, *p* = 0.007, η_p_^2^ = 0.208, BF_incl_ = 7.554, and of *Anteriority*, *F*(1,32) = 53.892, *p* < 0.001, η_p_^2^ = 0.627, BF_incl_ = 1.103 × 10^6^. The Nogo P3 was larger in the younger group compared to the older group and larger at the central ROI compared to the frontal ROI. The analysis revealed moderate evidence for the lack of interaction, *F*(1,32) = 0.003, *p* = 0.957, η_p_^2^ < 0.001, BF_incl_ = 0.329.

#### Source Localization

The differences between the Distractor Go and the Frequent Go trials within the groups, reported similarly to those of Experiment 1 and Experiment 1a, encompassed similar posterior structures (fusiform and lingual gyri, lateral occipital areas, precuneus and inferior parietal cortex), and concentrated on the right side (see Figure 6C and Supplementary Table 1).

Experiment 1 and Experiment 2 differed in the duration of differences in the comparison between the two age groups which had a wider time range in Experiment 2, including both the 150–168 and 170–188 ms ranges. However, similar to Experiment 1, the difference between the older and younger group involved these posterior structures (fusiform and lingual gyri, pericalcarine area, precuneus, lateral occipital cortex, and cuneus) as well as some temporal and parietal areas. In the anterior cortex differences appeared in rostral middle frontal gyrus, medial orbitofrontal gyrus (see Figure 7C and Supplementary Table 2).

### Discussion

The aim of Experiment 2 was to investigate the relationships between the ERP effects of the infrequent (distractor) events and the task-demands in the younger and older groups. In the simple RT task of Experiment 2 we obtained similar amplitude differences between the infrequent and frequent stimuli, and these differences were similar to the differences in the choice RT task. Concerning the location of the infrequent minus frequent difference, apart from the longer duration, the activity of the posterior structures was similar to that of Experiment 1. Accordingly, the activation of these structures was unrelated to task-related discrimination demand, which was absent in Experiment 2. Again, in the older group the recruitment of anterior structures was stronger. As for the anterior positivity, posterior negativity, and Nogo components, they were similar in Experiment 1 and 2.

## General Discussion

In the present study our aim was to investigate whether distractor events, presented simultaneously and in the same location as the target stimuli, were more disruptive to performance, and/or resulted in different processing in older and younger participants. The task we introduced required speeded reaction, i.e., a condition with supposed disadvantage for the elderly.

Concerning the question whether the older participants were more sensitive to infrequent salient task-irrelevant stimuli, the answer is “No.” RT was longer in our older group which corresponded to the frequent result of age-related slowing (Cerella, 1985; Salthouse, 1996; Swearer and Kane, 1996; Hedge et al., 2018). However, we obtained no interaction between age and the presence/absence of a distractor in either the choice RT (Experiment 1) or the simple RT (Experiment 2) task, and while the evidence for a null effect is inconclusive, in both cases the differences were very small (∼2.3 ms in Experiment 1 and ∼4 ms in Experiment 2). In Experiment 2 older participants even outperformed the younger group in correct response inhibition to Nogo stimuli. It should be noted that in the more demanding choice RT task the lack of age-related difference did not mean an absence of distractor effects as in both age-groups RT was longer to the infrequent distractors. This result is not surprising, because results on age-related difference to distraction is mixed. Some studies obtained increased sensitivity to task-irrelevant (distractor) stimuli in older adults (e.g., Juola et al., 2000; Wascher et al., 2012; Madden et al., 2014), whereas it was absent in other studies (e.g., Tales et al., 2002; Lien et al., 2011; Leiva et al., 2015).

Concerning the question about differences in the processing of potential distractors, the answer is “Yes.” As an unexpected result of Experiment 1, replicated in Experiment 2, we obtained a robust ERP difference to the Distractor Go (infrequent) vs. Frequent Go stimuli between the older and younger group, i.e., increased anterior positivity and posterior negativity in the 100–200 ms range in older adults. ERPs in this range are a composite of various activities, like subcomponents of posterior N1 (Di Russo et al., 2002), N170 (Experiments 1 and 2 in Bentin et al., 1996). The onset of attention-related components, the selection negativity and positivity (e.g., Harter and Guido, 1980; Wijers et al., 1989; Kenemans et al., 1993; Smid et al., 1999; Talsma et al., 2006), is usually later than 200 ms. Furthermore, Talsma et al. (2006) reported no age-related differences in non-spatial selection negativity. In Experiment 1a (non-facial distractors) we obtained a posterior negativity and an anterior positivity that were considerably smaller than those to facial distractors (Experiments 1 and 2), but did not disappear, therefore we conclude that while face-specific activity may contribute to the increased posterior negativity that resulted in a larger ERP difference between the infrequent and frequent stimuli, that difference is not entirely a face-specific effect, and the difference in amplitude was due to the salience of the stimuli. We propose that the posterior negativity/anterior positivity is a signature of the automatic processing of the additional objects superimposed on the target-related stimulus change. We used the “posterior negativity” as a neutral term for the negativity in the 100–200 ms latency range.

Over the anterior locations the range of increased positivity is within the P2 range. P2 is considered to be dependent on stimulus probability (Luck and Hillyard, 1994), and equal stimulus probability could be responsible for the lack of amplitude increase in older groups in case of spatial visual distraction (e.g., Karthaus et al., 2018). Importantly, in a novelty oddball paradigm Riis et al. (2009) obtained an increased P2 to novel stimuli in older participants in a task that required visual discrimination, but not in a task with irrelevant stimuli (see also Daffner et al., 2015 for an increased anterior P2 to novel stimuli in older groups). Riis et al. (2009) interpreted the increased P2 as an index of ‘motivation saliency.’ As in the case of posterior negativity, in this study we prefer the neutral term ‘anterior positivity.’ This is because the results of the source analysis did not indicate the increase of a distinct positive component to the infrequent stimuli.

As the sLORETA source analysis indicated, the posterior negativity and the anterior positivity were the consequence of the activity of a broad posterior network, including various areas of the ventral stream of visual processing. These areas were more active in the older group, and this activity was independent of the discrimination requirement of the choice RT task and the lack of this requirement in the simple RT task. This network appeared to be sensitive to stimulus saliency, as indicated by larger activity to facial distractor stimuli. In the case of facial distractors some anterior structures increased activity (Experiment 1: caudal middle frontal, lateral orbitofrontal cortex and the insula; Experiment 2: cingulate areas, superior frontal cortex) showing the involvement of central executive and emotional structures. Similar differences appeared in the comparison of Experiment 1 and 1a in the older groups, i.e., the activity in the orbitofrontal cortex and in the insula was larger to facial distractors. Brain imaging studies disclosed the neural networks at some distractor tasks (e.g., Salo et al., 2017; Han et al., 2018), but little is known about the relationships between the surface ERP effects and the putative networks underlying distraction. One possible interpretation of our results is that both the ERPs and the activity of the areas reflect to some extent the activation of a right-lateralized ventral attention system (e.g., Fox et al., 2006; Seeley et al., 2007). However, this interpretation should be treated with caution because of the exploratory nature of the source localization analysis.

Age-related differences on later, attention-related components (N2b, late positivity) are frequently reported (see Daffner et al., 2015 for a comprehensive analysis and discussion). Concerning N2b in Experiment 1, we identified a distinct negativity only in the younger groups. This is because in the older groups the putative anterior N2b is masked by the long-lasting anterior positivity. In the later latency ranges the ERP morphologies were highly different in the age groups. In all experiments the ERPs in the older groups were characterized by a long-lasting anterior positivity. Alperin et al. (2015) obtained a similar positivity in older and middle-aged participants. In the present study we obtained a posterior negative counterpart of the long-lasting anterior positivity. Alperin et al. (2015) reported decreased positivity over the centroparietal regions in elderly, but the definite occipital negativity of the present study was absent in their study. However, the paradigms of the two studies are different. As a general statement, distractors had long-lasting effects in older adults, without considerable decrease of task performance.

As offset responses (task-independent disappearance of an object), Nogo stimuli did not elicit considerable exogenous components. These stimuli elicited a late positivity that was largest over the central location. This positivity had a shorter latency and a larger amplitude in the younger group, showing the frequently reported age-related delay (e.g., Falkenstein et al., 2002). Being a rare event with almost perfect performance, the late positivity seems to be a P3b, rather than a characteristic Nogo P3b.

Concerning the robust age-related differences between ERPs to frequent and infrequent events, together with the similar distractor effects on the behavior level, we have to be careful in generalizing these results to similar situations. In this laboratory task (like in all other studies of the field) the distractors were infrequent events, but they were repeatedly presented, i.e., their appearance was uncertain, but they were not absolutely unexpected events. In everyday situations distracting events are frequently unique ones. Therefore, the large age-related processing difference as indicated by the anterior positivity/posterior negativity in a relatively early latency range, and the long-lasting ERP differences to salient events is a warning that distractors in everyday situations are more hazardous for older people.

## Data Availability Statement

The datasets presented in this study can be found in online repositories. The names of the repository/repositories and accession number(s) can be found below: Open Science Framework, https://osf.io/az97j.

## Ethics Statement

The studies involving human participants were reviewed and approved by Joint Ethical Review Committee for Research in Psychology (Hungary). The participants provided their written informed consent to participate in this study.

## Author Contributions

PK, IC, and ZG conceived and planned the study. PK coded the experiment, collected the data, and performed the data preprocessing and analysis. BN helped with the data collection and contributed to the data preprocessing and analysis. IC and PK drafted and wrote the manuscript. All the authors discussed the results, and commented on and contributed to the manuscript.

## Conflict of Interest

The authors declare that the research was conducted in the absence of any commercial or financial relationships that could be construed as a potential conflict of interest.

## Footnotes

1 This section essentially summarizes the methodology used in all three experiments in the study. Only the differences will be described for the subsequent experiments.

2 www.vislab.ucl.ac.uk/Cogent/

3 Peak latencies were sometimes found at one of the ends of the time windows. In this case the amplitude of the first local peak before the beginning or after the end of the window was used for the calculation.

4 The data of all experiments was also analyzed with *Gender* (male, female) as an additional between-subject factor because of the imbalance in both groups in Experiment 1. The added factor affected very few of the comparisons, and thus was not considered further. A summary of those results can be found in Supplementary Data Sheet 2.

5 http://neuroimage.usc.edu/brainstorm

6 In our studies IQ tests are used mainly to ensure that the participants in both age groups are within the normal range. However, the difference in the average scores may have influenced performance in this study. To test for this, all behavioral and ERP analyses were performed again with IQ as a covariate. The presence of the covariate did not modify the results. A summary of the results can be found in Supplementary Data Sheet 4.
