# Supplementary material for: Age Effects on Distraction in a Visual Task Requiring Fast Reactions: An Event-Related Potential Study
Source: Front Aging Neurosci. 2020 Nov 26;12:596047. doi: 10.3389/fnagi.2020.596047 (PMC7726357; doi:10.3389/fnagi.2020.596047)
Supplement: Supplementary file 1 [file Data_Sheet_1.PDF]

Statistical Analysis Results

$p < 0.05$ or $BF_{10}/BF_{incl} > 10$ or $BF_{10}/BF_{incl} < 0.1$
$10 > BF_{10}/BF_{incl} > 3$ or $0.333 > BF_{10}/BF_{incl} > 0.1$
$0.1 > p \geq 0.05$
$p \geq 0.1$ or $0.333 < BF_{10}/BF_{incl} <3$

Experiment 1

Behavioral results

Task performance (younger vs. older group)

t	*df*	*p*	*Cohen's d*
-0.76	34	0.453	0.261

BF_{10}	BF_{01}	Median posterior δ	95% CI
0.403	2.481	-0.191	[-0.793, 0.375]

Incorrect key presses

	F	*df1*	*df2*	*p*	ε	η_p^2
Trial Type	3.889	1	34	0.057	-	0.103
Age	2.899	1	34	0.098	-	0.079
Trial Type × *Age*	0.033	1	34	0.857	-	<0.001

Model comparison

Models	P(M)	P(M|data)	BF_M	BF_{10}	BF_{01}	error %
Null model (incl. subject)	0.200	0.193	0.954	1.000	1.000	
Trial Type + Age	0.200	0.273	1.499	1.415	0.707	1.889
Trial Type	0.200	0.233	1.218	1.212	0.825	0.888
Age	0.200	0.223	1.145	1.156	0.865	3.644
Trial Type + Age + Trial Type × Age	0.200	0.079	0.341	0.408	2.450	1.984

Analysis of Effects

Effects	P(incl)	P(incl|data)	BF_{incl}
Trial Type	0.400	0.506	1.219
Age	0.400	0.495	1.162
Trial Type × *Age*	0.200	0.079	0.288

Reaction time

	F	*df1*	*df2*	*p*	ε	η_p^2
Trial Type	162.32	1	34	<0.001	-	0.827
Age	107.07	1	34	<0.001	-	0.759
Trial Type × *Age*	0.57	1	34	0.455	-	0.017

Model comparison

Models	P(M)	P(M|data)	BF_M	BF_{10}	BF_{01}	error %
Null model (incl. subject)	0.200	4.372e-21	1.749e -20	1.000	1.000	
Trial Type + *Age*	0.200	0.724	10.492	1.656e +20	6.039e -21	1.709
Trial Type	0.200	0.276	1.525	6.313e +19	1.584e -20	1.502
Age	0.200	1.018e-9	4.070e -9	2.327e +11	4.297e -12	1.148
Trial Type + *Age* + *Trial Type* × *Age*	0.200	7.191e-12	2.876e -11	1.645e +9	6.080e -10	1.185

Analysis of Effects

Effects	P(incl)	P(incl|data)	BF_{incl}
Trial Type	0.600	1.000	9.268e +10
Age	0.600	1.000	6.552e +8
Trial Type × *Age*	0.200	0.276	1.525

Factors
Trial Type (within-subject factor): Frequent Go, Distractor Go
Age (between-subject factor): younger group, older group

Event-related potentials

Distractor Go *minus* Frequent Go ERP comparisons

Posterior negativity

Peak latency

	F	*df1*	*df2*	*p*	ε	η_p^2
Laterality	0.256	1	34	0.775	0.988	0.007
Age	16.601	1	34	<0.001	-	0.328
Laterality × *Age*	0.822	1	34	0.444	0.988	0.024

Model comparison

Models	P(M)	P(M|data)	BF_M	BF_{10}	BF_{01}	error %
Null model (incl. subject)	0.200	0.009	0.037	1.000	1.000	

Age	0.200	0.871	26.964	93.885	0.011	1.519
Laterality + Age	0.200	0.094	0.417	10.188	0.098	2.893
Laterality + Age + Laterality × Age	0.200	0.024	0.100	2.635	0.380	1.813
Laterality	0.200	9.736e -4	0.004	0.105	9.527	1.493

Analysis of Effects

Effects	P(incl)	P(incl|data)	BF_{incl}
Laterality	0.400	0.095	0.108
Age	0.400	0.965	94.186
Laterality x Age	0.200	0.024	0.259

Mean amplitude

	F	*df1*	*df2*	*p*	ε	η_p^2
Laterality	19.715	1	34	<0.001	0.734	0.367
Age	17.448	1	34	<0.001	-	0.339
Laterality x Age	0.57	1	34	0.518	0.734	0.017

Mean amplitude, *p* values for the comparison between the levels of the *Laterality* factor:
occipital ROI, left parieto-occipital ROI ($p = 0.002$)
occipital ROI, right parieto-occipital ROI ($p < 0.001$)
left parieto-occipital ROI, right parieto-occipital ROI ($p = 0.027$)

Model comparison

Models	P(M)	P(M|data)	BF_M	BF_{10}	BF_{01}	error %
Null model (incl. subject)	0.200	6.947e -8	2.779e -7	1.000	1.000	
Laterality + Age	0.200	0.817	17.896	1.177e +7	8.500e -8	1.381
Laterality + Age + Laterality × Age	0.200	0.176	0.856	2.537e +6	3.941e -7	3.417
Laterality	0.200	0.006	0.026	92150.920	1.085e -5	0.910
Age	0.200	8.906e -6	3.563e -5	128.209	0.008	4.134

Analysis of Effects

Effects	P(incl)	P(incl|data)	BF_{incl}
Laterality	0.400	0.824	91769.900
Age	0.400	0.817	127.674
Laterality × *Age*	0.200	0.176	0.216

Factors
Laterality (within-subject factor): occipital ROI, left parieto-occipital ROI, right parieto-occipital ROI
Age (between-subject factor): younger group, older group

Anterior positivity

Peak latency

	F	*df1*	*df2*	*p*	ε	η_p^2
Anteriority	20.861	1	34	<0.001	-	0.38
Age	0.018	1	34	0.894	-	<0.001
Anteriority × *Age*	10.572	1	34	0.003	-	0.237

Peak latency, *p* values for the comparisons in the interaction:
frontal ROI, younger group vs. central ROI, younger group ($p < 0.001$)
frontal ROI, younger group vs. frontal ROI, older group ($p = 0.139$)
frontal ROI, younger group vs. central ROI, older group ($p = 0.018$)
central ROI, younger group vs. frontal ROI, older group ($p = 0.032$)
central ROI, younger group vs. central ROI, older group ($p = 0.208$)
frontal ROI, older group vs. central ROI, older group ($p = 0.789$)

Model comparison

Models	P(M)	P(M|data)	BF_M	BF_{10}	BF_{01}	error %
Null model (incl. subject)	0.200	0.004	0.016	1.000	1.000	
Anteriority + *Age*	0.200	0.446	3.225	112.903	0.009	1.828
Anteriority + *Age* + *Anteriority* × *Age*	0.200	0.340	2.065	86.124	0.012	3.660
Anteriority	0.200	0.201	1.004	50.735	0.020	3.765
Age	0.200	0.009	0.035	2.176	0.460	0.777

Analysis of Effects

Effects	P(incl)	P(incl|data)	BF_{incl}
Anteriority	0.400	0.647	51.520
Age	0.400	0.455	2.224
Anteriority × *Age*	0.200	0.340	0.763

Mean amplitude

	F	*df1*	*df2*	*p*	ε	η_p^2
Anteriority	4.318	1	34	0.045	-	0.113
Age	24.045	1	34	<0.001	-	0.414
Anteriority × *Age*	7.468	1	34	0.01	-	0.18

Mean amplitude, *p* values for the comparisons in the interaction:
frontal ROI, younger group vs. central ROI, younger group ($p = 0.009$)
frontal ROI, younger group vs. frontal ROI, older group ($p < 0.001$)
frontal ROI, younger group vs. central ROI, older group ($p < 0.001$)
central ROI, younger group vs. frontal ROI, older group ($p = 0.001$)
central ROI, younger group vs. central ROI, older group ($p = 0.001$)
frontal ROI, older group vs. central ROI, older group ($p = 0.967$)

Model comparison

Models	P(M)	P(M|data)	BF_M	BF_{10}	BF_{01}	error %
Null model (incl. subject)	0.200	3.157e -4	0.001	1.000	1.000	
Anteriority + *Age* + *Anteriority* × *Age*	0.200	0.700	9.334	2217.138	4.510e -4	2.405
Age	0.200	0.150	0.706	474.995	0.002	1.287
Anteriority + *Age*	0.200	0.149	0.702	473.046	0.002	1.162
Anteriority	0.200	3.320e -4	0.001	1.051	0.951	0.981

Analysis of Effects

Effects	P(incl)	P(incl|data)	BF_{incl}
Anteriority	0.400	0.150	0.996
Age	0.400	0.299	462.122
Anteriority × *Age*	0.200	0.700	4.687

Factors
Anteriority (within-subject factor): frontal ROI, central ROI
Age (between-subject factor): younger group, older group

N2b

Peak latency

	F	*df1*	*df2*	*p*	ε	η_p^2
Anteriority	1.287	1	34	0.265	-	0.037
Age	4.007	1	34	0.053	-	0.105
Anteriority × *Age*	0.84	1	34	0.366	-	0.024

Model comparison

Models	P(M)	P(M|data)	BF_M	BF_{10}	BF_{01}	error %
Null model (incl. subject)	0.200	0.279	1.547	1.000	1.000	
Age	0.200	0.370	2.350	1.327	0.753	2.646
Anteriority + *Age*	0.200	0.161	0.766	0.576	1.735	2.563
Anteriority	0.200	0.119	0.541	0.427	2.340	1.538

Anteriority + Age + Anteriority × *Age*	0.200	0.071	0.307	0.255	3.915	3.517

Analysis of Effects

Effects	P(incl)	P(incl|data)	BF_{incl}
Anteriority	0.400	0.280	0.431
Age	0.400	0.531	1.334
Anteriority × *Age*	0.200	0.071	0.443

Peak to peak amplitude

	F	*df1*	*df2*	*p*	ε	η_p^2
Anteriority	0.18	1	34	0.679	-	0.005
Age	4.04	1	34	0.052	-	0.106
Anteriority × *Age*	2.658	1	34	0.112	-	0.073

Model comparison

Models	P(M)	P(M|data)	BF_M	BF_{10}	BF_{01}	error %
Null model (incl. subject)	0.200	0.266	1.448	1.000	1.000	
Age	0.200	0.456	3.349	1.714	0.583	7.042
Anteriority + Age	0.200	0.114	0.514	0.428	2.335	5.482
Anteriority + Age + Anteriority × *Age*	0.200	0.096	0.426	0.362	2.764	2.506
Anteriority	0.200	0.068	0.294	0.258	3.882	1.839

Analysis of Effects

Effects	P(incl)	P(incl|data)	BF_{incl}
Anteriority	0.400	0.182	0.253
Age	0.400	0.570	1.704
Anteriority × *Age*	0.200	0.096	0.845

Factors

Anteriority (within-subject factor): frontal ROI, central ROI

Age (between-subject factor): younger group, older group

Nogo ERP comparisons

Nogo N2

	t	*df*	*p*	*Cohen's d*
Peak latency	-1.465	34	0.152	0.488
Mean amplitude	-1.586	34	0.122	0.529

Peak latency

BF_{10}	BF_{01}	Median posterior δ	95% CI
0.736	1.359	0.379	[-0.201, 1.015]

Mean amplitude

BF_{10}	BF_{01}	Median posterior δ	95% CI
0.846	1.182	0.413	[-0.171, 1.055]

Nogo P3

Peak latency

	F	*df1*	*df2*	*p*	ε	η_p^2
Anteriority	10.818	1	34	0.002	-	0.241
Age	22.823	1	34	<0.001	-	0.401
Anteriority × *Age*	4.682	1	34	0.038	-	0.121

Peak latency, *p* values for the comparisons in the interaction:
central ROI, younger group vs. parietal ROI, younger group ($p = 0.003$)
central ROI, younger group vs. central ROI, older group ($p < 0.001$)
central ROI, younger group vs. parietal ROI, older group ($p < 0.001$)
parietal ROI, younger group vs. central ROI, older group ($p = 0.152$)
parietal ROI, younger group vs. parietal ROI, older group ($p = 0.037$)
central ROI, older group vs. parietal ROI, older group ($p = 0.856$)

Model comparison

Models	P(M)	P(M|data)	BF_M	BF_{10}	BF_{01}	error %
Null model (incl. subject)	0.200	5.983e -5	2.393e -4	1.000	1.000	
Anteriority + Age + Anteriority × *Age*	0.200	0.633	6.906	10584.631	9.448e -5	1.678
Anteriority + Age	0.200	0.340	2.058	5677.798	1.761e -4	1.281
Age	0.200	0.026	0.108	441.044	0.002	0.884
Anteriority	0.200	6.324e -4	0.003	10.570	0.095	1.428

Analysis of Effects

Effects	P(incl)	P(incl|data)	BF_{incl}
Anteriority	0.400	0.340	12.868
Age	0.400	0.366	528.862
Anteriority × *Age*	0.200	0.633	1.864

Mean amplitude

	F	$df1$	$df2$	p	ε	η_p^2
Anteriority	65.772	1	34	<0.001	-	0.659
Age	9.103	1	34	0.005	-	0.211
Anteriority × *Age*	1.244	1	34	0.273	-	0.035

Model comparison

Models	P(M)	P(M|data)	BF_M	BF_{10}	BF_{01}	error %
Null model (incl. subject)	0.200	4.396e -9	1.759e -8	1.000	1.000	
Anteriority + *Age*	0.200	0.603	6.082	1.372e +8	7.288e -9	1.446
Anteriority + *Age* + *Anteriority* × *Age*	0.200	0.330	1.967	7.498e +7	1.334e -8	6.696
Anteriority	0.200	0.067	0.288	1.527e +7	6.550e -8	1.043
Age	0.200	1.862e -8	7.449e -8	4.236	0.236	2.783

Analysis of Effects

Effects	P(incl)	P(incl|data)	BF_{incl}
Anteriority	0.400	0.670	2.912e +7
Age	0.400	0.603	8.988
Anteriority × *Age*	0.200	0.330	0.546

Factors
Anteriority (within-subject factor): central ROI, parietal ROI
Age (between-subject factor): younger group, older group

Experiment 1a

Behavioral results

Task performance (Experiment 1 (older group) vs. Experiment 1a)

t	df	p	*Cohen's d*
1.27	36	0.212	0.422

BF_{10}	BF_{01}	Median posterior δ	95% CI
0.593	1.687	-0.321	[-0.931, 0.241]

Incorrect key presses

	F	*df1*	*df2*	*p*	ε	η_p^2
Trial Type	15.26	1	36	<0.001	-	0.298
Experiment	0.213	1	36	0.647	-	0.006
Trial Type × *Experiment*	4.404	1	36	0.043	-	0.109

Incorrect key presses, *p* values for the comparisons in the interaction:
Frequent Go, Experiment 1 (older group) vs. Distractor Go, Experiment 1 (older group) ($p = 0.603$)
Frequent Go, Experiment 1 (older group) vs. Frequent Go, Experiment 1a ($p = 0.99$)
Frequent Go, Experiment 1 (older group) vs. Distractor Go, Experiment 1a ($p = 0.283$)
Distractor Go, Experiment 1 (older group) vs. Frequent Go, Experiment 1a ($p = 0.781$)
Distractor Go, Experiment 1 (older group) vs. Distractor Go, Experiment 1a ($p = 0.648$)
Frequent Go, Experiment 1a vs. Distractor Go, Experiment 1a ($p < 0.001$)

Model comparison

Models	P(M)	P(M|data)	BF_M	BF_{10}	BF_{01}	error %
Null model (incl. subject)	0.200	0.008	0.032	1.000	1.000	
Trial Type	0.200	0.423	2.932	53.580	0.019	1.006
Trial Type + Experiment + Trial Type × *Experiment*	0.200	0.361	2.260	45.735	0.022	2.358
Trial Type + Experiment	0.200	0.205	1.029	25.925	0.039	2.081
Age	0.200	0.004	0.014	0.448	2.235	1.616

Analysis of Effects

Effects	P(incl)	P(incl|data)	BF_{incl}
Trial Type	0.400	0.628	54.925
Experiment	0.400	0.208	0.483
Trial Type × *Experiment*	0.200	0.361	1.764

Reaction time

	F	*df1*	*df2*	*p*	ε	η_p^2
Trial Type	92.38	1	36	<0.001	-	0.72
Experiment	0.96	1	36	0.333	-	0.026
Trial Type × *Experiment*	0.43	1	36	0.515	-	0.012

Model comparison

Models	P(M)	P(M|data)	BF_M	BF_{10}	BF_{01}	error %
Null model (incl. subject)	0.200	1.324e -9	5.297e -9	1.000	1.000	
Trial Type	0.200	0.510	4.170	3.855e +8	2.594e -9	1.017
Trial Type + Experiment	0.200	0.355	2.197	2.677e +8	3.735e -9	1.818
Trial Type + Experiment + Trial Type × Experiment	0.200	0.135	0.625	1.020e +8	9.804e -9	4.596
Age	0.200	6.845e -10	2.738e -9	0.517	1.935	0.997

Analysis of Effects

Effects	P(incl)	P(incl|data)	BF_{incl}
Trial Type	0.400	0.865	4.306e +8
Experiment	0.400	0.355	0.695
Trial Type × Experiment	0.200	0.135	0.381

Factors
Trial Type (within-subject factor): Frequent Go, Distractor Go
Experiment (between-subject factor): Experiment 1 (older group), Experiment 1a

Event-related potentials

Distractor Go *minus* Frequent Go ERP comparisons

Posterior negativity

Peak latency

	F	$df1$	$df2$	p	ε	η_p^2
Laterality	0.345	1	36	0.709	0.926	0.01
Experiment	2.438	1	36	0.127	-	0.063
Laterality × Experiment	0.915	1	36	0.405	0.926	0.024

Model comparison

Models	P(M)	P(M|data)	BF_M	BF_{10}	BF_{01}	error %
Null model (incl. subject)	0.200	0.466	3.489	1.000	1.000	
Age	0.200	0.420	2.892	0.901	1.110	0.892
Laterality	0.200	0.053	0.222	0.113	8.864	1.624
Laterality + Experiment	0.200	0.049	0.204	0.104	9.583	2.872
Laterality + Experiment + Laterality × Experiment	0.200	0.013	0.054	0.028	35.139	3.216

Analysis of Effects

Effects	P(incl)	P(incl|data)	BF $_{incl}$
Laterality	0.400	0.101	0.114
Experiment	0.400	0.468	0.903
Laterality × *Experiment*	0.200	0.013	0.273

Mean amplitude

	F	$df1$	$df2$	p	ε	η_p^2
Laterality	7.943	1	36	0.002	0.803	0.181
Experiment	7.85	1	36	0.008	-	0.179
Laterality × *Experiment*	5.306	1	36	0.012	0.803	0.129

Mean amplitude, p values for the comparisons between the levels of the *Laterality* factor:
occipital ROI, left parieto-occipital ROI ($p = 0.295$)
occipital ROI, right parieto-occipital ROI ($p < 0.001$)
left parieto-occipital ROI, right parieto-occipital ROI ($p = 0.064$)

Mean amplitude, p values for the comparisons in the interaction:
occipital ROI, Experiment 1 (older group) vs. left parieto-occipital ROI, Experiment 1 (older group) ($p = 0.277$)
occipital ROI, Experiment 1 (older group) vs. right parieto-occipital ROI, Experiment 1 (older group) ($p < 0.001$)
occipital ROI, Experiment 1 (older group) vs. occipital ROI, Experiment 1a ($p = 0.936$)
occipital ROI, Experiment 1 (older group) vs. left parieto-occipital ROI, Experiment 1a ($p = 0.943$)
occipital ROI, Experiment 1 (older group) vs. right parieto-occipital ROI, Experiment 1a ($p = 0.99$)
left parieto-occipital ROI, Experiment 1 (older group) vs. right parieto-occipital ROI, Experiment 1 (older group) ($p = 0.065$)
left parieto-occipital ROI, Experiment 1 (older group) vs. occipital ROI, Experiment 1a ($p = 0.19$)
left parieto-occipital ROI, Experiment 1 (older group) vs. left parieto-occipital ROI, Experiment 1a ($p = 0.199$)
left parieto-occipital ROI, Experiment 1 (older group) vs. right parieto-occipital ROI, Experiment 1a ($p = 0.338$)
right parieto-occipital ROI, Experiment 1 (older group) vs. occipital ROI, Experiment 1a ($p = 0.001$)
right parieto-occipital ROI, Experiment 1 (older group) vs. left parieto-occipital ROI, Experiment 1a ($p = 0.001$)
right parieto-occipital ROI, Experiment 1 (older group) vs. right parieto-occipital ROI, Experiment 1a ($p = 0.003$)
occipital ROI, Experiment 1a vs. left parieto-occipital ROI, Experiment 1a ($p = 1.0$)
occipital ROI, Experiment 1a vs. right parieto-occipital ROI, Experiment 1a ($p = 0.996$)
left parieto-occipital ROI, Experiment 1a vs. right parieto-occipital ROI, Experiment 1a ($p = 0.997$)

Model comparison

Models	P(M)	P(M|data)	BF_M	BF_{10}	BF_{01}	error %
Null model (incl. subject)	0.200	0.002	0.007	1.000	1.000	
Laterality + Experiment + Laterality × Experiment	0.200	0.822	18.443	492.129	0.002	2.068
Laterality + Experiment	0.200	0.144	0.672	86.134	0.012	3.727
Laterality	0.200	0.023	0.093	13.545	0.074	0.816
Experiment	0.200	0.010	0.041	6.059	0.165	1.192

Analysis of Effects

Effects	P(incl)	P(incl|data)	BF_{incl}
Laterality	0.400	0.166	14.120
Experiment	0.400	0.154	6.339
Laterality × *Experiment*	0.200	0.822	5.714

Factors
Laterality (within-subject factor): occipital ROI, left parieto-occipital ROI, right parieto-occipital ROI
Experiment (between-subject factor): Experiment 1 (older group), Experiment 1a

Anterior positivity

Peak latency

	F	*df1*	*df2*	*p*	ε	η_p^2
Anteriority	5.523	1	36	0.024	-	0.133
Experiment	2.61	1	36	0.115	-	0.068
Anteriority × *Experiment*	0.586	1	36	0.449	-	0.016

Model comparison

Models	P(M)	P(M|data)	BF_M	BF_{10}	BF_{01}	error %
Null model (incl. subject)	0.200	0.144	0.676	1.000	1.000	
Anteriority	0.200	0.307	1.768	2.122	0.471	1.975
Anteriority + Experiment	0.200	0.292	1.649	2.020	0.495	2.329
Experiment	0.200	0.144	0.673	0.997	1.003	2.632
Anteriority + Experiment + Anteriority × Experiment	0.200	0.113	0.510	0.782	1.278	3.061

Analysis of Effects

Effects	P(incl)	P(incl|data)	BF_{incl}
Anteriority	0.400	0.598	2.075
Experiment	0.400	0.436	0.966
Anteriority × *Experiment*	0.200	0.113	0.387

Mean amplitude

	F	*df1*	*df2*	*p*	ε	η_p^2
Anteriority	1.554	1	36	0.221	-	0.041
Experiment	7.418	1	36	0.01	-	0.171
Anteriority × *Experiment*	3.822	1	36	0.058	-	0.096

Mean amplitude, *p* values for the comparisons in the interaction:
frontal ROI, Experiment 1 (older group) vs. central ROI, Experiment 1 (older group) ($p = 0.961$)
frontal ROI, Experiment 1 (older group) vs. frontal ROI, Experiment 1a ($p = 0.02$)
frontal ROI, Experiment 1 (older group) vs. central ROI, Experiment 1a ($p = 0.093$)
central ROI, Experiment 1 (older group) vs. frontal ROI, Experiment 1a ($p = 0.028$)
central ROI, Experiment 1 (older group) vs. central ROI, Experiment 1a ($p = 0.126$)
frontal ROI, Experiment 1a vs. central ROI, Experiment 1a ($p = 0.111$)

Model comparison

Models	P(M)	P(M|data)	BF_M	BF_{10}	BF_{01}	error %
Null model (incl. subject)	0.200	0.099	0.440	1.000	1.000	
Anteriority + *Experiment* + *Anteriority* × *Experiment*	0.200	0.363	2.280	3.666	0.273	28.949
Experiment	0.200	0.336	2.020	3.388	0.295	3.219
Anteriority + *Experiment*	0.200	0.155	0.732	1.561	0.640	3.682
Anteriority	0.200	0.048	0.200	0.481	2.081	1.006

Analysis of Effects

Effects	P(incl)	P(incl|data)	BF_{incl}
Anteriority	0.400	0.202	0.465
Experiment	0.400	0.490	3.343
Anteriority × *Experiment*	0.200	0.363	2.347

Factors
Anteriority (within-subject factor): frontal ROI, central ROI
Experiment (between-subject factor): Experiment 1 (older group), Experiment 1a

Experiment 2

Behavioral results

Task performance (younger vs. older group)

t	*df*	*p*	*Cohen's d*
0.601	32	0.552	0.214

BF_{10}	BF_{01}	Median posterior δ	95% CI
0.379	2.642	0.153	[-0.427, 0.765]

Correct omissions in the Nogo trials (younger vs. older group)

U	*Z*	*p*	*r*
60	-2.881	0.004	0.494

BF_{10}	BF_{01}	Median posterior δ	95% CI
7.269	0.138	0.805	[-0.141, 1.529]

Reaction time

	F	*df1*	*df2*	*p*	ε	η_p^2
Trial Type	1.69	1	32	0.202	-	0.05
Age	54.37	1	32	<0.001	-	0.629
Trial Type × *Age*	0.78	1	32	0.383	-	0.024

Model comparison

Models	P(M)	P(M|data)	BF_M	BF_{10}	BF_{01}	error %
Null model (incl. subject)	0.200	1.410e -6	5.642e -6	1.000	1.000	
Age	0.200	0.573	5.376	406513.396	2.460e -6	1.228
Trial Type + Age	0.200	0.289	1.629	205140.094	4.875e -6	1.221
Trial Type + Age + Trial Type × *Age*	0.200	0.137	0.637	97337.884	1.027e -5	4.451
Age	0.200	7.422e -7	2.969e -6	0.526	1.900	1.675

Analysis of Effects

Effects	P(incl)	P(incl|data)	BF_{incl}
Trial Type	0.400	0.289	0.505
Age	0.400	0.863	400773.259
Trial Type × *Age*	0.200	0.137	0.474

Factors
Trial Type (within-subject factor): Frequent Go, Distractor Go
Age (between-subject factor): younger group, older group

Event-related potentials

Distractor Go *minus* Frequent Go ERP comparisons

Posterior negativity

Peak latency

	F	*df1*	*df2*	*p*	ε	η_p^2
Laterality	0.556	1	32	0.576	0.907	0.017
Age	4.772	1	32	0.036	-	0.13
Laterality × *Age*	2.685	1	32	0.076	0.907	0.077

Model comparison

Models	P(M)	P(M|data)	BF_M	BF_{10}	BF_{01}	error %
Null model (incl. subject)	0.200	0.272	1.493	1.000	1.000	
Age	0.200	0.533	4.565	1.961	0.510	1.580
Laterality + *Age* + *Laterality* × *Age*	0.200	0.082	0.359	0.303	3.298	3.146
Laterality + *Age*	0.200	0.076	0.327	0.278	3.592	2.548
Laterality	0.200	0.037	0.155	0.137	7.294	0.853

Analysis of Effects

Effects	P(incl)	P(incl|data)	BF_{incl}
Laterality	0.400	0.113	0.140
Age	0.400	0.609	1.970
Laterality × *Age*	0.200	0.082	1.089

Mean amplitude

	F	*df1*	*df2*	*p*	ε	η_p^2
Laterality	20.32	1	32	<0.001	0.822	0.388
Age	19.063	1	32	<0.001	-	0.373
Laterality × *Age*	0.763	1	32	0.72	0.822	0.008

Mean amplitude, *p* values for the comparisons between the levels of the *Laterality* factor:
occipital ROI, left parieto-occipital ROI ($p < 0.001$)
occipital ROI, right parieto-occipital ROI ($p < 0.001$)
left parieto-occipital ROI, right parieto-occipital ROI ($p = 0.975$)

Model comparison

Models	P(M)	P(M|data)	BF_M	BF_{10}	BF_{01}	error %
Null model (incl. subject)	0.200	4.125e -8	1.650e -7	1.000	1.000	
Laterality + Age	0.200	0.844	21.642	2.046e +7	4.888e -8	1.134
Laterality + Age + Laterality × *Age*	0.200	0.151	0.712	3.665e +6	2.729e -7	1.457
Laterality	0.200	0.005	0.019	116162.336	8.609e -6	0.572
Age	0.200	7.818e -6	3.127e -5	189.514	0.005	0.953

Analysis of Effects

Effects	P(incl)	P(incl|data)	BF_{incl}
Laterality	0.400	0.849	107999.217
Age	0.400	0.844	176.126
Laterality × *Age*	0.200	0.151	0.179

Factors
Laterality (within-subject factor): occipital ROI, left parieto-occipital ROI, right parieto-occipital ROI
Age (between-subject factor): younger group, older group

Anterior positivity

Peak latency

	F	*df1*	*df2*	*p*	ε	η_p^2
Anteriority	4.951	1	32	0.033	-	0.134
Age	0.055	1	32	0.816	-	0.002
Anteriority × *Age*	0.943	1	32	0.339	-	0.029

Model comparison

Models	P(M)	P(M|data)	BF_M	BF_{10}	BF_{01}	error %
Null model (incl. subject)	0.200	0.244	1.288	1.000	1.000	
Anteriority	0.200	0.449	3.265	1.845	0.542	1.230
Anteriority + Age	0.200	0.154	0.729	0.633	1.580	1.154
Age	0.200	0.082	0.359	0.338	2.956	1.091
Anteriority + Age + Anteriority × *Age*	0.200	0.070	0.303	0.289	3.463	1.422

Analysis of Effects

Effects	P(incl)	P(incl|data)	BF_{incl}
Anteriority	0.400	0.604	1.851
Age	0.400	0.237	0.341
Anteriority × *Age*	0.200	0.070	0.456

Mean amplitude

	F	*df1*	*df2*	*p*	ε	η_p^2
Anteriority	1.447	1	32	0.238	-	0.043
Age	30.003	1	32	<0.001	-	0.484
Anteriority × *Age*	0.06	1	32	0.808	-	0.002

Model comparison

Models	P(M)	P(M|data)	BF_M	BF_{10}	BF_{01}	error %
Null model (incl. subject)	0.200	1.950e -4	7.803e -4	1.000	1.000	
Age	0.200	0.606	6.160	3108.600	3.217e -4	0.982
Anteriority + Age	0.200	0.297	1.686	1520.452	6.577e -4	7.313
Anteriority + Age + Anteriority × *Age*	0.200	0.097	0.429	496.817	0.002	4.950
Anteriority	0.200	8.684e -5	3.474e -4	0.445	2.246	1.128

Analysis of Effects

Effects	P(incl)	P(incl|data)	BF_{incl}
Anteriority	0.400	0.297	0.489
Age	0.400	0.903	3202.887
Anteriority × *Age*	0.200	0.097	0.327

Factors
Anteriority (within-subject factor): frontal ROI, central ROI
Age (between-subject factor): younger group, older group

N2b

Peak latency

	F	*df1*	*df2*	*p*	ε	η_p^2
Anteriority	0.612	1	32	0.44	-	0.019
Age	1.398	1	32	0.246	-	0.042
Anteriority × *Age*	2.768	1	32	0.106	-	0.08

Model comparison

Models	P(M)	P(M|data)	BF_M	BF_{10}	BF_{01}	error %
Null model (incl. subject)	0.200	0.456	3.346	1.000	1.000	
Age	0.200	0.246	1.308	0.541	1.849	1.006
Anteriority	0.200	0.145	0.676	0.317	3.150	5.513
Anteriority + *Age*	0.200	0.079	0.344	0.174	5.758	2.039
Anteriority + *Age* + *Anteriority* × *Age*	0.200	0.074	0.322	0.163	6.121	2.068

Analysis of Effects

Effects	P(incl)	P(incl|data)	BF_{incl}
Anteriority	0.400	0.224	0.319
Age	0.400	0.325	0.542
Anteriority × *Age*	0.200	0.074	0.941

Peak to peak amplitude

	F	*df1*	*df2*	*p*	ε	η_p^2
Anteriority	0.187	1	32	0.668	-	0.006
Age	0.655	1	32	0.424	-	0.02
Anteriority × *Age*	0.376	1	32	0.544	-	0.012

Model comparison

Models	P(M)	P(M|data)	BF_M	BF_{10}	BF_{01}	error %
Null model (incl. subject)	0.200	0.499	3.981	1.000	1.000	
Age	0.200	0.261	1.415	0.524	1.909	0.833
Anteriority	0.200	0.144	0.674	0.289	3.458	8.250
Anteriority + *Age*	0.200	0.070	0.302	0.141	7.112	1.621
Anteriority + *Age* + *Anteriority* × *Age*	0.200	0.026	0.105	0.051	19.495	1.652

Analysis of Effects

Effects	P(incl)	P(incl|data)	BF_{incl}
Anteriority	0.400	0.214	0.282
Age	0.400	0.331	0.515
Anteriority × *Age*	0.200	0.026	0.365

Factors
Anteriority (within-subject factor): frontal ROI, central ROI
Age (between-subject factor): younger group, older group

Nogo ERP comparisons

Nogo N2

	t	*df*	*p*	*Cohen's d*
Peak latency	-0.751	32	0.458	0.261
Mean amplitude	-0.978	32	0.335	0.335

Peak latency

BF_{10}	BF_{01}	Median posterior δ	95% CI
1.042	0.959	-0.467	[-1.139, 0.139]

Mean amplitude

BF_{10}	BF_{01}	Median posterior δ	95% CI
0.476	2.1	0.253	[-0.329, 0.883]

Nogo P3

Peak latency

	F	*df1*	*df2*	*p*	ε	η_p^2
Anteriority	28.024	1	32	<0.001	-	0.467
Age	26.093	1	32	<0.001	-	0.449
Anteriority × *Age*	1.855	1	32	0.183	-	0.055

Model comparison

Models	P(M)	P(M|data)	BF_M	BF_{10}	BF_{01}	error %
Null model (incl. subject)	0.200	2.027e -7	8.107e -7	1.000	1.000	
Anteriority + *Age*	0.200	0.623	6.617	3.075e +6	3.252e -7	9.674
Anteriority + *Age* + *Anteriority* × *Age*	0.200	0.376	2.412	1.856e +6	5.388e -7	1.832
Anteriority	0.200	5.300e -4	0.002	2615.138	3.824e -4	0.954
Age	0.200	8.275e -5	3.310e -4	408.311	0.002	0.936

Analysis of Effects

Effects	P(incl)	P(incl|data)	BF_{incl}
Anteriority	0.400	0.624	7519.774
Age	0.400	0.623	1175.672

Anteriority × *Age*	0.200	0.376	0.603

Mean amplitude

	F	*df1*	*df2*	*p*	ε	η_p^2
Anteriority	53.892	1	32	<0.001	-	0.627
Age	8.414	1	32	0.007	-	0.208
Anteriority × *Age*	0.003	1	32	0.957	-	<0.001

Model comparison

Models	P(M)	P(M|data)	BF_M	BF_{10}	BF_{01}	error %
Null model (incl. subject)	0.200	1.169e -7	4.676e -7	1.000	1.000	
Anteriority + *Age*	0.200	0.684	8.661	5.852e +6	1.709e -7	4.724
Anteriority + *Age* + *Anteriority* × *Age*	0.200	0.225	1.164	1.928e +6	5.187e -7	2.255
Anteriority	0.200	0.091	0.398	774717.831	1.291e -6	1.301
Age	0.200	5.854e -7	2.342e -6	5.008	0.200	0.914

Analysis of Effects

Effects	P(incl)	P(incl|data)	BF_{incl}
Anteriority	0.400	0.775	1.103e +6
Age	0.400	0.684	7.554
Anteriority × *Age*	0.200	0.225	0.329

Factors
Anteriority (within-subject factor): central ROI, parietal ROI
Age (between-subject factor): younger group, older group
