# Supplementary material for: Age Effects on Distraction in a Visual Task Requiring Fast Reactions: An Event-Related Potential Study
Source: Front Aging Neurosci. 2020 Nov 26;12:596047. doi: 10.3389/fnagi.2020.596047 (PMC7726357; doi:10.3389/fnagi.2020.596047)
Supplement: Supplementary file 2 [file Data_Sheet_2.pdf]

Statistical Analysis Results with *Gender* Added as a Between-subject Factor

The reviewed literature did not include analyses for possible effects of gender, and we did not regard it as a question of interest in our study. However, it is possible to obtain at least some indication of an effect by including gender as a between-subject factor along the rest of the factors in a repeated measures ANOVA. ANOVA is quite robust to unequal sample size, but even if this is seen as a problem, in Experiment 2 the gender of the participants was balanced, so those results can be viewed as more reliable. Note that including one more grouping factor still divides the whole sample into smaller groups, so smaller effects might not be detected.

All results must be viewed as exploratory. Gender as a factor could have a main effect on the results or interact with the other factors. A main effect is not of interest for this study – if, for example, female participants have an advantage at a younger age which they retain as they age, then this suggests similar aging processes for both male and female participants. An interaction on the other hand would mean that there are gender differences in those processes as people age. Out of 33 ANOVAs, only 3 main effects and 4 interactions (only one triple interaction in Experiment 2 for the Nogo P3 mean amplitude) reached significance. Given the exploratory nature of the analysis, these are not sufficient for a reliable interpretation. More importantly, if compared to the statistical analyses without gender as an added factor, almost all originally found effects remain significant. It is possible that the effect of gender is too small to be detected with the current sample size, but this also means that it did not affect the results of this study to a great extent.

($p < 0.05$, $0.05 \leq p < 0.1$, $0.1 \leq p$)

Experiment 1

Behavioral results

Task performance

	F	*df1*	*df2*	*p*	ε	η_p^2
Age	0.251	1	32	0.62	-	0.008
Gender	0.007	1	32	0.934	-	<0.002
Gender × Age	0.041	1	32	0.84	-	0.001

Incorrect key presses

	F	*df1*	*df2*	*p*	ε	η_p^2
Trial Type	6.329	1	32	0.017	-	0.165
Age	5.723	1	32	0.023	-	0.152
Gender	2.939	1	32	0.096	-	0.084
Trial Type × Age	0.968	1	32	0.333	-	0.029
Trial Type × Gender	2.175	1	32	0.15	-	0.064
Age × Gender	2.647	1	32	0.114	-	0.076
Trial Type × Age × Gender	2.218	1	32	0.146	-	0.065

Reaction time

	F	$df1$	$df2$	p	ε	η_p^2
Trial Type	125.55	1	32	<0.001	-	0.796
Age	67.102	1	32	<0.001	-	0.677
Gender	0.362	1	32	.0552	-	0.011
Trial Type × *Age*	0.304	1	32	0.585	-	0.009
Trial Type × *Gender*	1.402	1	32	0.245	-	0.042
Age × *Gender*	0.161	1	32	0.691	-	0.005
Trial Type × *Age* × *Gender*	0.016	1	32	0.899	-	0.0005

Factors
Trial Type (within-subject factor): Frequent Go, Distractor Go
Age (between-subject factor): younger group, older group
Gender (between-subject factor): male, female

Event-related potentials

Distractor Go *minus* Frequent Go ERP comparisons

Posterior negativity

Peak latency

	F	$df1$	$df2$	p	ε	η_p^2
Laterality	0.154	2	64	0.858	0.98	0.005
Age	8.312	1	32	0.007	-	0.206
Gender	4.284	1	32	0.047	-	0.118
Laterality × *Age*	0.805	2	64	0.451	0.98	0.025
Laterality × *Gender*	0.788	2	64	0.459	0.98	0.024
Gender × *Age*	1.544	1	32	0.223	-	0.046
Laterality × *Gender* × *Age*	0.413	2	64	0.664	0.98	0.013

Mean amplitude

	F	$df1$	$df2$	p	ε	η_p^2
Laterality	12.925	2	64	<0.001	0.726	0.288
Age	12.081	1	32	0.002	-	0.274
Gender	0.009	1	32	0.926	-	0.0003
Laterality × *Age*	0.553	2	64	0.524	0.726	0.017

Laterality × Gender	0.289	2	64	0.679	0.726	0.009
Gender × Age	0.035	1	32	0.853	-	0.001
Laterality × Gender × Age	0.372	2	64	0.624	0.726	0.012

Factors
Laterality (within-subject factor): occipital ROI, left parieto-occipital ROI, right parieto-occipital ROI
Age (between-subject factor): younger group, older group
Gender (between-subject factor): male, female

Anterior positivity

Peak latency

	F	*df1*	*df2*	*p*	ε	η_p^2
Anteriority	13.477	1	32	<0.001	-	0.296
Age	0.02	1	32	0.888	-	0.0006
Gender	0.142	1	32	0.709	-	0.004
Anteriority × Age	6.091	1	32	0.019	-	0.16
Anteriority × Gender	0.001	1	32	0.975	-	<0.0001
Gender × Age	0.004	1	32	0.953	-	0.0001
Anteriority × Gender × Age	0.081	1	32	0.777	-	0.003

Mean amplitude

	F	*df1*	*df2*	*p*	ε	η_p^2
Anteriority	0.136	1	32	0.714	-	0.004
Age	18.173	1	32	<0.001	-	0.362
Gender	0.716	1	32	0.404	-	0.022
Anteriority × Age	4.351	1	32	0.045	-	0.12
Anteriority × Gender	7.27	1	32	0.011	-	0.185
Gender × Age	0.204	1	32	0.654	-	0.006
Anteriority × Gender × Age	0.443	1	32	0.51	-	0.014

Factors
Anteriority (within-subject factor): frontal ROI, central ROI
Age (between-subject factor): younger group, older group
Gender (between-subject factor): male, female

N2b

Peak latency

	F	*df1*	*df2*	*p*	ε	η_p^2
Anteriority	0.716	1	32	0.404	-	0.022
Age	0.204	1	32	0.654	-	0.006
Gender	1.123	1	32	0.297	-	0.034
Anteriority × *Age*	1.404	1	32	0.245	-	0.042
Anteriority × *Gender*	0.02	1	32	0.889	-	0.0006
Gender × *Age*	5.701	1	32	0.023	-	0.151
Anteriority × *Gender* × *Age*	0.622	1	32	0.436	-	0.019

Peak to peak amplitude

	F	*df1*	*df2*	*p*	ε	η_p^2
Anteriority	0.004	1	32	0.948	-	0.0001
Age	5.136	1	32	0.03	-	0.138
Gender	0.459	1	32	0.503	-	0.014
Anteriority × *Age*	1.71	1	32	0.2	-	0.051
Anteriority × *Gender*	0.534	1	32	0.47	-	0.016
Gender × *Age*	1.182	1	32	0.285	-	0.036
Anteriority × *Gender* × *Age*	0.001	1	32	0.974	-	<0.0001

Factors
Anteriority (within-subject factor): frontal ROI, central ROI
Age (between-subject factor): younger group, older group
Gender (between-subject factor): male, female

Nogo ERP comparisons

Nogo N2

Peak latency

	F	*df1*	*df2*	*p*	ε	η_p^2
Age	0.572	1	32	0.455	-	0.018
Gender	4.726	1	32	0.037	-	0.129
Gender × *Age*	0.893	1	32	0.352	-	0.027

Mean amplitude

	F	*df1*	*df2*	*p*	ε	η_p^2
Age	0.9	1	34	0.35	-	0.027
Gender	0.869	1	34	0.358	-	0.026
Gender × *Age*	0.411	1	34	0.526	-	0.013

Factors
Age (between-subject factor): younger group, older group
Gender (between-subject factor): male, female

Nogo P3

Peak latency

	F	*df1*	*df2*	*p*	ε	η_p^2
Anteriority	5.5801	1	32	0.024	-	0.149
Age	16.291	1	32	<0.001	-	0.337
Gender	0.432	1	32	0.516	-	0.013
Anteriority × *Age*	4.302	1	32	0.046	-	0.119
Anteriority × *Gender*	0.326	1	32	0.572	-	0.01
Gender × *Age*	0.075	1	32	0.786	-	0.002
Anteriority × *Gender* × *Age*	0.314	1	32	0.579	-	0.01

Mean amplitude

	F	*df1*	*df2*	*p*	ε	η_p^2
Anteriority	43.681	1	32	<0.001	-	0.577
Age	7.267	1	32	0.011	-	0.185
Gender	9.306	1	32	0.005	-	0.226
Anteriority × *Age*	0.23	1	32	0.635	-	0.007
Anteriority × *Gender*	0.0001	1	32	0.991	-	<0.0001
Gender × *Age*	0.016	1	32	0.901	-	0.0005
Anteriority × *Gender* × *Age*	0.596	1	32	0.446	-	0.018

Factors
Anteriority (within-subject factor): central ROI, parietal ROI
Age (between-subject factor): younger group, older group
Gender (between-subject factor): male, female

Experiment 1a

Behavioral results

Task performance

	F	$df1$	$df2$	p	ε	η_p^2
Experiment	1.132	1	34	0.295	-	0.032
Gender	0.255	1	34	0.617	-	0.008
Gender × Experiment	0.038	1	34	0.846	-	0.001

Incorrect key presses

	F	$df1$	$df2$	p	ε	η_p^2
Trial Type	12.154	1	34	0.001	-	0.263
Experiment	0.09	1	34	0.766	-	0.003
Gender	0.804	1	34	0.376	-	0.023
Trial Type × Experiment	3.618	1	34	0.066	-	0.096
Trial Type × Gender	0.236	1	34	0.63	-	0.007
Experiment × Gender	0.954	1	34	0.335	-	0.028
Trial Type × Experiment × Gender	0.251	1	34	0.62	-	0.007

Reaction time

	F	$df1$	$df2$	p	ε	η_p^2
Trial Type	76.39	1	34	<0.001	-	0.692
Experiment	0.27	1	34	0.604	-	0.008
Gender	0.04	1	34	0.834	-	0.001
Trial Type × Experiment	0.06	1	34	0.802	-	0.002
Trial Type × Gender	0.03	1	34	0.868	-	0.001
Experiment × Gender	0.81	1	34	0.374	-	0.023
Trial Type × Experiment × Gender	0.78	1	34	0.384	-	0.022

Factors
Trial Type (within-subject factor): Frequent Go, Distractor Go
Experiment (between-subject factor): Experiment 1, Experiment 1a
Gender (between-subject factor): male, female

Event-related potentials

Distractor Go *minus* Frequent Go ERP comparisons

Posterior negativity

Peak latency

	F	*df1*	*df2*	*p*	ε	η_p^2
Laterality	0.156	2	68	0.856	0.925	0.005
Experiment	1.825	1	34	0.186	-	0.051
Gender	2.064	1	34	0.16	-	0.057
Laterality × *Experiment*	0.49	2	68	0.615	0.925	0.014
Laterality × *Gender*	0.559	2	68	0.574	0.925	0.016
Gender × *Experiment*	0.584	1	34	0.45	-	0.017
Laterality × *Gender* × *Experiment*	0.756	2	68	0.474	0.925	0.022

Mean amplitude

	F	*df1*	*df2*	*p*	ε	η_p^2
Laterality	7.666	2	68	0.003	0.784	0.184
Experiment	6.465	1	34	0.016	-	0.16
Gender	0.001	1	34	0.976	-	<0.0001
Laterality × *Experiment*	4.999	2	68	0.016	0.784	0.128
Laterality × *Gender*	0.579	2	68	0.524	0.784	0.017
Gender × *Experiment*	0.071	1	34	0.791	-	0.002
Laterality × *Gender* × *Experiment*	0.652	2	68	0.489	0.784	0.019

Factors
Laterality (within-subject factor): occipital ROI, left parieto-occipital ROI, right parieto-occipital ROI
Experiment (between-subject factor): Experiment 1, Experiment 1a
Gender (between-subject factor): male, female

Anterior positivity

Peak latency

	F	*df1*	*df2*	*p*	ε	η_p^2
Anteriority	7.794	1	34	0.009	-	0.187
Experiment	2.177	1	34	0.149	-	0.06
Gender	1.559	1	34	0.22	-	0.044
Anteriority × *Experiment*	0.596	1	34	0.445	-	0.017
Anteriority × *Gender*	6.083	1	34	0.019	-	0.152
Gender × *Experiment*	0.778	1	34	0.384	-	0.022
Anteriority × *Gender* × *Experiment*	2.953	1	34	0.095	-	0.08

Mean amplitude

	F	*df1*	*df2*	*p*	ε	η_p^2
Anteriority	0.223	1	34	0.64	-	0.007
Experiment	7.607	1	34	0.009	-	0.183
Gender	0.575	1	34	0.453	-	0.017
Anteriority × *Experiment*	5.469	1	34	0.025	-	0.139
Anteriority × *Gender*	2.286	1	34	0.14	-	0.063
Gender × *Experiment*	0.575	1	34	0.453	-	0.017
Anteriority × *Gender* × *Experiment*	0.485	1	34	0.491	-	0.014

Factors
Anteriority (within-subject factor): frontal ROI, central ROI
Experiment (between-subject factor): Experiment 1, Experiment 1a
Gender (between-subject factor): male, female

Experiment 2

Behavioral results

Task performance

	F	*df1*	*df2*	*p*	ε	η_p^2
Age	0.280	1	30	0.601	-	0.009
Gender	0.016	1	30	0.899	-	0.001
Gender × Age	0.916	1	30	0.346	-	0.03

Correct omissions in the Nogo trials

	F	*df1*	*df2*	*p*	ε	η_p^2
Age	7.659	1	30	0.01	-	
Gender	0.022	1	30	0.882	-	
Gender × Age	0.515	1	30	0.479	-	

Reaction time

	F	*df1*	*df2*	*p*	ε	η_p^2
Trial Type	2.05	1	30	0.163	-	0.064
Age	51.23	1	30	<0.001	-	0.631
Gender	0.01	1	30	0.908	-	0.0005
Trial Type × Age	1.02	1	30	0.32	-	0.033
Trial Type × Gender	1.28	1	30	0.266	-	0.041
Age × Gender	0.66	1	30	0.425	-	0.021
Trial Type × Age × Gender	1.13	1	30	0.295	-	0.036

Factors
Trial Type (within-subject factor): Frequent Go, Distractor Go
Age (between-subject factor): younger group, older group
Gender (between-subject factor): male, female

Event-related potentials

Distractor Go *minus* Frequent Go ERP comparisons

Posterior negativity

Peak latency

	F	*df1*	*df2*	*p*	ε	η_p^2
Laterality	0.455	2	60	0.637	0.913	0.015

Age	4.488	1	30	0.043	-	0.13
Gender	0.029	1	30	0.865	-	0.001
Laterality × *Age*	2.672	2	60	0.077	0.913	0.082
Laterality × *Gender*	0.313	2	60	0.733	0.913	0.01
Gender × *Age*	2.591	1	30	0.118	-	0.08
Laterality × *Gender* × *Age*	1.774	2	60	0.178	0.913	0.056

Mean amplitude

	F	$df1$	$df2$	p	ε	η_p^2
Laterality	21.941	2	60	<0.001	0.778	0.422
Age	21.345	1	30	<0.001	-	0.416
Gender	0.032	1	30	0.86	-	0.001
Laterality × *Age*	0.286	2	60	0.697	0.778	0.009
Laterality × *Gender*	1.424	2	60	0.249	0.778	0.045
Gender × *Age*	4.08	1	30	0.052	-	0.12
Laterality × *Gender* × *Age*	2.922	2	60	0.075	0.778	0.089

Factors
Laterality (within-subject factor): occipital ROI, left parieto-occipital ROI, right parieto-occipital ROI
Age (between-subject factor): younger group, older group
Gender (between-subject factor): male, female

Anterior positivity

Peak latency

	F	$df1$	$df2$	p	ε	η_p^2
Anteriority	4.652	1	30	0.039	-	0.134
Age	0.054	1	30	0.818	-	0.002
Gender	0.142	1	30	0.709	-	0.005
Anteriority × *Age*	0.954	1	30	0.337	-	0.031
Anteriority × *Gender*	0.786	1	30	0.382	-	0.026
Gender × *Age*	0.084	1	30	0.775	-	0.003
Anteriority × *Gender* × *Age*	0.194	1	30	0.663	-	0.006

Mean amplitude

	F	*df1*	*df2*	*p*	ε	η_p^2
Anteriority	1.214	1	30	0.279	-	0.039
Age	32.982	1	30	<0.001	-	0.524
Gender	0.762	1	30	0.39	-	0.025
Anteriority × *Age*	0.115	1	30	0.737	-	0.004
Anteriority × *Gender*	1.638	1	30	0.21	-	0.052
Gender × *Age*	2.75	1	30	0.108	-	0.084
Anteriority × *Gender* × *Age*	0.192	1	30	0.665	-	0.006

Factors
Anteriority (within-subject factor): frontal ROI, central ROI
Age (between-subject factor): younger group, older group
Gender (between-subject factor): male, female

N2b

Peak latency

	F	*df1*	*df2*	*p*	ε	η_p^2
Anteriority	0.459	1	30	0.503	-	0.015
Age	1.185	1	30	0.285	-	0.038
Gender	0.31	1	30	0.582	-	0.01
Anteriority × *Age*	3.275	1	30	0.08	-	0.098
Anteriority × *Gender*	0.136	1	30	0.715	-	0.005
Gender × *Age*	0.437	1	30	0.514	-	0.014
Anteriority × *Gender* × *Age*	3.074	1	30	0.09	-	0.093

Peak to peak amplitude

	F	*df1*	*df2*	*p*	ε	η_p^2
Anteriority	0.127	1	30	0.724	-	0.004
Age	0.68	1	30	0.416	-	0.022
Gender	1.896	1	30	0.179	-	0.059
Anteriority × *Age*	0.445	1	30	0.51	-	0.015
Anteriority × *Gender*	0.6	1	30	0.445	-	0.02

Gender × *Age*	2.153	1	30	0.153	-	0.067
Anteriority × *Gender* × *Age*	0.188	1	30	0.668	-	0.006

Factors
Anteriority (within-subject factor): frontal ROI, central ROI
Age (between-subject factor): younger group, older group
Gender (between-subject factor): male, female

Nogo ERP comparisons

Nogo N2

Peak latency

	F	*df1*	*df2*	*p*	ε	η_p^2
Age	3.115	1	30	0.088	-	0.094
Gender	3.415	1	30	0.074	-	0.102
Gender × *Age*	1.247	1	30	0.273	-	0.04

Mean amplitude

	F	*df1*	*df2*	*p*	ε	η_p^2
Age	0.299	1	30	0.588	-	0.01
Gender	1.292	1	30	0.265	-	0.041
Gender × *Age*	0.052	1	30	0.821	-	0.002

Factors
Age (between-subject factor): younger group, older group
Gender (between-subject factor): male, female

Nogo P3

Peak latency

	F	*df1*	*df2*	*p*	ε	η_p^2
Anteriority	26.414	1	30	<0.001	-	0.468
Age	28.362	1	30	<0.001	-	0.486
Gender	0.135	1	30	0.716	-	0.005
Anteriority × *Age*	1.727	1	30	0.199	-	0.054
Anteriority × *Gender*	0.340	1	30	0.564	-	0.011
Gender × *Age*	3.214	1	30	0.083	-	0.097
Anteriority × *Gender* × *Age*	0.15	1	30	0.701	-	0.005

Mean amplitude

	F	*df1*	*df2*	*p*	ε	η_p^2
Anteriority	59.105	1	30	<0.001	-	0.663
Age	8.108	1	30	0.008	-	0.213
Gender	0.26	1	30	0.614	-	0.009
Anteriority × *Age*	0.022	1	30	0.883	-	0.0007
Anteriority × *Gender*	0.576	1	30	0.454	-	0.019
Gender × *Age*	0.011	1	30	0.916	-	0.0004
Anteriority × *Gender* × *Age*	5.74	1	30	0.023	-	0.161

Factors
Anteriority (within-subject factor): central ROI, parietal ROI
Age (between-subject factor): younger group, older group
Gender (between-subject factor): male, female
