# Supplementary material for: Age Effects on Distraction in a Visual Task Requiring Fast Reactions: An Event-Related Potential Study
Source: Front Aging Neurosci. 2020 Nov 26;12:596047. doi: 10.3389/fnagi.2020.596047 (PMC7726357; doi:10.3389/fnagi.2020.596047)
Supplement: Supplementary file 3 [file Data_Sheet_3.PDF]

A priori power analysis for Experiment 1a

In Experiment 1a we were expressly interested in whether a change of the distracting stimuli from faces to salient threatening objects will result in a change of the large differences between the Frequent Go and the Distractor Go condition that we found in the older group for the anterior positivity and posterior negativity in both Experiment 1 and 2 as well as the stable difference in reaction time (RT) in Experiment 1. The data from both experiments allowed to us to calculate effect sizes – and what sample size would be needed to achieve similar results if we assume that the effect size for the new distractors is as large as that for faces. A non-significant result in Experiment 1a would mean that either the new distractors do not affect the older group in Experiment 1a or their effect is too small to be detected with the current sample, i.e., the new distractors cause a much smaller distraction in the older group. Either way the conclusion would be that faces have a special status as distractors for older participants.

For RT we compared RT for Frequent Go and Distractor Go stimuli in Experiment 1 with a paired-samples *t*-test. For the amplitude difference, we computed the mean amplitude (peak of the grand average ±10 ms) for each component and each ROI for the Distractor Go *minus* Frequent Go difference potential, and then compared it to 0 with a one-sample *t*-test. These tests were calculated only for the older group. Table 1 summarizes the results.

We then used G*Power 3.9.1.4 (Faul et al., 2007) to calculate the necessary sample sizes at different power levels as summarized in Table 2. Even at power 0.99 the largest sample size required was 12. Thus we aimed for a similar sample size to those in Experiment 1 and Experiment 2.

Table 1: Results for the comparisons between the Frequent Go and Distractor Go stimuli

**Comparison**	**Experiment**	**Measurement**	**ROI**	***t***	**N**	***p***	***Cohen's d***
Frequent Go vs. Distractor Go	Experiment 1	RT	-	-6.852	17	<0.001	-1.615
Amplitude against 0 for Distractor Go minus Frequent Go	Experiment 1	anterior positivity	frontal ROI	8.641	17	<0.001	2.037
		anterior positivity	central ROI	10.306	17	<0.001	2.429
		posterior negativity	occipital ROI	-7.166	17	<0.001	-1.689
		posterior negativity	left parieto-occipital ROI	-8.459	17	<0.001	-1.994
		posterior negativity	right parieto-occipital ROI	-8.143	17	<0.001	-1.92
	Experiment 2	anterior positivity	frontal ROI	6.669	17	<0.001	1.572
		anterior positivity	central ROI	9.393	17	<0.001	2.214
		posterior negativity	occipital ROI	-5.881	17	<0.001	-1.386
		posterior negativity	left parieto-occipital ROI	-7.57	17	<0.001	-1.784
		posterior negativity	right parieto-occipital ROI	-9.056	17	<0.001	-2.135

Table 2: Total sample size necessary to obtain effect sizes similar to those in Experiment 1 and Experiment 2 at different levels of power

				**Noncentrality parameter δ**	**Critical *t***	**Total sample size**
**Comparison**	**Experiment**	**Measurement**	**ROI**		**Power 0.8**	
Frequent Go vs. Distractor Go	Experiment 1	RT	-	3.925	2.571	6
Amplitude against 0 for Distractor Go minus Frequent Go difference potential	Experiment 1	anterior positivity	frontal ROI	4.554	2.777	5
		anterior positivity	central ROI	4.858	3.183	4
		posterior negativity	occipital ROI	-3.777	-2.777	5
		posterior negativity	left parieto-occipital ROI	-4.458	-2.777	5
		posterior negativity	right parieto-occipital ROI	-4.293	-2.777	5
	Experiment 2	anterior positivity	frontal ROI	3.85	2.571	6
		anterior positivity	central ROI	4.428	3.183	4
		posterior negativity	occipital ROI	-3.668	-2.447	7
		posterior negativity	left parieto-occipital ROI	-3.99	-2.777	5
		posterior negativity	right parieto-occipital ROI	-4.269	-3.183	4
**Comparison**	**Experiment**	**Measurement**	**ROI**		**Power 0.95**	
Frequent Go vs. Distractor Go	Experiment 1	RT	-	4.532	2.365	8
Amplitude against 0 for Distractor Go minus Frequent Go difference potential	Experiment 1	anterior positivity	frontal ROI	4.99	2.571	6
		anterior positivity	central ROI	5.432	2.777	5
		posterior negativity	occipital ROI	-4.469	-2.447	7
		posterior negativity	left parieto-occipital ROI	-4.884	-2.571	6
		posterior negativity	right parieto-occipital ROI	-4.703	-2.571	6
	Experiment 2	anterior positivity	frontal ROI	4.446	2.365	8
		anterior positivity	central ROI	4.95	2.777	5
		posterior negativity	occipital ROI	-4.159	-2.306	9
		posterior negativity	left parieto-occipital ROI	-4.721	-2.447	7
		posterior negativity	right parieto-occipital ROI	-5.228	-2.571	6
**Comparison**	**Experiment**	**Measurement**	**ROI**		**Power 0.99**	
Frequent Go vs. Distractor Go	Experiment 1	RT	-	5.067	2.262	10
Amplitude against 0 for	Experiment 1	anterior positivity	frontal ROI	5.389	2.447	7
		anterior positivity	central ROI	5.95	2.571	6

		posterior negativity	occipital ROI	-5.067	-2.306	9
		posterior negativity	left parieto-occipital ROI	-5.275	-2.447	7
		posterior negativity	right parieto-occipital ROI	-5.43	-2.365	8
Distractor Go minus Frequent Go difference potential	Experiment 2	anterior positivity	frontal ROI	4.971	2.262	10
		anterior positivity	central ROI	5.857	2.447	7
		posterior negativity	occipital ROI	-4.802	-2.201	12
		posterior negativity	left parieto-occipital ROI	-5.353	-2.306	9
		posterior negativity	right parieto-occipital ROI	-5.647	-2.447	7

References

Faul, F., Erdfelder, E., Lang, A., & Buchner, A. (2007). G*Power 3: A flexible statistical power analysis program for the social, behavioral, and biomedical sciences. *Behavior Research Methods, 39*(2), 175-191. doi:10.3758/bf03193146
