# Supplementary material for: Age Effects on Distraction in a Visual Task Requiring Fast Reactions: An Event-Related Potential Study
Source: Front Aging Neurosci. 2020 Nov 26;12:596047. doi: 10.3389/fnagi.2020.596047 (PMC7726357; doi:10.3389/fnagi.2020.596047)

Statistical Analysis Results for IQ as Covariate

Note the following:

- IQ is measured in our studies mainly as an indicator that the participants in both groups are within the normal range.
- In our study the participants are not from the same population.
- When based on the raw score, IQ scores are biased against the older group.
- When based on standardized scores, in the Hungarian version of the test, IQ scores are biased against the younger group.
- Assumptions that must be met:
    - the dependent variable and the covariate have a similar relationship within the groups (homogeneity of the slopes), i.e., the IQ affects the dependent variable similarly in both younger and older adults;
    - the difference in the population mean is 0, which is uncertain for two groups from different populations (see about possible biases above).
- The IQ scores must be centered in a repeated measures ANCOVA.

Regarding homogeneity of slopes, this assumption can be visually inspected in the figures after the statistical analysis results where each dependent variable is correlated to the centered IQ scores in each group. In most cases the regression lines are parallel or close to parallel, but not in all cases. However, a check of the homogeneity of slopes assumption showed that the condition for rejecting the assumption is not met (i.e., the Age and IQ_centered interaction did not reach significance for any of the analyses). Thus, we assumed homogeneity of the slopes.

Regarding the second assumption, standardized scores should mean that the populations' means do not differ from each other, but the above-mentioned bias suggests that this assumption might not be met. Because of that, the ANCOVA here is used only for testing the main effect of the covariate and the within-subject covariate interactions, while ANOVA is used to test all other remaining effects.

Only the ANCOVA results are reported. The IQ was not a significant covariate in any of the cases. For this reason, we kept the original ANOVA analysis in the manuscript.

($p < 0.05$, $0.05 \leq p < 0.1$, $0.1 \leq p$)

Sources:

Schneider, B. A., Avivi-Reich, M., & Mozuraitis, M. (2015). A cautionary note on the use of the Analysis of Covariance (ANCOVA) in classification designs with and without within-subject factors. Frontiers in psychology, 6, 474. https://doi.org/10.3389/fpsyg.2015.00474

Delaney, H. D. & Maxwell, S. E. (1981). On using analysis of covariance in repeated measures designs, Multivariate Behavioral Research, 16(1), 105-123. http://dx.doi.org/10.1207/s15327906mbr1601_6

Experiment 2

Behavioral results

Task performance

	F	*df1*	*df2*	*p*	ε	η_p^2
Age	0.367	1	31	0.549	-	0.012
IQ_centered	1.571	1	31	0.220	-	0.048

Homogeneity of slopes: p = 0.246 (for the *Age x IQ_centered* interaction in the model)

Correct omissions in the Nogo trials

	F	*df1*	*df2*	*p*	ε	η_p^2
Age	8.06	1	31	0.008	-	0.206
IQ_centered	0.222	1	31	0.641	-	0.007

Homogeneity of slopes: p = 0.375 (for the *Age x IQ_centered* interaction in the model)

Reaction time

	F	*df1*	*df2*	*p*	ε	η_p^2
IQ_centered	0.064	1	31	0.803	-	0.002
Trial Type × *IQ_centered*	0.074	1	31	0.788	-	0.002

Homogeneity of slopes: p = 0.057 (for the *Age x IQ_centered* interaction in the model)

Factors
Trial Type (within-subject factor): Frequent Go, Distractor Go
Age (between-subject factor): younger group, older group
IQ_centered (covariate)

Event-related potentials

Distractor Go *minus* Frequent Go ERP comparisons

Posterior negativity

Peak latency

	F	*df1*	*df2*	*p*	ε	η_p^2
IQ_centered	0.071	1	31	0.792	-	0.002
Laterality × *IQ_centered*	1.056	2	62	0.354	0.932	0.033

Homogeneity of slopes: p = 0.467 (for the *Age x IQ_centered* interaction in the model)

Mean amplitude

	F	*df1*	*df2*	*p*	ε	η_p^2
IQ_centered	3.461	1	31	0.072	-	0.1
Laterality × *IQ_centered*	0.095	2	62	0.873	0.82	0.003

Homogeneity of slopes: p = 0.398 (for the *Age x IQ_centered* interaction in the model)

Factors
Laterality (within-subject factor): occipital ROI, left parieto-occipital ROI, right parieto-occipital ROI
Age (between-subject factor): younger group, older group
IQ_centered (covariate)

Anterior positivity

Peak latency

	F	*df1*	*df2*	*p*	ε	η_p^2
IQ_centered	0.201	1	31	0.657	-	0.006
Anteriority × *IQ_centered*	0.03	1	31	0.863	-	<0.0001

Homogeneity of slopes: p = 0.973 (for the *Age x IQ_centered* interaction in the model)

Mean amplitude

	F	*df1*	*df2*	*p*	ε	η_p^2
IQ_centered	2.418	1	31	0.13	-	0.072
Anteriority × *IQ_centered*	0.331	1	31	0.569	-	0.011

Homogeneity of slopes: p = 0.232 (for the *Age x IQ_centered* interaction in the model)

Factors
Anteriority (within-subject factor): frontal ROI, central ROI
Age (between-subject factor): younger group, older group
IQ_centered (covariate)

N2b

Peak latency

	F	*df1*	*df2*	*p*	ε	η_p^2
IQ_centered	0.002	1	31	0.964	-	<0.0001
Anteriority × *IQ_centered*	0.333	1	31	0.568	-	0.011

Homogeneity of slopes: p = 0.958 (for the *Age x IQ_centered* interaction in the model)

Peak to peak amplitude

	F	*df1*	*df2*	*p*	ε	η_p^2
IQ_centered	2.629	1	31	0.115	-	0.078
Anteriority × *IQ_centered*	0.808	1	31	0.376	-	0.025

Homogeneity of slopes: p = 0.493 (for the *Age x IQ_centered* interaction in the model)

Factors
Anteriority (within-subject factor): frontal ROI, central ROI
Age (between-subject factor): younger group, older group
IQ_centered (covariate)

Nogo ERP comparisons

Nogo N2

Peak latency

	F	*df1*	*df2*	*p*	ε	η_p^2
Age	2.954	1	31	0.096	-	0.087
IQ_centered	0.043	1	31	0.837	-	0.001

Homogeneity of slopes: p = 0.649 (for the *Age x IQ_centered* interaction in the model)

Mean amplitude

	F	*df1*	*df2*	*p*	ε	η_p^2
Age	0.941	1	31	0.339	-	0.029
IQ_centered	0.469	1	31	0.499	-	0.015

Homogeneity of slopes: p = 0.883 (for the *Age x IQ_centered* interaction in the model)

Factors
Age (between-subject factor): younger group, older group
IQ_centered (covariate)

Nogo P3

Peak latency

	F	*df1*	*df2*	*p*	ε	η_p^2
IQ_centered	2.672	1	31	0.112	-	0.079
Anteriority × *IQ_centered*	0.946	1	31	0.338	-	0.03

Homogeneity of slopes: p = 0.144 (for the *Age x IQ_centered* interaction in the model)

Mean amplitude

	F	*df1*	*df2*	*p*	ε	η_p^2
IQ_centered	0.375	1	31	0.545	-	0.012
Anteriority × *IQ_centered*	1.904	1	31	0.177	-	0.058

Homogeneity of slopes: $p = 0.505$ (for the *Age x IQ_centered* interaction in the model)

Factors
Anteriority (within-subject factor): central ROI, parietal ROI
Age (between-subject factor): younger group, older group
IQ_centered (covariate)

Experiment 2

Behavioural data

Task performance

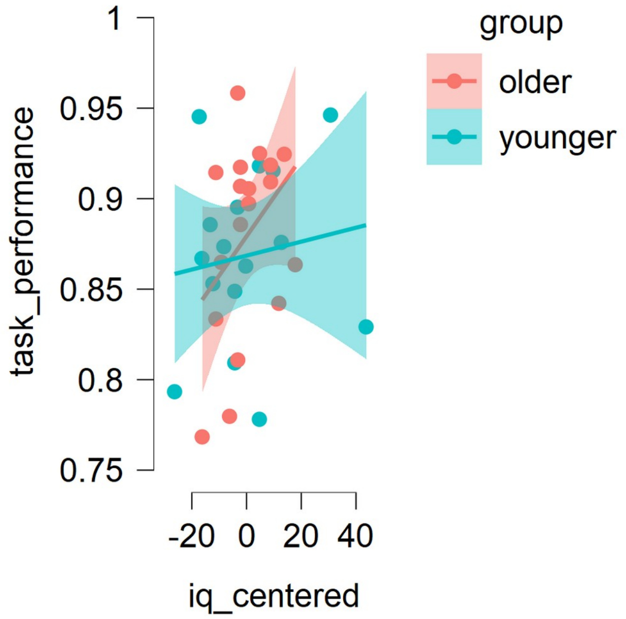

Correct omissions in the Nogo trials

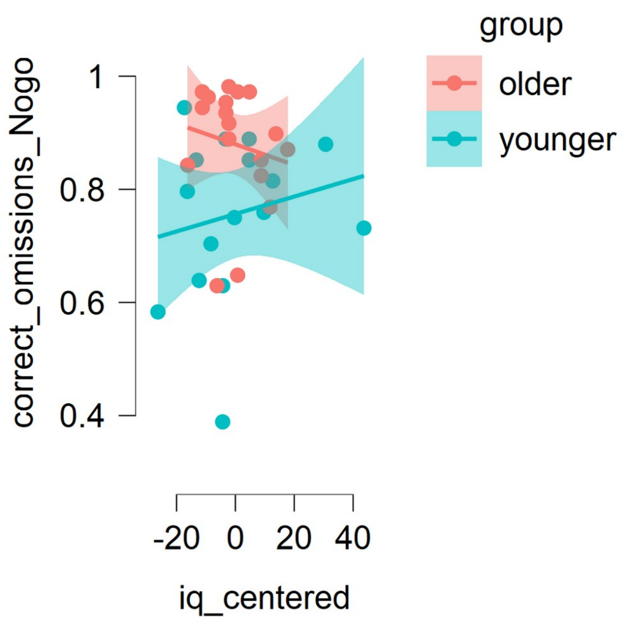

Reaction time for the Frequent Go trials

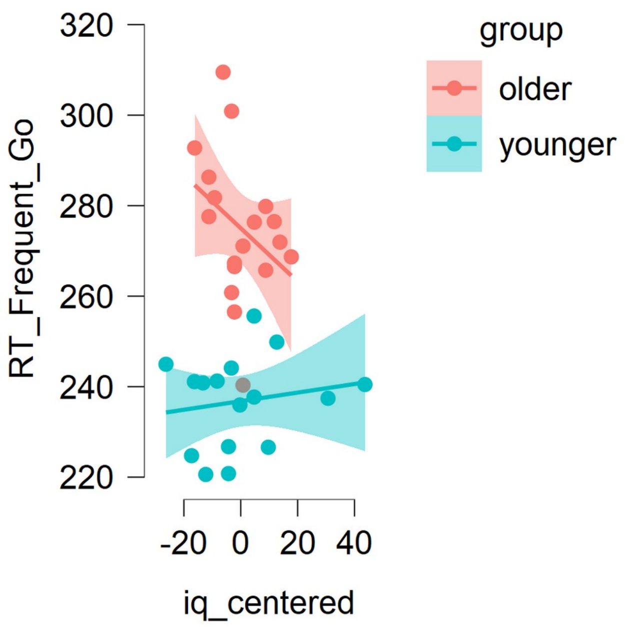

Reaction time for the Distractor Go trials

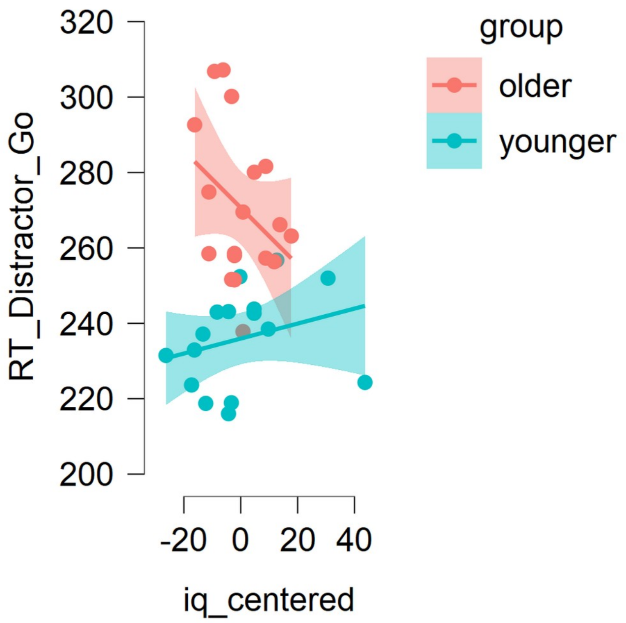

Peak latency

Frequent Go minus Distractor Go difference at the occipital ROI (posterior negativity)

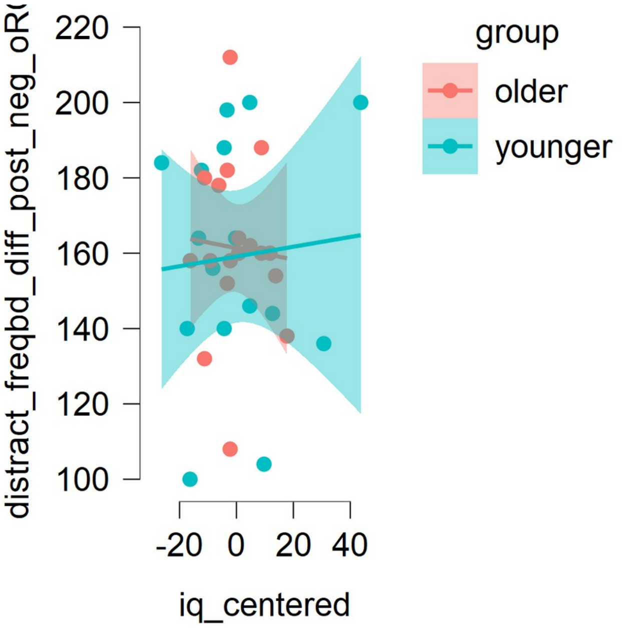

Frequent Go minus Distractor Go difference at the left parieto-occipital ROI (posterior negativity)

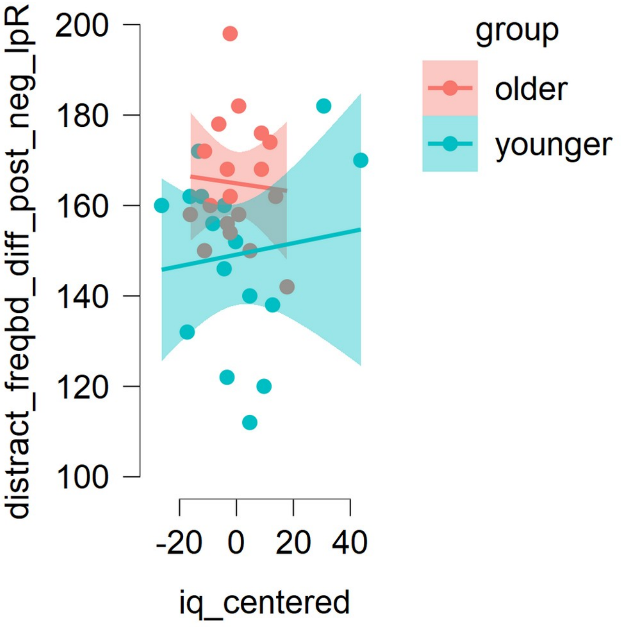

Frequent Go minus Distractor Go difference at the right parieto-occipital ROI (posterior negativity)

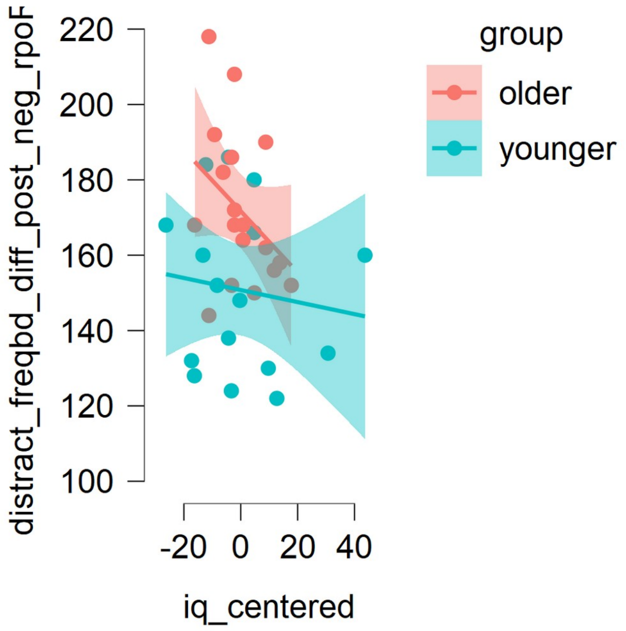

Frequent Go minus Distractor Go difference at the frontal ROI (anterior positivity)

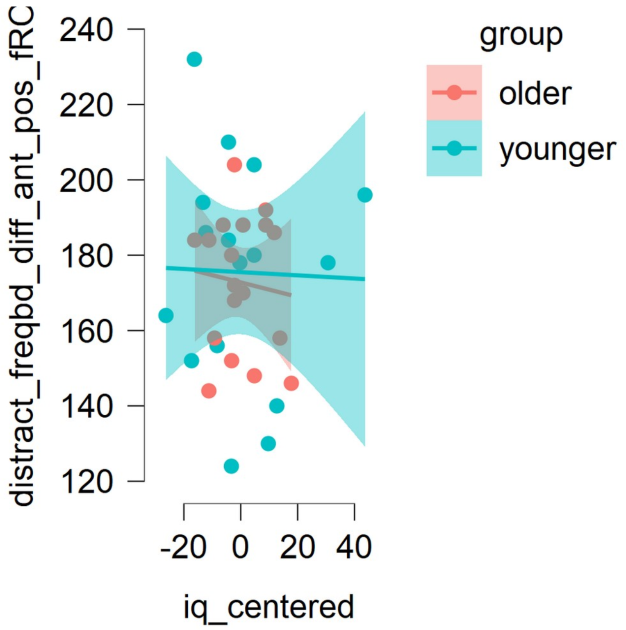

Frequent Go minus Distractor Go difference at the central ROI
(anterior positivity)

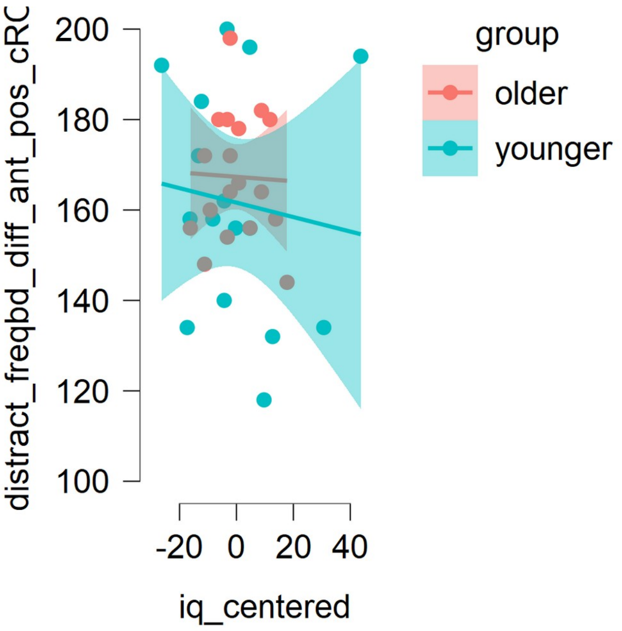

Frequent Go minus Distractor Go difference at the frontal ROI
(N2b)

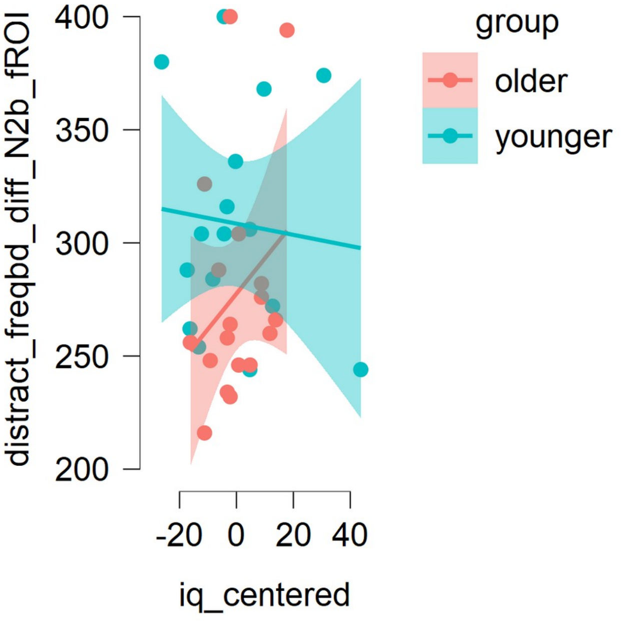

Frequent Go minus Distractor Go difference at the central ROI
(N2b)

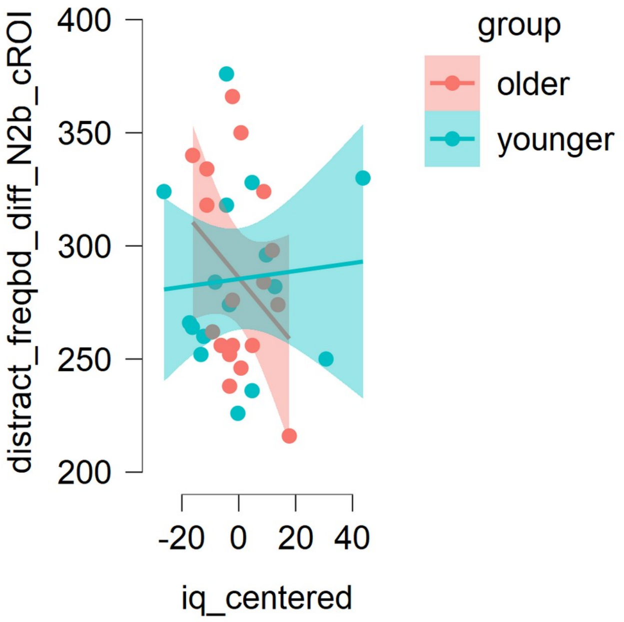

Nogo ERP at the parietal ROI
(Nogo N2)

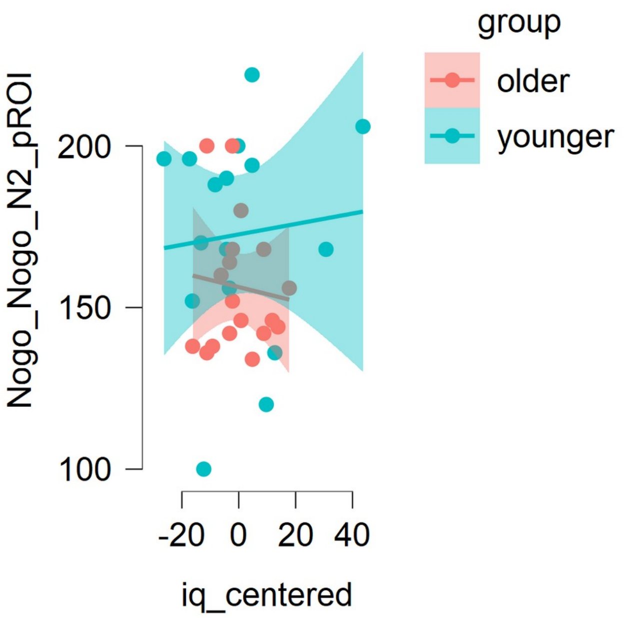

Nogo ERP at the central ROI
(Nogo P3)

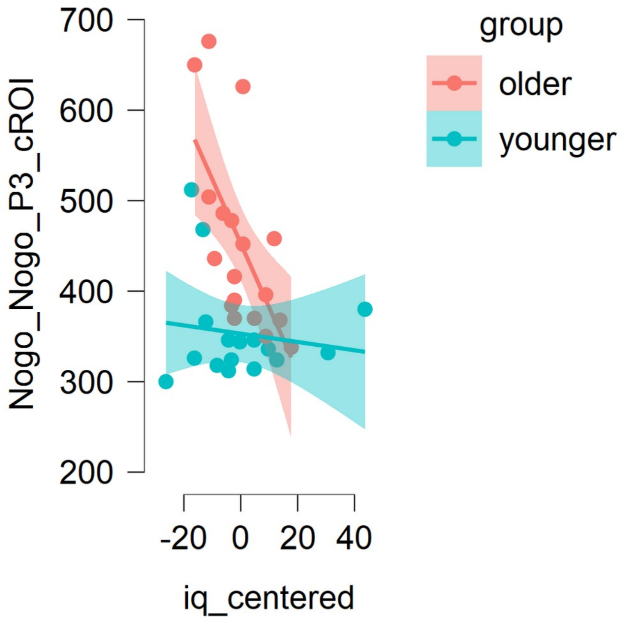

Nogo ERP at the parietal ROI
(Nogo P3)

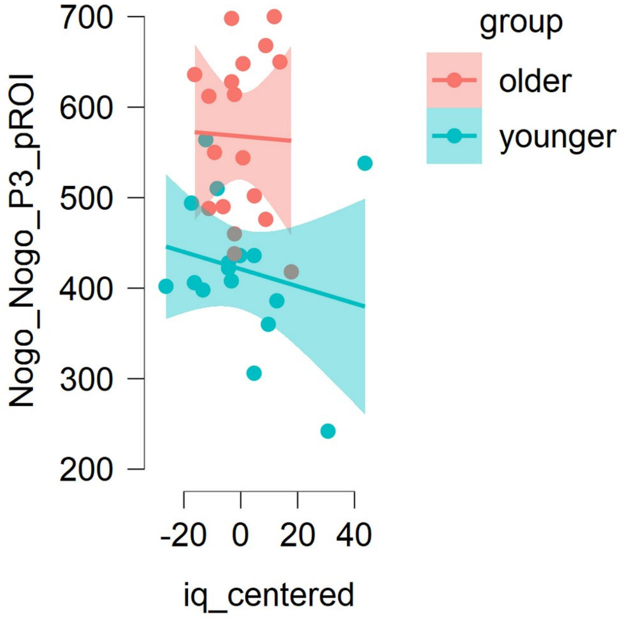

Mean amplitude

Frequent Go minus Distractor Go difference at the occipital ROI
(posterior negativity)

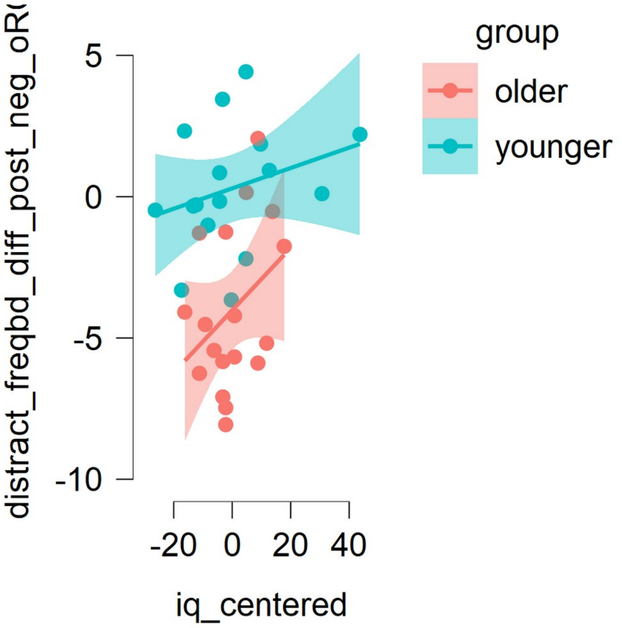

Frequent Go minus Distractor Go difference at the left parieto-occipital ROI
(posterior negativity)

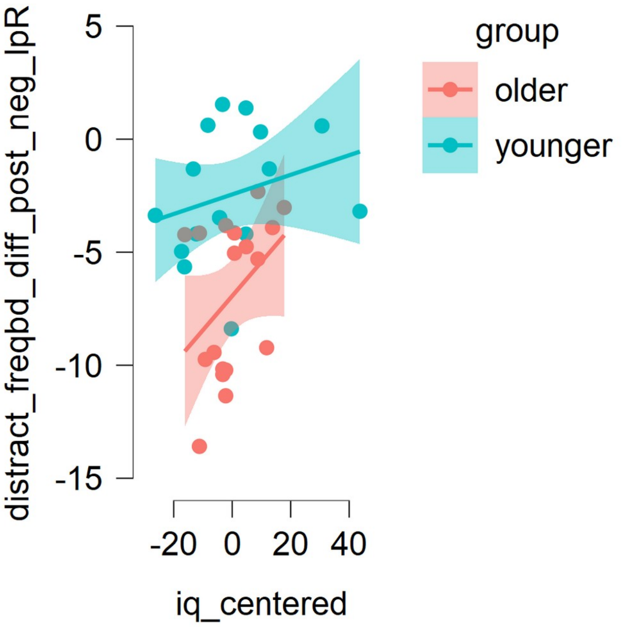

Frequent Go minus Distractor Go difference at the right parieto-occipital ROI (posterior negativity)

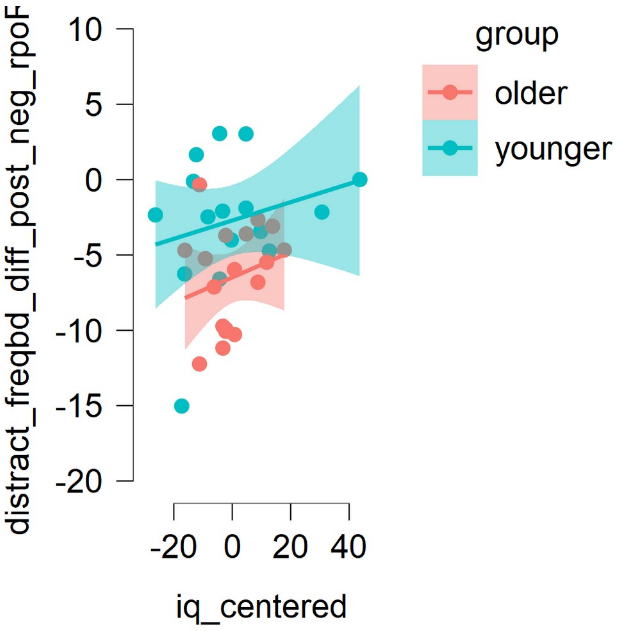

Frequent Go minus Distractor Go difference at the frontal ROI (anterior positivity)

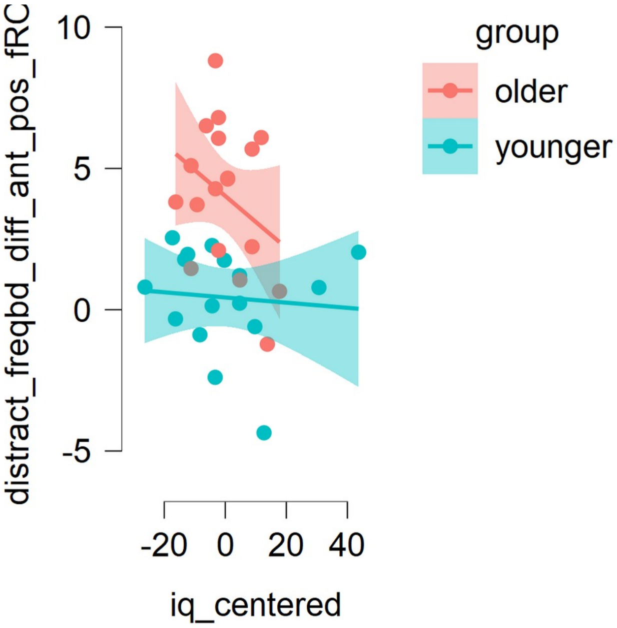

Frequent Go minus Distractor Go difference at the central ROI
(anterior positivity)

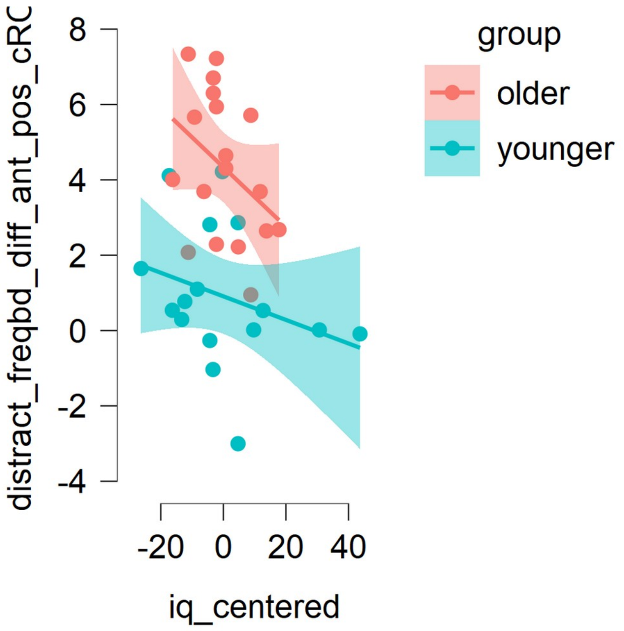

Nogo ERP at the parietal ROI
(Nogo N2)

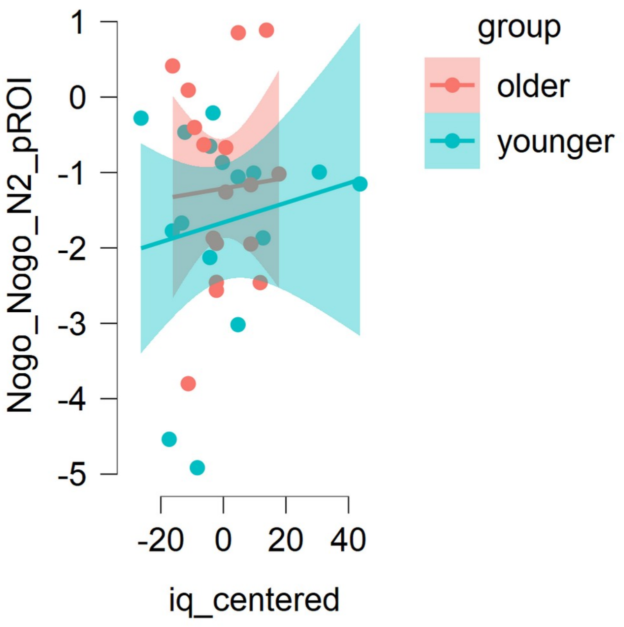

Nogo ERP at the central ROI
(Nogo P3)

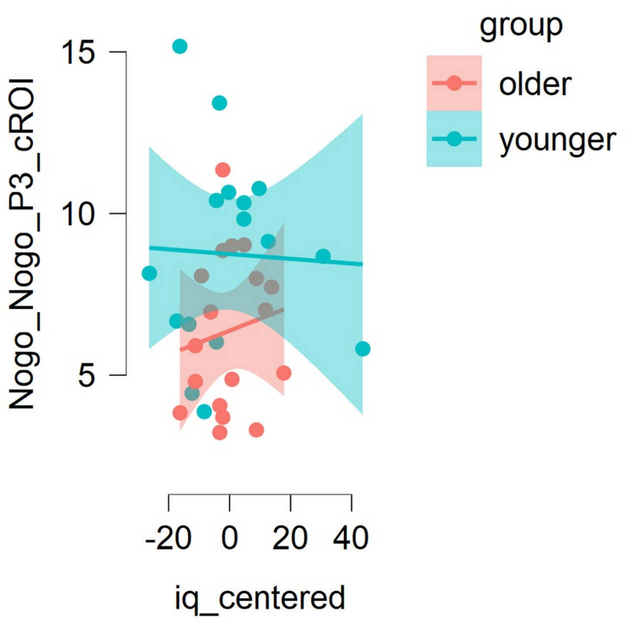

Nogo ERP at the parietal ROI
(Nogo P3)

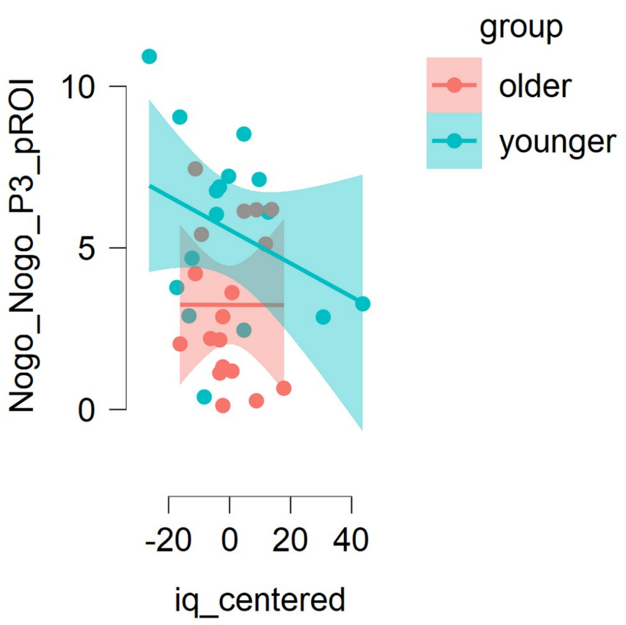

Peak-to-peak amplitude

Frequent Go minus Distractor Go difference at the frontal ROI
(N2b)

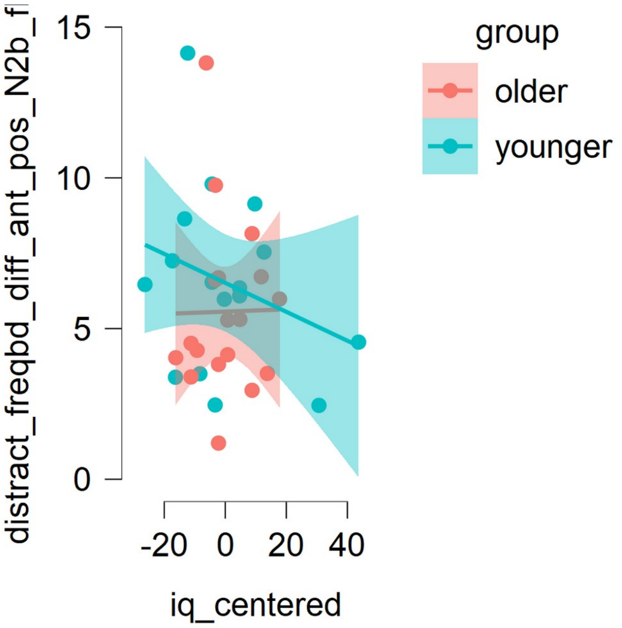

Frequent Go minus Distractor Go difference at the central ROI
(N2b)

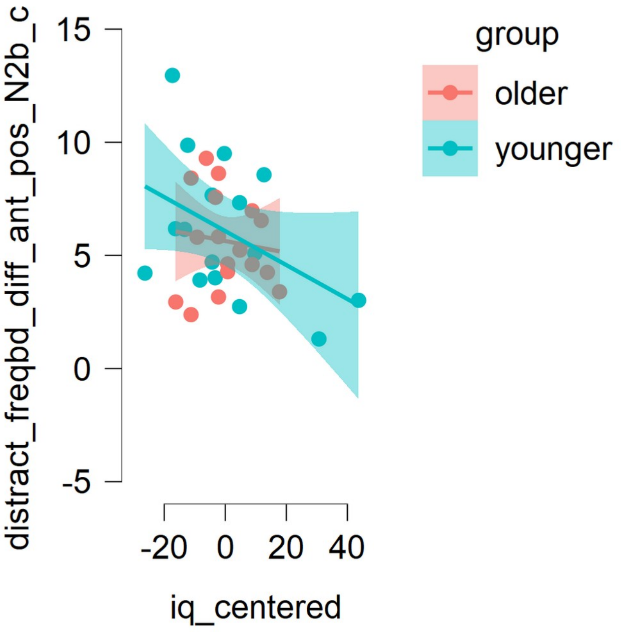

Supplement: Supplementary file 4 [file Data_Sheet_4.PDF]
