# Supplementary material for: Age Effects on Distraction in a Visual Task Requiring Fast Reactions: An Event-Related Potential Study
Source: Front Aging Neurosci. 2020 Nov 26;12:596047. doi: 10.3389/fnagi.2020.596047 (PMC7726357; doi:10.3389/fnagi.2020.596047)
Supplement: Supplementary file 5 [file Table_1.pdf]

Table 1. Brain regions in which the differences between the Frequent Go and Distractor Go trials were the largest for each experiment within each group. χ^2 is the largest χ^2-test value in the brain region, x, y, z are the MNI coordinates of the voxel with largest χ^2-test value, and N_{vx} is the number of voxels in the brain regions whose χ^2-test values are larger than mean + two standard deviations of all χ^2-test values. Average corrected p-threshold: 3.33289×10^{-6} (Bonferroni, *Ntests* = 15002). Only the time intervals for which the between-group comparisons yielded significant results are listed (see Table 2 below). Only regions for which there were at least 20 voxels in at least one of the groups and at least in one of the intervals are presented.

Experiment	Group	Time interval	Brain regions						
			Left fusiform	Left inferior parietal	Left inferior temporal	Left isthmus of the cingulate	Left lateral occipital	Left lingual	Left pericalcarine
Experiment 1	Younger group	150-168 ms					χ^2 = 6698.9<br>x, y, z = -54.1, -73.4, 11.1<br>N_{vx} = 5		
	Older group	130-148 ms	χ^2 = 21041.3<br>x, y, z = -28.5, -64.2, -8.1<br>N_{vx} = 12	χ^2 = 17330.5<br>x, y, z = -32.8, -92.5, 22.2<br>N_{vx} = 9			χ^2 = 24045.9<br>x, y, z = -12.4, -97.5, -18.4<br>N_{vx} = 40	χ^2 = 30772.4<br>x, y, z = -6.6, -75.3, 0.4<br>N_{vx} = 126	χ^2 = 28978.6<br>x, y, z = -12.4, -80.1, 6.2<br>N_{vx} = 45
		150-168 ms	χ^2 = 22475.4<br>x, y, z = -28.5, -64.2, -8.1<br>N_{vx} = 7				χ^2 = 23869.7<br>x, y, z = -12.4, -97.5, -18.4<br>N_{vx} = 24	χ^2 = 27603.6<br>x, y, z = -6.6, -75.3, 0.4<br>N_{vx} = 70	χ^2 = 22674.4<br>x, y, z = -12.7, -71.3, 6.2<br>N_{vx} = 11
Experiment 1a	Older group	130-148 ms	χ^2 = 11296.3<br>x, y, z = -26.6, -60.2, -10.5<br>N_{vx} = 25	χ^2 = 11652.8<br>x, y, z = -40.8, -90.8, 8.8<br>N_{vx} = 4		χ^2 = 11284.6<br>x, y, z = -4.8, -54.0, 6.0<br>N_{vx} = 10	χ^2 = 15966.7<br>x, y, z = -40.3, -92.9, -2.2<br>N_{vx} = 99	χ^2 = 13270.9<br>x, y, z = -21.2, -55.2, 0.9<br>N_{vx} = 185	χ^2 = 13159.8<br>x, y, z = -13.7, -72.8, 11.1<br>N_{vx} = 42
		150-168 ms	χ^2 = 11062.4<br>x, y, z = -24.1, -54.5, -11.1<br>N_{vx} = 2			χ^2 = 12857.6<br>x, y, z = -4.8, -54.0, 6.0<br>N_{vx} = 21	χ^2 = 11671.2<br>x, y, z = -26.4, -99.4, -16.5<br>N_{vx} = 3	χ^2 = 13968.1<br>x, y, z = -21.2, -55.2, 0.9<br>N_{vx} = 91	χ^2 = 11769.3<br>x, y, z = -12.9, -65.2, 7.9<br>N_{vx} = 17

Experiment	Group	Time interval	Brain regions						
			Left precuneus	Left superior frontal	Right cuneus	Right fusiform	Right inferior parietal	Right inferior temporal	Right isthmus of the cingulate
Experiment 2	Younger group	150-168 ms	χ^2 = 8267.0<br>x, y, z = -25.7, -72.7, -4.3<br>N_{vx} = 102	χ^2 = 7555.0<br>x, y, z = -40.5, -79.1, 13.4<br>N_{vx} = 11	χ^2 = 8772.2<br>x, y, z = -46.4, -68.8, 9.4<br>N_{vx} = 37		χ^2 = 17537.3<br>x, y, z = -42.1, -67.5, 8.8<br>N_{vx} = 84	χ^2 = 9774.2<br>x, y, z = -9.7, -91.2, -18.9<br>N_{vx} = 196	χ^2 = 7619.0<br>x, y, z = -25.7, -72.9, 5.9<br>N_{vx} = 31
		170-188 ms	χ^2 = 3593.4<br>x, y, z = -42.6, -77.0, -19.6<br>N_{vx} = 32	χ^2 = 4635.1<br>x, y, z = -43.9, -67.4, 13.7<br>N_{vx} = 50	χ^2 = 4265.7<br>x, y, z = -46.4, -68.8, 9.4<br>N_{vx} = 21		χ^2 = 5448.9<br>x, y, z = -42.1, -67.5, 8.8<br>N_{vx} = 82	χ^2 = 4167.5<br>x, y, z = -9.7, -91.2, -18.9<br>N_{vx} = 29	χ^2 = 3559.2<br>x, y, z = -25.7, -72.9, 5.9<br>N_{vx} = 10
	Older group	150-168 ms	χ^2 = 15468.1<br>x, y, z = -28.5, -70.4, -6.5<br>N_{vx} = 1	χ^2 = 17549.3<br>x, y, z = -29.8, -69.3, 27.2<br>N_{vx} = 8		χ^2 = 19224.2<br>x, y, z = -4.4, -46.2, 10.7<br>N_{vx} = 23	χ^2 = 17789.4<br>x, y, z = -18.1, -101.7, 20.5<br>N_{vx} = 13	χ^2 = 24350.2<br>x, y, z = -1.3, -73.0, 1.7<br>N_{vx} = 40	χ^2 = 16370.6<br>x, y, z = -2.9, -67.4, 10.6<br>N_{vx} = 6
		170-188 ms		χ^2 = 15988.0<br>x, y, z = -29.8, -69.3, 27.2<br>N_{vx} = 10		χ^2 = 14683.9<br>x, y, z = -2.7, -52.4, 16.7<br>N_{vx} = 15	χ^2 = 13781.0<br>x, y, z = -46.2, -68.8, 14.8<br>N_{vx} = 2		
Experiment 1	Younger group	150-168 ms			χ^2 = 7418.8<br>x, y, z = 9.6, -99.1, 0.8<br>N_{vx} = 12	χ^2 = 12684.2<br>x, y, z = 46.6, -57.6, -21.9<br>N_{vx} = 96	χ^2 = 20186.8<br>x, y, z = 42.3, -77.6, 14.1<br>N_{vx} = 100	χ^2 = 17462.8<br>x, y, z = 54.3, -60.8, -21.7<br>N_{vx} = 122	
	Older group	130-148 ms	χ^2 = 20513.3<br>x, y, z = -3.4, -58.0, 7.4<br>N_{vx} = 23		χ^2 = 28916.4<br>x, y, z = 13.4, -61.5, 18.3<br>N_{vx} = 24	χ^2 = 27814.2<br>x, y, z = 26.8, -67.1, -4.6<br>N_{vx} = 99	χ^2 = 19185.6<br>x, y, z = 33.7, -75.1, 20.3<br>N_{vx} = 55		χ^2 = 21732.1<br>x, y, z = 4.5, -52.4, 7.2<br>N_{vx} = 30
		150-168 ms	χ^2 = 20952.8<br>x, y, z = -3.4, -58.0, 7.4<br>N_{vx} = 6		χ^2 = 31647.7<br>x, y, z = 13.4, -61.5, 18.3<br>N_{vx} = 21	χ^2 = 33896.5<br>x, y, z = 26.8, -67.1, -4.6<br>N_{vx} = 105	χ^2 = 26862.2<br>x, y, z = 41.7, -70.8, 15.3<br>N_{vx} = 90		χ^2 = 24087.0<br>x, y, z = 12.2, -55.0, 5.7<br>N_{vx} = 31

Experiment 1a	Older group	130-148 ms	χ^2 = 12117.7<br>x, y, z = -8.8, -66.1, 20.6<br>N_{vx} = 29		χ^2 = 15671.3<br>x, y, z = 11.3, -94.4, 19.1<br>N_{vx} = 27	χ^2 = 22719.2<br>x, y, z = 23.5, -68.8, -6.8<br>N_{vx} = 61			χ^2 = 16393.7<br>x, y, z = 12.2, -55.0, 5.7<br>N_{vx} = 28
		150-168 ms	χ^2 = 14322.8<br>x, y, z = -2.0, -54.4, 15.4<br>N_{vx} = 28		χ^2 = 22797.0<br>x, y, z = 11.3, -94.4, 19.1<br>N_{vx} = 35	χ^2 = 24617.6<br>x, y, z = 23.5, -68.8, -6.8<br>N_{vx} = 61			χ^2 = 18913.1<br>x, y, z = 12.2, -55.0, 5.7<br>N_{vx} = 39
Experiment 2	Younger group	150-168 ms	χ^2 = 6572.7<br>x, y, z = -29.9, -63.6, 5.8<br>N_{vx} = 32			χ^2 = 6171.9<br>x, y, z = 24.6, -73.0, -16.2<br>N_{vx} = 35	χ^2 = 7470.0<br>x, y, z = 44.4, -86.6, 11.9<br>N_{vx} = 26		
		170-188 ms	χ^2 = 3388.7<br>x, y, z = -21.5, -66.6, 19.3<br>N_{vx} = 3	χ^2 = 7415.9<br>x, y, z = -7.3, 55.3, 44.7<br>N_{vx} = 24		χ^2 = 4003.0<br>x, y, z = 33.3, -76.1, -10.7<br>N_{vx} = 43	χ^2 = 5638.5<br>x, y, z = 44.4, -86.6, 11.9<br>N_{vx} = 32		
	Older group	150-168 ms	χ^2 = 19454.0<br>x, y, z = -3.0, -56.0, 8.3<br>N_{vx} = 18		χ^2 = 19334.7<br>x, y, z = 19.3, -61.2, 19.6<br>N_{vx} = 26	χ^2 = 15834.8<br>x, y, z = 34.1, -27.2, -21.3<br>N_{vx} = 16	χ^2 = 19942.1<br>x, y, z = 34.5, -88.9, 25.4<br>N_{vx} = 22		χ^2 = 19485.1<br>x, y, z = 4.5, -52.4, 7.2<br>N_{vx} = 38
		170-188 ms	χ^2 = 14803.0<br>x, y, z = -2.0, -54.4, 15.4<br>N_{vx} = 12		χ^2 = 20118.7<br>x, y, z = 19.3, -61.2, 19.6<br>N_{vx} = 31	χ^2 = 14899.3<br>x, y, z = 34.1, -27.2, -21.3<br>N_{vx} = 52	χ^2 = 22548.1<br>x, y, z = 49.2, -75.6, 16.8<br>N_{vx} = 42		χ^2 = 14969.2<br>x, y, z = 4.5, -52.4, 7.2<br>N_{vx} = 37

Experiment	Group	Time interval	Brain regions						
			Right lateral occipital	Right lingual	Right middle temporal	Right parahippocampal	Right pericalcarine	Right precuneus	Right superior parietal
Experiment 1	Younger group	150-168 ms	χ^2 = 17176.8<br>x, y, z = 46.6, -82.2, 11.9<br>N_{vx} = 241	χ^2 = 10929.3<br>x, y, z = 10.9, -98.5, -6.5<br>N_{vx} = 72	χ^2 = 16157.8<br>x, y, z = 63.3, -59.4, 15.7<br>N_{vx} = 94		χ^2 = 8959.5<br>x, y, z = 11.3, -98.1, 1.0<br>N_{vx} = 91	χ^2 = 7153.6<br>x, y, z = 29.5, -56.6, 8.0<br>N_{vx} = 12	χ^2 = 10799.7<br>x, y, z = 33.2, -74.6, 20.9<br>N_{vx} = 9
	Older group	130-148 ms	χ^2 = 29001.1<br>x, y, z = 39.8, -88.3, -17.2<br>N_{vx} = 171	χ^2 = 31142.7<br>x, y, z = 5.1, -91.3, -15.2<br>N_{vx} = 201			χ^2 = 25526.3<br>x, y, z = 27.9, -64.3, 7.5<br>N_{vx} = 40	χ^2 = 29232.2<br>x, y, z = 12.6, -56.2, 16.5<br>N_{vx} = 62	χ^2 = 19527.8<br>x, y, z = 29.1, -77.7, 18.3<br>N_{vx} = 19

		150-168 ms	χ^2 = 32339.5<br>x, y, z = 37.6, -78.0, 3.5<br>N_{vx} = 229	χ^2 = 35500.7<br>x, y, z = 27.0, -66.2, 4.3<br>N_{vx} = 200			χ^2 = 33547.1<br>x, y, z = 27.9, -64.3, 7.5<br>N_{vx} = 49	χ^2 = 32433.3<br>x, y, z = 12.6, -56.2, 16.5<br>N_{vx} = 59	χ^2 = 24830.9<br>x, y, z = 29.1, -77.7, 18.3<br>N_{vx} = 24
Experiment 1a	Older group	130-148 ms	χ^2 = 27921.4<br>x, y, z = 15.5, -99.5, 23.4<br>N_{vx} = 117	χ^2 = 24887.9<br>x, y, z = 22.4, -71.5, -3.7<br>N_{vx} = 209			χ^2 = 21559.3<br>x, y, z = 15.2, -91.3, 1.4<br>N_{vx} = 106	χ^2 = 15517.1<br>x, y, z = 16.2, -54.6, 6.2<br>N_{vx} = 40	
		150-168 ms	χ^2 = 37075.0<br>x, y, z = 13.2, -99.0, 21.8<br>N_{vx} = 96	χ^2 = 26579.0<br>x, y, z = 22.4, -71.5, -3.7<br>N_{vx} = 214			χ^2 = 20201.8<br>x, y, z = 15.2, -91.3, 1.4<br>N_{vx} = 88	χ^2 = 18197.0<br>x, y, z = 16.2, -54.6, 6.2<br>N_{vx} = 55	
Experiment 2	Younger group	150-168 ms	χ^2 = 7765.1<br>x, y, z = 45.5, -84.8, 8.8<br>N_{vx} = 90	χ^2 = 6882.2<br>x, y, z = 13.0, -88.6, -17.5<br>N_{vx} = 79			χ^2 = 5983.1<br>x, y, z = 16.3, -71.7, 11.9<br>N_{vx} = 13		χ^2 = 6182.0<br>x, y, z = 29.6, -80.7, 16.7<br>N_{vx} = 7
		170-188 ms	χ^2 = 6040.9<br>x, y, z = 44.6, -85.6, 5.9<br>N_{vx} = 124	χ^2 = 3517.9<br>x, y, z = 27.0, -66.2, 4.3<br>N_{vx} = 27	χ^2 = 3236.9<br>x, y, z = 56.8, -68.0, 4.4<br>N_{vx} = 7		χ^2 = 3534.6<br>x, y, z = 26.8, -67.0, 7.9<br>N_{vx} = 12		χ^2 = 4682.3<br>x, y, z = 29.6, -80.7, 16.7<br>N_{vx} = 14
	Older group	150-168 ms	χ^2 = 24259.2<br>x, y, z = 43.3, -84.8, 5.7<br>N_{vx} = 57	χ^2 = 25491.3<br>x, y, z = 2.4, -72.2, -0.5<br>N_{vx} = 105		χ^2 = 15971.2<br>x, y, z = 10.6, -38.3, -7.0<br>N_{vx} = 17	χ^2 = 17203.6<br>x, y, z = 21.1, -66.8, 5.5<br>N_{vx} = 26	χ^2 = 28515.6<br>x, y, z = 19.0, -60.0, 28.2<br>N_{vx} = 97	χ^2 = 21776.0<br>x, y, z = 28.7, -64.4, 32.8<br>N_{vx} = 12
		170-188 ms	χ^2 = 21502.2<br>x, y, z = 48.8, -74.9, 16.3<br>N_{vx} = 39	χ^2 = 15403.8<br>x, y, z = 16.1, -61.4, 7.6<br>N_{vx} = 58		χ^2 = 14732.2<br>x, y, z = 34.8, -28.0, -18.5<br>N_{vx} = 36	χ^2 = 15414.8<br>x, y, z = 10.3, -61.2, 10.2<br>N_{vx} = 30	χ^2 = 30606.6<br>x, y, z = 19.0, -60.0, 28.2<br>N_{vx} = 118	χ^2 = 24148.4<br>x, y, z = 28.7, -64.4, 32.8<br>N_{vx} = 15
