# Supplementary material for: Age Effects on Distraction in a Visual Task Requiring Fast Reactions: An Event-Related Potential Study
Source: Front Aging Neurosci. 2020 Nov 26;12:596047. doi: 10.3389/fnagi.2020.596047 (PMC7726357; doi:10.3389/fnagi.2020.596047)
Supplement: Supplementary file 6 [file Table_2.pdf]

Table 2. Brain regions in which there were significant Distractor Go *minus* Frequent Go differences between the groups for each experiment. *t* is the largest value of the *t*-tests in the brain region, *p* is the *p* value for the largest *t*-value, x, y, z are the MNI coordinates of the voxel with largest *t*-value, and N_{vx} is the number of voxels in the brain region with significant differences. Only the time intervals for which the between-group comparisons yielded significant results are listed. Only regions for which there were at least 20 voxels in at least one of the comparisons and at least in one of the intervals are presented.

Experiment	Comparison	Time interval	Brain regions				
			Left banks of superior, temporal sulcus	Left caudal middle frontal	Left cuneus	Left entorhinal	Left fusiform
Experiment 1	Younger group vs. Older group	150-168 ms	t = -3.38<br>p = 0.002<br>x, y, z = -48.6, -43.7, 0.7<br>N_{vx} = 20	t = -3.66<br>p = 0.001<br>x, y, z = -26.1, -6.2, 52.2<br>N_{vx} = 31	t = -4.46<br>p < 0.001<br>x, y, z = -2.7, -70.1, 20.7<br>N_{vx} = 70	t = -3.59<br>p = 0.001<br>x, y, z = -20.7, -14.5, -32.6<br>N_{vx} = 25	t = -5.02<br>p < 0.001<br>x, y, z = -23.8, -71.5, -14.9<br>N_{vx} = 254
Experiment 1a	Experiment 1 vs. Experiment 1a (Older group)	130-148 ms					
		150-168 ms	t = 3.56<br>p = 0.001<br>x, y, z = -63.5, -51.5, 12.3<br>N_{vx} = 1				
Experiment 2	Younger group vs. Older group	150-168 ms			t = -5.14<br>p < 0.001<br>x, y, z = -0.1, -86.1, 30.3<br>N_{vx} = 22		
		170-188 ms			t = -4.66<br>p < 0.001<br>x, y, z = -0.9, -84.6, 27.8<br>N_{vx} = 50		

Experiment	Comparison	Time interval	Brain regions				
			Left inferior parietal	Left inferior temporal	Left isthmus of the cingulate	Left lateral occipital	Left lingual
Experiment 1	Younger group vs. Older group	150-168 ms	$t = -4.47$<br>$p < 0.001$<br>x, y, z = -30.6, -91.9, 30.1<br>$N_{vx} = 121$	$t = -4.35$<br>$p < 0.001$<br>x, y, z = -51.5, -46.2, -28.4<br>$N_{vx} = 226$	$t = -6.33$<br>$p < 0.001$<br>x, y, z = -1.4, -31.4, 28.1<br>$N_{vx} = 81$	$t = -4.35$<br>$p < 0.001$<br>x, y, z = -36.9, -93.0, -12.5<br>$N_{vx} = 269$	$t = -5.51$<br>$p < 0.001$<br>x, y, z = -13.2, -46.2, -8.0<br>$N_{vx} = 242$
Experiment 1a	Experiment 1 vs. Experiment 1a (Older group)	130-148 ms		$t = 3.40$<br>$p = 0.001$<br>x, y, z = -65.7, -37.3, -15.4<br>$N_{vx} = 47$			
		150-168 ms		$t = 3.93$<br>$p = 0.001$<br>x, y, z = -67.4, -39.9, -16.1<br>$N_{vx} = 3$			
Experiment 2	Younger group vs. Older group	150-168 ms	$t = -4.98$<br>$p < 0.001$<br>x, y, z = -29.0, -85.7, 30.8<br>$N_{vx} = 32$			$t = -4.22$<br>$p < 0.001$<br>x, y, z = -13.7, -96.7, 30.7<br>$N_{vx} = 13$	
		170-188 ms	$t = -4.82$<br>$p < 0.001$<br>x, y, z = -30.6, -91.9, 30.1<br>$N_{vx} = 22$			$t = -4.50$<br>$p = 0.001$<br>x, y, z = -3.6, -104.6, 9.2<br>$N_{vx} = 99$	$t = -4.01$<br>$p = 0.001$<br>x, y, z = -0.4, -89.0, -0.7<br>$N_{vx} = 11$

Experiment	Comparison	Time interval	Brain regions				
			Left middle temporal	Left paracentral	Left parahippocampal	Left pars orbitalis	Left pericalcarine
Experiment 1	Younger group vs. Older group	150-168 ms	$t = -3.96$<br>$p < 0.001$<br>x, y, z = -53.9, 4.0, -42.2<br>$N_{vx} = 144$	$t = -6.70$<br>$p < 0.001$<br>x, y, z = -2.4, -15.2, 48.6<br>$N_{vx} = 110$	$t = -5.50$<br>$p < 0.001$<br>x, y, z = -10.4, -41.3, -6.5<br>$N_{vx} = 68$	$t = -3.35$<br>$p = 0.002$<br>x, y, z = -47.7, 22.8, -16.6<br>$N_{vx} = 16$	$t = -4.18$<br>$p < 0.001$<br>x, y, z = -0.9, -68.7, 13.3<br>$N_{vx} = 77$

Experiment 1a	Experiment 1 vs. Experiment 1a (Older group)	130-148 ms	*t* = 3.67<br>*p* < 0.001<br>x, y, z = -65.4, -12.7, -8.7<br>N_{vx} = 50				
		150-168 ms	*t* = 3.92<br>*p* = 0.001<br>x, y, z = -66.9, -40.8, -15.7<br>N_{vx} = 17				
Experiment 2	Younger group vs. Older group	150-168 ms					
		170-188 ms					*t* = -4.05<br>*p* = 0.001<br>x, y, z = -1.5, -94.7, -3.4<br>N_{vx} = 14

Experiment	Comparison	Time interval	Brain regions				
			Left postcentral	Left posterior cingulate	Left precentral	Left precuneus	Left superior frontal
Experiment 1	Younger group vs. Older group	150-168 ms	*t* = -4.61<br>*p* < 0.001<br>x, y, z = -28.9, -28.0, 65.7<br>N_{vx} = 89	*t* = -6.65<br>*p* < 0.001<br>x, y, z = -1.4, -14.9, 45.1<br>N_{vx} = 85	*t* = -4.75<br>*p* < 0.001<br>x, y, z = -23.9, -12.5, 60.4<br>N_{vx} = 125	*t* = -6.17<br>*p* < 0.001<br>x, y, z = -1.0, -37.9, 39.8<br>N_{vx} = 247	*t* = -6.78<br>*p* < 0.001<br>x, y, z = -1.0, -13.1, 50.5<br>N_{vx} = 179
Experiment 1a	Experiment 1 vs. Experiment 1a (Older group)	130-148 ms					
		150-168 ms					

Experiment 2	Younger group vs. Older group	150-168 ms				t = -3.97<br>p < 0.001<br>x, y, z = -17.5, -78.2, 36.3<br>N_{vx} = 46	
		170-188 ms				t = -3.99<br>p = 0.00<br>x, y, z = -14.8, -79.2, 28.3<br>N_{vx} = 99	

Experiment	Comparison	Time interval	Brain regions				
			Left superior parietal	Left superior temporal	Right banks of superior, temporal sulcus	Right caudal middle frontal	Right cuneus
Experiment 1	Younger group vs. Older group	150-168 ms	t = -4.48<br>p < 0.001<br>x, y, z = -26.5, -91.1, 31.4<br>N_{vx} = 227	t = -3.77<br>p = 0.001<br>x, y, z = -42.7, 18.4, -39.7<br>N_{vx} = 118	t = -3.38<br>p = 0.002<br>x, y, z = 46.0, -44.9, 19.9<br>N_{vx} = 4	t = -5.13<br>p < 0.000<br>x, y, z = 26.4, -5.4, 51.3<br>N_{vx} = 37	t = -4.64<br>p < 0.001<br>x, y, z = 1.2, -66.9, 17.8<br>N_{vx} = 72
Experiment 1a	Experiment 1 vs. Experiment 1a (Older group)	130-148 ms		t = 3.67<br>p = 0.001<br>x, y, z = -66.2, -12.6, -7.0<br>N_{vx} = 26	t = 3.75<br>p < 0.001<br>x, y, z = 54.8, -46.8, 18.0<br>N_{vx} = 59		
		150-168 ms		t = 3.62<br>p < 0.001<br>x, y, z = -64.1, -56.1, 13.7<br>N_{vx} = 3	t = 4.21<br>p < 0.001<br>x, y, z = 61.3, -43.5, 8.2<br>N_{vx} = 59		
Experiment 2	Younger group vs. Older group	150-168 ms	t = -5.19<br>p < 0.001<br>x, y, z = -19.3, -88.3, 44.4<br>N_{vx} = 91				t = -4.66<br>p < 0.001<br>x, y, z = 3.3, -86.3, 34.1<br>N_{vx} = 13

		170-188 ms	$t = -5.04$<br>$p < 0.001$<br>x, y, z = -10.7, -91.8, 39.4<br>$N_{vx} = 86$		$t = -3.84$<br>$p = 0.002$<br>x, y, z = 46.0, -44.9, 19.9<br>$N_{vx} = 2$	$t = 3.27$<br>$p = 0.001$<br>x, y, z = 46.3, 20.3, 40.9<br>$N_{vx} = 8$	$t = -4.28$<br>$p < 0.001$<br>x, y, z = 2.0, -86.6, 25.8<br>$N_{vx} = 66$

Experiment	Comparison	Time interval	Brain regions				
			Right entorhinal	Right frontal pole	Right fusiform	Right inferior parietal	Right inferior temporal
Experiment 1	Younger group vs. Older group	150-168 ms	$t = -3.93$<br>$p < 0.001$<br>x, y, z = 30.5, -3.9, -34.6<br>$N_{vx} = 32$		$t = -4.69$<br>$p < 0.001$<br>x, y, z = 22.2, -39.0, -17.2<br>$N_{vx} = 185$	$t = -4.26$<br>$p < 0.001$<br>x, y, z = 32.8, -83.0, 32.9<br>$N_{vx} = 228$	$t = -4.64$<br>$p < 0.001$<br>x, y, z = 48.0, -2.1, -33.6<br>$N_{vx} = 100$
Experiment 1a	Experiment 1 vs. Experiment 1a (Older group)	130-148 ms				$t = 4.19$<br>$p < 0.001$<br>x, y, z = 63.9, -56.2, 19.9<br>$N_{vx} = 115$	$t = 4.09$<br>$p < 0.001$<br>x, y, z = 61.6, -12.4, -30.6<br>$N_{vx} = 35$
		150-168 ms	$t = 3.77$<br>$p = 0.001$<br>x, y, z = 29.6, -0.6, -36.1<br>$N_{vx} = 3$	$t = 4.12$<br>$p < 0.001$<br>x, y, z = 9.0, 66.1, -23.5<br>$N_{vx} = 23$		$t = 4.63$<br>$p < 0.001$<br>x, y, z = 62.8, -56.8, 18.0<br>$N_{vx} = 98$	$t = 4.33$<br>$p < 0.001$<br>x, y, z = 63.2, -14.3, -32.7<br>$N_{vx} = 73$
Experiment 2	Younger group vs. Older group	150-168 ms				$t = -5.13$<br>$p < 0.001$<br>x, y, z = 47.6, -58.6, 57.7<br>$N_{vx} = 78$	
		170-188 ms			$t = -4.21$<br>$p < 0.001$<br>x, y, z = 42.2, -32.3, -19.4<br>$N_{vx} = 61$	$t = -4.96$<br>$p < 0.001$<br>x, y, z = 41.6, -59.9, 58.5<br>$N_{vx} = 238$	$t = -4.21$<br>$p < 0.001$<br>x, y, z = 43.9, -31.9, -23.1<br>$N_{vx} = 31$

Experiment	Comparison	Time interval	Brain regions				
			Right insula	Right isthmus of the cingulate	Right lateral occipital	Right lateral orbitofrontal	Right lingual
Experiment 1	Younger group vs. Older group	150-168 ms	$t = -4.79$<br>$p < 0.001$<br>x, y, z = 40.6, 4.5, -17.6<br>$N_{vx} = 95$	$t = -6.52$<br>$p < 0.001$<br>x, y, z = 2.6, -34.6, 36.5<br>$N_{vx} = 91$	$t = -4.65$<br>$p < 0.001$<br>x, y, z = 19.0, -86.5, 23.8<br>$N_{vx} = 183$	$t = -3.77$<br>$p = 0.001$<br>x, y, z = 30.0, 15.8, -24.0<br>$N_{vx} = 35$	$t = -5.36$<br>$p < 0.001$<br>x, y, z = 9.8, -45.0, -2.6<br>$N_{vx} = 219$
Experiment 1a	Experiment 1 vs. Experiment 1a (Older group)	130-148 ms	$t = 5.59$<br>$p < 0.001$<br>x, y, z = 45.3, 11.5, -8.6<br>$N_{vx} = 161$		$t = 3.74$<br>$p < 0.001$<br>x, y, z = 57.7, -68.3, 14.6<br>$N_{vx} = 7$	$t = 5.14$<br>$p < 0.001$<br>x, y, z = 40.2, 20.2, -13.5<br>$N_{vx} = 109$	
		150-168 ms	$t = 5.20$<br>$p < 0.001$<br>x, y, z = 44.7, 16.2, -8.8<br>$N_{vx} = 157$		$t = 4.13$<br>$p < 0.001$<br>x, y, z = 31.7, -82.6, 17.5<br>$N_{vx} = 42$	$t = 4.97$<br>$p < 0.001$<br>x, y, z = 40.2, 20.2, -13.5<br>$N_{vx} = 181$	
Experiment 2	Younger group vs. Older group	150-168 ms			$t = -4.35$<br>$p < 0.001$<br>x, y, z = 28.7, -100.8, -3.4<br>$N_{vx} = 22$		
		170-188 ms	$t = -3.90$<br>$p = 0.001$<br>x, y, z = 33.1, -26.6, 18.2<br>$N_{vx} = 17$	$t = -4.19$<br>$p = 0.001$<br>x, y, z = 8.5, -51.8, 3.9<br>$N_{vx} = 33$	$t = -4.26$<br>$p < 0.001$<br>x, y, z = 7.9, -102.3, 9.2<br>$N_{vx} = 106$		$t = -4.20$<br>$p = 0.001$<br>x, y, z = 7.6, -53.9, 2.5<br>$N_{vx} = 87$

Experiment	Comparison	Time interval	Brain regions				
			Right medial orbitofrontal	Right middle temporal	Right paracentral	Right parahippocampal	Right pars opercularis
Experiment 1	Younger group vs. Older group	150-168 ms		t = -4.89<br>p < 0.001<br>x, y, z = 49.7, 6.0, -28.3<br>N_{vx} = 126	t = -6.93<br>p = 0.001<br>x, y, z = 4.8, -12.4, 47.9<br>N_{vx} = 124	t = -5.36<br>p < 0.001<br>x, y, z = 11.4, -35.7, -7.8<br>N_{vx} = 66	t = -3.94<br>p < 0.001<br>x, y, z = 50.2, 13.7, -4.7<br>N_{vx} = 10
Experiment 1a	Experiment 1 vs. Experiment 1a (Older group)	130-148 ms		t = 4.65<br>p < 0.001<br>x, y, z = 64.2, -6.0, -10.0<br>N_{vx} = 130			t = 5.70<br>p < 0.001<br>x, y, z = 50.2, 13.7, -4.7<br>N_{vx} = 38
		150-168 ms	t = 3.90<br>p < 0.001<br>x, y, z = 9.4, 50.1, -13.7<br>N_{vx} = 56	t = 4.62<br>p < 0.001<br>x, y, z = 67.5, -7.2, -20.0<br>N_{vx} = 173			t = 5.30<br>p < 0.001<br>x, y, z = 50.3, 17.7, -3.9<br>N_{vx} = 72
Experiment 2	Younger group vs. Older group	150-168 ms			t = -3.92<br>p < 0.001<br>x, y, z = 13.8, -42.5, 60.0<br>N_{vx} = 5		
		170-188 ms		t = -3.75<br>p = 0.001<br>x, y, z = 46.7, -33.9, -1.2<br>N_{vx} = 4	t = -4.33<br>p < 0.001<br>x, y, z = 15.9, -41.8, 50.8<br>N_{vx} = 29	t = -4.17<br>p = 0.001<br>x, y, z = 35.5, -32.0, -16.0<br>N_{vx} = 31	

Experiment	Comparison	Time interval	Brain regions				
			Right pars orbitalis	Right pars triangularis	Right pericalcarine	Right postcentral	Right posterior cingulate
Experiment 1	Younger group vs. Older group	150-168 ms	$t = -3.83$<br>$p = 0.001$<br>x, y, z = 43.6, 20.5, -13.6<br>$N_{vx} = 8$	$t = -3.65$<br>$p = 0.001$<br>x, y, z = 51.4, 22.5, -7.6<br>$N_{vx} = 7$	$t = -4.00$<br>$p < 0.001$<br>x, y, z = 14.7, -62.9, 7.7<br>$N_{vx} = 49$	$t = -5.61$<br>$p < 0.001$<br>x, y, z = 24.5, -26.5, 55.6<br>$N_{vx} = 152$	$t = -6.97$<br>$p < 0.001$<br>x, y, z = 2.5, -15.7, 47.5<br>$N_{vx} = 93$
Experiment 1a	Experiment 1 vs. Experiment 1a (Older group)	130-148 ms	$t = 5.44$<br>$p < 0.001$<br>x, y, z = 45.5, 23.2, -9.3<br>$N_{vx} = 39$	$t = 5.63$<br>$p < 0.001$<br>x, y, z = 49.1, 21.8, -7.2<br>$N_{vx} = 38$		$t = 4.62$<br>$p < 0.001$<br>x, y, z = 48.6, -8.0, 6.9<br>$N_{vx} = 52$	
		150-168 ms	$t = 5.24$<br>$p < 0.001$<br>x, y, z = 45.5, 23.2, -9.3<br>$N_{vx} = 68$	$t = 5.33$<br>$p < 0.001$<br>x, y, z = 49.1, 21.8, -7.2<br>$N_{vx} = 115$		$t = 4.07$<br>$p < 0.001$<br>x, y, z = 48.6, -8.0, 6.9<br>$N_{vx} = 15$	
Experiment 2	Younger group vs. Older group	150-168 ms					
		170-188 ms			$t = -4.05$<br>$p = 0.001$<br>x, y, z = 6.5, -93.8, 1.2<br>$N_{vx} = 50$	$t = -4.53$<br>$p < 0.001$<br>x, y, z = 27.7, -39.1, 52.3<br>$N_{vx} = 24$	$t = -4.02$<br>$p = 0.001$<br>x, y, z = 15.3, -35.0, 41.5<br>$N_{vx} = 7$

Experiment	Comparison	Time interval	Brain regions				
			Right precentral	Right precuneus	Right rostral anterior cingulate	Right rostral middle frontal	Right superior frontal
Experiment 1	Younger group vs. Older group	150-168 ms	$t = -5.67$<br>$p < 0.001$<br>x, y, z = 22.3, -25.5, 56.2<br>$N_{vx} = 205$	$t = -6.21$<br>$p < 0.001$<br>x, y, z = 8.2, -37.1, 39.4<br>$N_{vx} = 261$			$t = -6.15$<br>$p < 0.001$<br>x, y, z = 6.5, -7.2, 44.8<br>$N_{vx} = 145$
Experiment 1a	Experiment 1 vs. Experiment 1a (Older group)	130-148 ms	$t = 5.05$<br>$p < 0.001$<br>x, y, z = 55.2, 9.4, -1.4<br>$N_{vx} = 48$			$t = 3.63$<br>$p = 0.00$<br>x, y, z = 18.6, 59.8, -20.9<br>$N_{vx} = 2$	
		150-168 ms	$t = 4.71$<br>$p < 0.001$<br>x, y, z = 47.5, 7.2, -2.8<br>$N_{vx} = 46$		$t = 3.78$<br>$p = 0.001$<br>x, y, z = 11.3, 42.0, -2.1<br>$N_{vx} = 14$	$t = 4.19$<br>$p < 0.001$<br>x, y, z = 33.8, 56.5, -18.4<br>$N_{vx} = 105$	$t = 3.73$<br>$p = 0.001$<br>x, y, z = 14.1, 46.6, 2.0<br>$N_{vx} = 22$
Experiment 2	Younger group vs. Older group	150-168 ms		$t = -3.99$<br>$p < 0.001$<br>x, y, z = 2.6, -71.9, 54.4<br>$N_{vx} = 26$		$t = 4.50$<br>$p < 0.001$<br>x, y, z = 42.8, 34.3, 39.2<br>$N_{vx} = 52$	$t = 3.51$<br>$p = 0.001$<br>x, y, z = 25.5, 44.0, 42.6<br>$N_{vx} = 189$
		170-188 ms		$t = -4.30$<br>$p < 0.001$<br>x, y, z = 15.6, -41.3, 48.9<br>$N_{vx} = 143$		$t = 3.27$<br>$p = 0.001$<br>x, y, z = 46.3, 24.2, 41.6<br>$N_{vx} = 5$	$t = 2.48$<br>$p = 0.017$<br>x, y, z = 25.5, 35.0, 48.8<br>$N_{vx} = 325$

Experiment	Comparison	Time interval	Brain regions				
			Right superior parietal	Right superior temporal	Right supramarginal	Right temporal pole	Right transverse temporal
Experiment 1	Younger group vs. Older group	150-168 ms	*t* = -5.29<br>*p* < 0.001<br>x, y, z = 37.3, -38.2, 58.4<br>N_{vx} = 350	*t* = -4.92<br>*p* < 0.001<br>x, y, z = 47.5, 4.6, -25.7<br>N_{vx} = 139	*t* = -4.11<br>*p* < 0.001<br>x, y, z = 43.3, -35.4, 53.8<br>N_{vx} = 47	*t* = -4.08<br>*p* < 0.001<br>x, y, z = 31.4, 11.7, -28.4<br>N_{vx} = 38	*t* = -3.34<br>*p* = 0.002<br>x, y, z = 48.5, -6.3, 1.0<br>N_{vx} = 1
Experiment 1a	Experiment 1 vs. Experiment 1a (Older group)	130-148 ms	*t* = 3.44<br>*p* = 0.001<br>x, y, z = 31.2, -78.2, 21.0<br>N_{vx} = 5	*t* = 5.81<br>*p* < 0.001<br>x, y, z = 50.3, 14.6, -7.4<br>N_{vx} = 206	*t* = 4.20<br>*p* < 0.001<br>x, y, z = 60.0, -46.8, 27.5<br>N_{vx} = 102	*t* = 4.20<br>*p* = 0.001<br>x, y, z = 31.4, 11.7, -28.4<br>N_{vx} = 18	*t* = 5.07<br>*p* < 0.001<br>x, y, z = 48.5, -6.3, 1.0<br>N_{vx} = 21
		150-168 ms	*t* = 4.15<br>*p* < 0.001<br>x, y, z = 29.1, -77.7, 18.3<br>N_{vx} = 17	*t* = 5.29<br>*p* < 0.001<br>x, y, z = 50.3, 14.6, -7.4<br>N_{vx} = 143	*t* = 4.01<br>*p* < 0.001<br>x, y, z = 61.4, -51.2, 20.8<br>N_{vx} = 13	*t* = 4.24<br>*p* < 0.001<br>x, y, z = 31.4, 11.7, -28.4<br>N_{vx} = 31	*t* = 4.60<br>*p* < 0.001<br>x, y, z = 48.5, -6.3, 1.0<br>N_{vx} = 7
Experiment 2	Younger group vs. Older group	150-168 ms			*t* = -4.71<br>*p* < 0.001<br>x, y, z = 46.4, -48.6, 54.1<br>N_{vx} = 25		
		170-188 ms	*t* = -4.98<br>*p* < 0.001<br>x, y, z = 37.5, -58.0, 63.5<br>N_{vx} = 11		*t* = -4.37<br>*p* = 0.001<br>x, y, z = 41.6, -33.4, 20.6<br>N_{vx} = 84		*t* = -3.91<br>*p* < 0.001<br>x, y, z = 41.7, -26.5, 15.4<br>N_{vx} = 5
